# Synthesis of 2-Amino-*N*′-aroyl(het)arylhydrazides, DNA Photocleavage, Molecular Docking and Cytotoxicity Studies against Melanoma CarB Cell Lines

**DOI:** 10.3390/molecules29030647

**Published:** 2024-01-30

**Authors:** Achilleas Mitrakas, Maria-Eleni K. Stathopoulou, Chrysoula Mikra, Chrystalla Konstantinou, Stergios Rizos, Stella Malichetoudi, Alexandros E. Koumbis, Maria Koffa, Konstantina C. Fylaktakidou

**Affiliations:** 1 Laboratory of Cellular Biology, Molecular Biology and Genetics Department, Democritus University of Thrace, University Campus, 68100 Alexandroupolis, Greece; amitrak@med.duth.gr (A.M.); stelmali1@mbg.duth.gr (S.M.); mkoffa@mbg.duth.gr (M.K.); 2 Laboratory of Organic, Bioorganic and Natural Product Chemistry, Molecular Biology and Genetics Department, Democritus University of Thrace, 68100 Alexandroupolis, Greece; marialenastathopoulou@outlook.com (M.-E.K.S.); chrystallakon@outlook.com (C.K.); 3 Laboratory of Organic Chemistry, Faculty of Chemistry, Aristotle University of Thessaloniki, 54124 Thessaloniki, Greece; chrmikgeo@chem.auth.gr (C.M.); akoumbis@chem.auth.gr (A.E.K.); 4 Department of Chemistry and Chemical Biology, Harvard University, 12 Oxford St., Cambridge, MA 02138, USA; stergiosrizos@fas.harvard.edu

**Keywords:** diacylhydrazine, anthranilic acid, DNA photocleavers, molecular dockings

## Abstract

Diacylhydrazine bridged anthranilic acids with aryl and heteroaryl domains have been synthesized as the open flexible scaffold of arylamide quinazolinones in order to investigate flexibility versus rigidity towards DNA photocleavage and sensitivity. Most of the compounds have been synthesized via the in situ formation of their anthraniloyl chloride and subsequent reaction with the desired hydrazide and were obtained as precipitates, in moderate yields. All compounds showed high UV-A light absorption and are eligible for DNA photocleavage studies under this “harmless” irradiation. Despite their reduced UV-B light absorption, a first screening indicated the necessity of a halogen at the *p*-position in relation to the amine group and the lack of an electron-withdrawing group on the aryl group. These characteristics, in general, remained under UV-A light, rendering these compounds as a novel class of UV-A-triggered DNA photocleavers. The best photocleaver, the compound **9**, was active at concentrations as low as 2 μΜ. The 5-Nitro-anthranilic derivatives were inactive, giving the opposite results to their related rigid quinazolinones. Molecular docking studies with DNA showed possible interaction sites, whereas cytotoxicity experiments indicated the iodo derivative **17** as a potent cytotoxic agent and the compound **9** as a slight phototoxic compound.

## 1. Introduction

**Figure 1:**
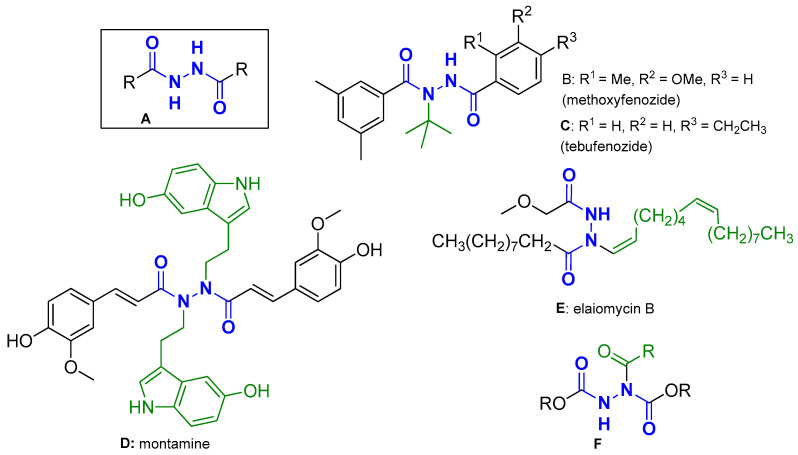
**A:** General structure of DACHZs; **B**, **C:** commercially available insecticides bearing a DACHZ moiety; **D**, **E:** natural products bearing a DACHZ group; **F:** a DACHZ-related motif with synthon characteristics. Blue color: the DACHZ skeleton; Green color: Substituents other than H on the nitrogen atom.

*N*,*N*′-Diacylhydrazines (DACHZs, general structure **A**, Figure 1) represent an interesting class of compounds with potential applications in chemistry and biology. Several *N*-*tert*-butyl-substituted derivatives, such as methoxyfenozide and tebufenozide (**B** and **C**, respectively, Figure 1), are well known for their insecticidal activity. Due to their ability to mimic the action of two principal hormones in insects while having almost no impact on most non-target and beneficiary organisms, those derivatives have been subjected to numerous structural modifications [1,2,3,4]. Moreover, an *N*,*N*′-bis substituted DACHZ moiety is found in the naturally occurring alkaloids montamine (**D**) that displays anti-oxidative activity and cytotoxicity against CaCo-2 colon cancer (IC_50_ = 43.9 μΜ) and elaiοmycin (**E**) that displays strong in vitro inhibition against bovine and human strains of *Mycobacterium tuberculosis* [3,5]. A similar urethan-like pattern (general structure **F**) is considered as a useful synthon in Organic Chemistry [6].

**Figure 2:**
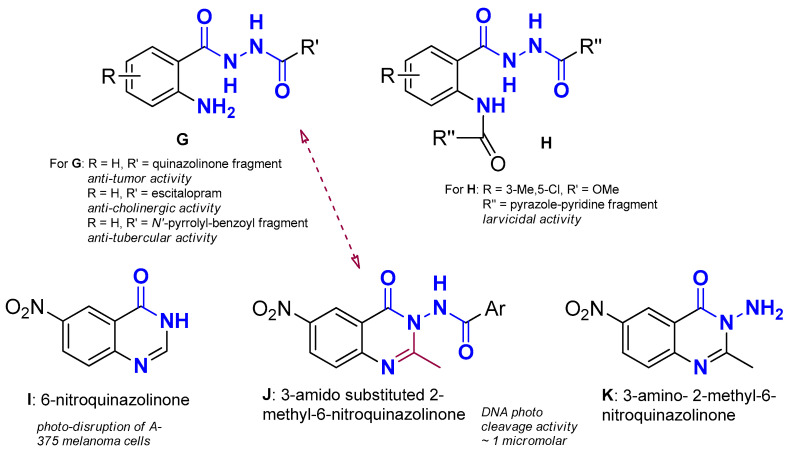
**G**, **H:** Structures of non-substituted (**G**) [31,33,34] or arylamine substituted (**H**) AA DACHZs [36]; **I**, **J** and **K:** Structures of known DNA photocleaving nitro-quinazolinone derivatives [57,58]; **G** and **J** are the open flexible and closed rigid structures corresponding to AA DACHZ and quinazolinone (red arrow between **G** and **J** as well as red color on **J** indicate similarities and differences between the two scaffolds).

Anthranilic acid DACHZs (AA DACHZs, Figure 2, **G**, **H**) bear an additional functional amine group which contributes to their chemistry, biology and technology. Derivatives such as **G** may give rise to 1,3,4-oxadiazoles [7,8,9,10], 1,2,3-benzotriazine-4-ones [11], 3-amido-1,2-dihydro substituted quinazolinones [12,13,14,15,16,17], 3-amido substituted quinazolinones [10,18], quinazolinone containing fused polycyclic compounds [19,20] or *N*-*N* axially chiral 3,3′-bisquinazolinones [21]. Furthermore, their well-positioned electron-donating atoms allow metal complexation which, depending on the metal and the conditions applied, range from the formation of simple metal complexes [22,23,24] to the important metal–organic macrocycles (MOMs) [25,26] with Mn, Ga and In [27,28,29]. Compounds of the general structures **G** and **H** have also been used as conjugates of the chemotherapeutic drug daunorubicin [30], whereas other AA DACHZs have been tested or were found to exhibit biological activity as inhibitors of EGFR [31], of HIV-1 Integrase [32], of cholinesterase [33], of enoyl ACP reductase [34] or as potent insecticidal agents that target ryanodine receptors [35,36] (Figure 2: for some structures, the R and R′ or/and R″ are defined, along with the biological activities of the compounds, below the general structure).

Organic DNA photocleavers that can be photosensitized under UV-A irradiation are less studied compared to organometallic compounds, because the majority of the former compounds lack strong absorptions above the UV-B light wavelength. However, the interest for small organic molecules that can photocleave DNA [37] is showing a come-back due to the need for alternative anticancer and antimicrobial therapies able to overcome drug resistances [38,39,40,41]. Thus, pyrazoles (UV-A), trifluoromethyl pyrazolines and pyrazoles (UV-B), as well as bis-pyranopyrazoles (UV-A), were found to photocleave DNA exhibiting additional antibacterial, cytotoxic and antimicrobial activities, respectively [42,43,44]. Interestingly, halogenated derivatives were the best photocleavers among pyrazoles [42], as well as nitro-substituted trifluoromethyl pyrazoles [43]. Deazaflavin analogs linked to the naphthalene core (UV-A) [45] were also found to photocleave DNA, and quinolinium dicarbocyanine dyes (near IR) bearing a pentamethine bridge that was meso-substituted with halogen caused photodynamic cell damage [46,47]. The attachment of a halogen (Cl or Br) at the polymethine meso-carbon was anticipated to introduce a “heavy atom effect” in which ROS production and DNA photocleavage was enhanced by increasing the rate of intersystem crossing between the photosensitizer’s singlet and triplet excited states [46]. A methylene violet-conjugated perylene diimide (near IR) was found to be a promising antitumor nanoagent through a photothermal/photodynamic combination mechanism [48]. Finally, β-carbolinebisindole compounds [49], chlorinated hexahydroquinolines [50], triazolylnucleosides [51] and bis-pyrimidine derivatives [52] when photosensitized under UV-A irradiation promote the photocleavage of DNA, with nucleosides and pyrimidines exhibiting antimicrobial activity as well.

Our team has recently shown a strong interest in the synthesis and biological evaluation of quinazoline and sulfonyloxy carbamidoxime organic ligands and their metal complexes [53,54,55], as well as in the DNA photocleavage of organic photosensitizers. Some of the derivatives were found to exhibit insecticidal activity on a major crop pest, the Whitefly *Bemisia tabaci*, under UV-A irradiation [56]. The so-called “privileged structures” that correspond to the quinazolinone ring system were part of our target scaffolds for the investigation of photoreactivity. Among the quinazolinones tested, the simple 6-nitro derivative (Figure 2, **I**) was found to photodegrade human melanoma cell lines under UV-A irradiation at a concentration 50 μM, whereas a 6-bromo analogue was found to photocleave DNA under UV-B irradiation [57]. In addition, several 3-amido-2-methyl-6-nitro substituted quinazolinones exhibited excellent DNA photocleavage activity at concentrations 1 μM, which was better compared to their parent 3-amino-2-methyl-6-nitro-quinazolinone (Figure 2, **J**, **K**, respectively) [58]. Molecular docking studies on the 6-nitro derivative **J** were indicative of satisfactory binding to DNA with participation of the nitro group and this was correlated with the observed photoactivity.

Comparing structures **G** and **J**, one may suggest that the scaffold of **G**, lacking the carbon joint between the two nitrogen atoms, literally represents the open and flexible form of **J**. We have therefore decided to synthesize a number of AA DACHZ bridged derivatives of **G** that bear electron-donating and electron-withdrawing groups (R) at a *p*- and *m*- position to the amine group and a variety of aroyl and heteroaroyl rings (R′) attached at the AA CO-NH-NH- bridge and test their DNA photocleavage activity under UV-A and UV-B irradiation. Furthermore, molecular docking studies were planned in order to observe similarities and differences between the open AA derivative (**G**) and the closed locked quinazolinone ring system (**J**) [58]. Finally, to assess the potential increase in cytotoxic effects, a series of toxicity and phototoxicity studies were conducted on melanoma cells with the derivatives that exhibited the best DNA photocleavage activity.

## 2. Results and Discussion

### 2.1. Chemistry

**Figure 3:**
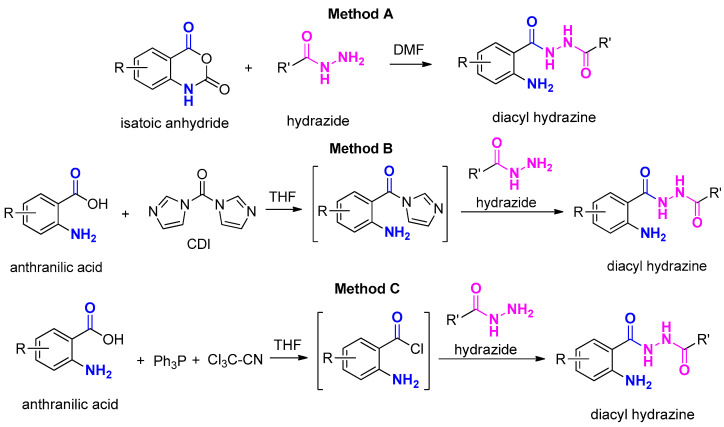
Methods for the synthesis of AA DACHz. Method A: Synthesis via isatoic anhydride; Method B: Synthesis via the in situ formation of anthraniloyl imidazole; Method C: Synthesis via the in situ formation of the anthraniloyl chloride.

There are several methods in the literature to form amide bonds using a carboxylic acid and an amine [59,60,61] which apply to the synthesis of hydrazides as well [33,62,63,64]. More specifically, the synthesis of anthranilamides is performed upon activation of the carboxylic acid with known classic reagents or with the use of isatoic anhydrides. In our case, depending of the availability of the reagents, we have used the nucleophilic attack of the proper hydrazide to (a) isatoic anhydride [20] (Figure 3, Method A), (b) the intermediate imidazole amides derived from AAs using *Ν*,*Ν’*-carbonyldiimidazole (CDI) as the coupling agent [65] and adopting a modified methodology for AAs [66] (Figure 3, Method B) and finally (c) the in situ formation of anthraniloyl chlorides upon treatment with Ph_3_P and CCl_3_CN anthraniloyl chlorides (Figure 3, Method C). The latter has been established for the synthesis of carboxylic acid amides, where triaryl phosphonium chloride is believed to be generated upon the reaction of Ph_3_P and CCl_3_CN. This reacts further with carboxylic acid to produce the corresponding acid chloride, triphenylphosphine oxide and dichloroacetonitrile [67].

**Figure 4:**
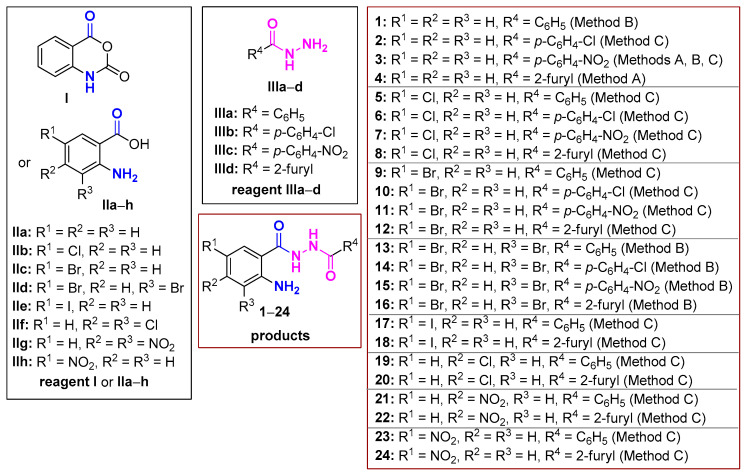
All synthesized AA DACHZs **1**–**24**. **1**–**4:** from the combination of **I** or **IIa** with all hydrazides **IIIa**–**d**; **5**–**8:** from the combination of **IIb** with all hydrazides **IIIa**–**d**; **9**–**12:** from the combination of **IIc** with all hydrazides **IIIa**–**d**; **13**–**16:** from the combination of **IId** with all hydrazides **IIIa**–**d**; **17**–**18:** from the combination of **IIe** with the hydrazides **IIIa**,**d**; **19**–**20:** from the combination of **IIf** with the hydrazides **IIIa**,**d**; **21**–**22:** from the combination of **IIg** with the hydrazides **IIIa**,**d**; **23**–**24:** from the combination of **IIh** with the hydrazides **IIIa**,**d**.

Our synthetic work is summarized in Figure 4. Τhe availability of isatoic anhydride has initially driven our synthetic attempts (Method A). According to this protocol, the combination of **I** and **IIIc**,**d** in DMF smoothly gave the derivatives **3** and **4**. However, the yields were generally moderate and, furthermore, in order to apply the same protocol for all derivatives we needed to synthesize the corresponding anhydrides. Thus, we turned our efforts towards the CDI-assisted coupling reaction (Method B). This procedure was applied for the reactions between the AAs **IIa**,**d** and **IIIa**–**d** in THF and the compounds **1**, **3** and **13**–**16**, where obtained. Products derived from the simple AA **ΙIa** needed column chromatography purification, but the 3,5-dibromo analogue **IId** derived products were insoluble in the reaction mixture and obtained in good yields upon simple filtration. Nevertheless, the reaction required an overnight stirring at room temperature and then heating for 1 h at 55 °C, then the addition of water and heating for another 1 h at 55 °C.

An alternative approach (Method C), involving the in situ generation of acid chlorides from the corresponding carboxylic acid followed by hydrazide attack furnished in several cases the desired products. This approach was adopted and adjusted to AAs, predicting the highest reactivity of hydrazide NH_2_ in comparison to aniline NH_2_ group present in the AAs. In general, the formation of the anthraniloyl chlorides in THF was fast (~1.5 h), whereas the rate of the second step depended on the substituents of starting materials **II** (1 to 12 h). Upon completion of the reaction, the mixture was extracted with water and ethyl acetate. The residue obtained after removal of the organic solvent was simply triturated with CH_2_Cl_2_ to furnish a precipitate which corresponded to the pure product. A small quantity of product remained in the aliquot, which was not further purified. The easiness of this synthetic protocol, the availability of the inexpensive starting materials and the facile work up to obtain pure products rendered this method as the best green choice to prepare most of the targeted derivatives, avoiding column chromatography and use of organic solvents.

Apart from the compounds **1**–**4**, the rest are novel. All compounds were fully characterized with ^1^H-NMR, ^13^C-NMR, IR and HRMS, and all data are in accordance with the proposed structure (Section 3 and Supplementary Materials S.1). The two NH moieties appear as two broad singlet peaks between 10.11 and 10.88 ppm except for the derivatives **13** and **21** where both hydrogens give a broad singlet integrated for two protons. The NH_2_ group appears as a broad singlet from 6.42 to 6.87 ppm for all derivatives **1**–**22**. The two 5-nitro-substituted compounds **23**, **24** having this electron-withdrawing group in the *p*-position relative to the amine group appear downfield at ~7.70 ppm. In the IR spectra, the absorptions at the area above 3200 cm^−1^ are also characteristic for the NH and NH_2_ groups, and amide carbonyl groups are found to absorb between 1630 and 1690 cm^−1^.

### 2.2. DNA Photointeractions of AA DACHZs with Plasmid DNA

#### 2.2.1. DNA Photo-Cleavage Experiments at 312 nm (UV-B Irradiation)

**Figure 5:**
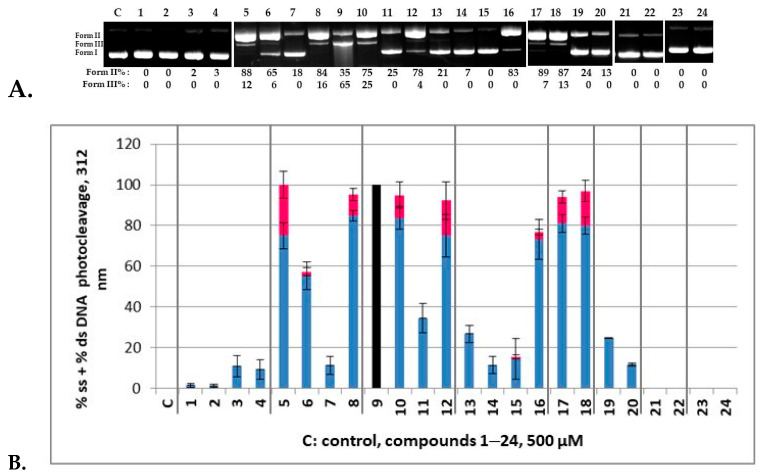
(**A**): A compilation of representative agarose gel pictures for the compounds **1**–**24** under 312 nm irradiation for 30 min, at a concentration of 500 μM. Calculations of Form II and Form III % are shown below the picture, compared to the control. Numbers 1–24 on the top correspond to the compounds **1**–**24** (all gel pictures are shown at Supplementary Materials S.2); (**B**): Plots of DNA photocleavage of the compounds **1**–**24** of all experiments: Error bars represent the standard deviation from at least two experiments; blue column: % ss photocleavage; red column: % ds photocleavage. The black column indicates that the concentration was too high to give measurable strands.

All compounds at a concentration of 500 μΜ were mixed with pBR322, incubated for 30 min and then irradiated for 30 min under UV-B irradiation at 312 nm. In Figure 5A, a compilation of representative agarose gel electrophoresis pictures for each compound (**1**–**24**) under UV-B irradiation is depicted. All experiments have been contacted at least twice. All runs are given in Supplementary Materials S.2: S.2.1–S.2.8 with calculations of Form II (nicked plasmid, % ss—single strand—damage) and Form III (linear plasmid, % ds—double strand—damage) photocleavage to be performed comparing to controls of irradiated plasmid DNA under the same conditions. On the top of the agarose gel, the numbers 1–24 one-to-one correspond to the compounds **1**–**24**, and **C** to the control irradiated plasmid DNA. In Figure 5B, plots of DNA photocleavage of all experiments is depicted. The % ss (Form II) is shown in blue and the % ds (Form III) is shown in red. Vertical gray lines divide the graph into groups of derivatives for easier reading. Thus, the compounds **1**–**4** resulted from the reactions of the AA **IIa**; **5**–**8** from 5-Cl-AA **IIb**; **9**–**12** from the 5-Br-AA **IIc**; and **13**–**16** from the 3,5-diBr-AA **IId**. Furthermore, each of those quartets have similar hydrazide residues, meaning Ph (from **IIIa**) for the first compound of the group; *p*-Cl-Ph (from **IIIb**) for the second; *p*-NO_2_-Ph (from **IIIc**) for the third; and finally the heteroaryl 2-furyl group (from **IIId**).

The compounds **1**–**4** were found to be almost inactive, regardless of the hydrazide residue (Supplementary Materials S.2.3 and S.2.7). In addition, the starting material **IIa** was also photochemically inactive (Supplementary Materials S.2.4). However, when we moved to the substituted AA **IIb** derivative (compounds **5**–**8**), we observed a significant DNA photocleavage that seems to be specifically higher for the Ph and furyl derivatives (Figure 5A,B, Lanes 5 and 8, respectively, Supplementary Materials S.2.2, S.2.3 and S.2.5) giving at this concentration both the nicked plasmid (Form II) and linear form (Form III). The substitution on the aryl groups seems to lower the activity. Interestingly, in the case of the nitroaryl group (Lane 7) this inactivity towards irradiation was particularly notable. Compared to the starting material **IIb** that exhibited a strong DNA photocleavage at 500 μM under UV-B irradiation, only the derivative **5** retained this activity, whereas **6**–**8** showed lower photoaction towards DNA (Supplementary Materials S.2.2–S.2.5).

Checking 5-Br-AA (**IIc**) and the related products **9**–**12**, we found that **IIc** results in high DNA photocleavage, comparable to its derivatives **10** and **12**; however, an extreme reactivity was observed for compound **9** (Figure 5A, Supplementary Materials S.2.2–S.2.4), where plasmid DNA nicely reacted photochemically with the photosensitizer to give both nicked and linear fragments at 35% and 65%, respectively (Supplementary Materials S.2.3). In other runs with the compound **9** (Supplementary Materials S.2.2 and S.2.4), plasmid DNA has totally vanished, indicating that a lower concentration is needed for this compound (*vide intra*). For this reason, in Figure 5B, where the average of all calculations of all experiments with their standard deviations is depicted, this plot is represented with a black color. The compound **10** showed a lower activity than **9**, and the nitro derivative **11** dramatically lost activity, whereas furyl heterocycle reestablished photointeraction. The introduction of a second bromine atom that inactivates the aromatic ring of the AA (**IId**) diminished the activity for all compounds of the group but the furyl one **16** that showed the same activity with the corresponding AA (Supplementary Materials S.2.1 and S.2.3).

The above-described set of experiments indicated that the phenyl and furyl hydrazide residues promote photosensitization. For this reason, further experiments that resulted from the combination of **IIIa**,**d** with the 5-I-AA (**IIe**), 4-Cl-AA (**IIf**), 4-NO_2_-AA (**IIg**) and 5-NO_2_-AA (**IIh**) were set up in order to examine the influence of a heavier halogen (I) or the position of the chlorine atom (5- or 4-). In addition, the 4-NO_2_ and 5-NO_2_ substituted AA could complete the study showing the effect of a highly electron-withdrawing group. Under UV-B irradiation, the I-derivatives **17** and **18** (Supplementary Materials S.2.4–S.2.6) had comparable activity with the 5-Cl analogues **5** and **8** and better than 5-I AA (**IIe**). Interestingly, derivatives bearing the Cl atom at the *meta* position relatively to NH_2_ (**19** and **20**) dropped all activity, as was also true for 4-Cl AA (**IIf**) (Supplementary Materials S.2.4–S.2.6). And even more surprising was the fact that all four nitro substituted compounds (**21**–**24**), as well as **IIg**,**h** regardless of the position of the nitro group, were found to exhibit zero activity (Supplementary Materials S.2.4, S.2.6 and S.2.8). Therefore, it seems that rigidity is the key for the high activity of nitro-quinazolinones [57] and their 3-amide substituted derivatives [58] that allows the nitro group to express its photochemistry in biological systems as it does in organic synthesis [68].

**Figure 6:**
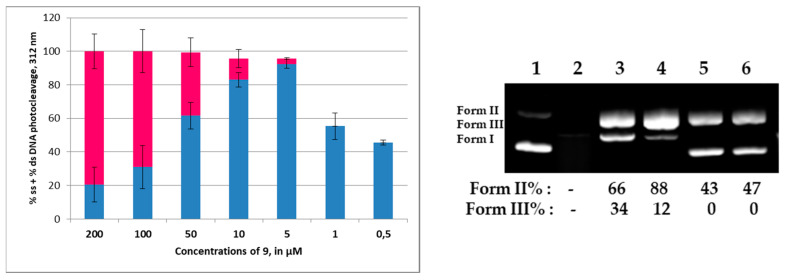
Diagram on the (**left**): Plots of DNA photocleavage of the compound **9** under various concentrations (indicated at horizontal axis): Error bars represent the standard deviation from at least two experiments; blue column: % ss cleavage; red column: % ds cleavage (for all gel pictures see Supplementary Materials S.3). Picture on the (**right**): DNA agarose gel picture (one experiment) for the compound **9** under 312 nm irradiation for 30 min, at concentrations of 100, 50, 10, 1, 0.5 μM, Lanes 2–6, respectively (Supplementary Materials S.3.2). Lane 1: control (DNA + UV). Calculations of Form II and Form III % are shown below the picture, compared to the control.

The compound **9** that exhibited the higher reactivity among all checked derivatives (**1**–**24**) has been subjected to concentration/reactivity relationship experiments in order to find an optimized concentration to perform pH influence experiments and mechanistic studies. One can observe in Figure 6 that the compound **9** exhibited 50% damage of the plasmid DNA at concentration as low as 1 μM (Supplementary Materials S.3.1 and S.3.2).

**Figure 7:**
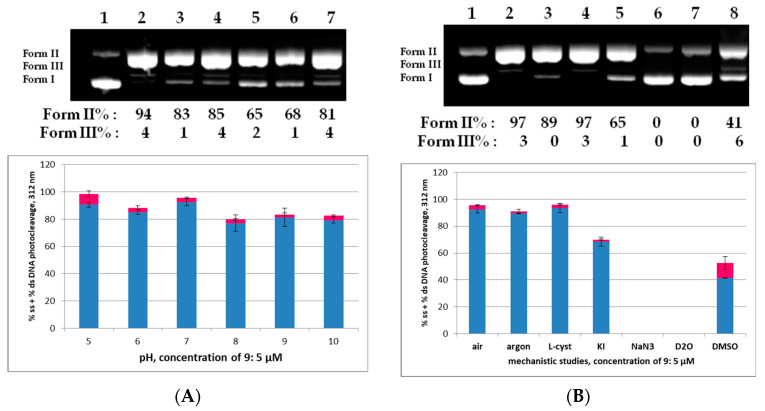
(**A**): (**Top**) picture: DNA agarose gel picture (one experiment) for the compound **9**, 312 nm, 30 min, at a concentration of 5 μM and pH 5–10. Lane 1: Control DNA at pH 7; Lanes 2–7: pH 5–10, respectively. Calculations of Form II and Form III % are shown below the picture, compared to the control. (**Bottom**) diagram: Plots of DNA photocleavage of the compound **9** of all experiments at pHs shown on the horizontal axis; Error bars represent the standard deviation from at least two experiments; blue column: % ss cleavage; red column: % ds cleavage, (Supplementary Materials S.3.3); (**B**): (**Top**) picture: DNA agarose gel picture (one experiment) for the compound **9**, 312 nm, 30 min, at concentration of 5 μM. Mechanistic studies: Lane 1: control, Lanes 2 and 3: under air and under argon; Lanes 4–8: under air and various scavengers like: L-cyst, KI, NaN_3_, D_2_O and DMSO, respectively. (**Bottom**) diagram: Plots of DNA photocleavage of the compound **9** of all mechanistic experiments shown on the horizontal axis. Blue and red columns as in (**A**) (Supplementary Materials S.3.4).

As far as the pH influence on the DNA photocleavage experiment concerns, it is obvious that for the compound **9** (5 μΜ) a pH above 8 had little effect on the photocleavage (Figure 7A, top picture, Lanes 2–7 for pH 5–10, respectively, and bottom diagram, Supplementary Materials S.3.3), whereas below 8 and up to pH 5 no change was observed. Moreover, mechanistic studies show that a lack of molecular oxygen does not affect the reactivity (Figure 7B, top picture, Lane 3 and bottom diagram, Supplementary Materials S.3.4), which probably means that the homolysis of the C-Br bond may occur [57]. In the presence of oxygen, a singlet oxygen scavenger like NaN_3_ indicated the formation of this species, whereas a hydroxyl radical scavenger like DMSO verified their presence (Figure 7B, top picture, Lanes 6 and 8, respectively, Supplementary Materials S.3.4). Therefore, the compound **9** seems to react under Type I and Type II photosensitized oxidation reactions [69].

#### 2.2.2. DNA Photo-Cleavage Experiments at 365 nm (UV-A Irradiation)

All compounds at a concentration of 500, 100, 10 and 2 μΜ were mixed with pBluescript SK II DNA, incubated for 30 min and then irradiated for 120 min under UV-A irradiation at 365 nm (broad band lamb). Concentrations below 500 μΜ were chosen after being apparent that the compounds were very active. The same applies for concentrations below 100 and 10 μΜ. All AA DACHZs absorb light in this area. The compounds 3-Arylamide-6-Br-2-Me-quinazolinones [58] that closely resemble open forms of **9**–**11** in the UV–Vis spectra did not absorb with a high ε, with values very far from 315 nm. All sets of derivatives gave a broad shoulder with a high ε from 300 to 400 nm in UV–Vis spectra (Supplementary Materials S.4), except **23** and **24** which showed a right shift and absorption from 350 to 500 nm. The compound **J** (Figure 2, Ar = Ph) which is the rigid analogue of **23** had a peak at 325 nm [58].

**Figure 8:**
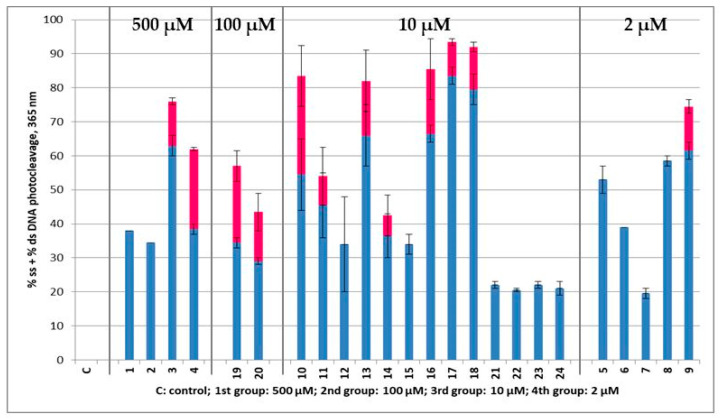
Plots of DNA photocleavage of the compounds **1**–**24** under various concentrations, at 365 nm. C: control; 1st group of four compounds separated with gray lines are compounds that gave DNA photocleavage at a 500 μΜ concentration; 2nd group—two compounds: 100 μΜ concentration; 3rd group—thirteen compounds: 10 μΜ concentration; 4th group—five compounds: 2 μΜ concentration. Error bars represent the standard deviation from at least two experiments; blue column: % ss cleavage; red column: % ds cleavage (all gel agarose pictures at Supplementary Materials S.5).

In Figure 8 (Supplementary Materials S.5 for the agarose gel pictures), it is shown that the compounds **1**–**4** that were inactive under UV-B irradiation at 500 μΜ exhibited some DNA photocleavage under UV-A light. This may be attributed to the higher UV–Vis absorption in this area. However, compared to the rest of the compounds, they still seemed reluctant to be excited. In addition, derivatives bearing the Cl atom in the *meta* position relative to NH_2_ (**19** and **20**) showed a lower activity (moderate action at concentration 100 μΜ) compared to their corresponding *p*-analogues **5** and **8**, which were active at concentrations as low as 2 μΜ. The activity among the AA DACHZs of this group (**5**–**8**) indicated again that the nitroaryl moiety lowered DNA photocleavage (derivative **7**).

What was left to be analyzed was the group of the 5-Br-AA DACHZs (**9**–**12**), of the 3,5-diBr-AA DACHZs (**13**–**16**) and of the 5-I-AA DACHZs (**17**, **18**). In addition to the compound **9** (2 μΜ), the rest of the derivatives **10**–**18** were active at a concentration of 10 μΜ. Both nitrophenyl hydrazides’ (**IIIc**) products **11** and **15** had reduced activity in relation to other members of their group. The rest of the compounds were sufficiently active at a 10 μM concentration with the 5-I-AA DACHZs (**17**, **18**) being the most potent ones. The next group with low activity (10 μM) was the nitro-AA derivatives **21**–**24**. Again, the nitro derivatives seemed to be very inactive.

The much higher activity of the compounds **5**–**8** and **10**–**20** that photocleaved DNA at much lower concentrations under UV-A irradiation compared to UV-B might be attributed to the very high absorption between 300 and 400 nm. However, this does not seem to explain the reactivity of the derivative **9** that keeps the higher activity in both UV-B and UV-A irradiation at similar concentrations. Certainly, since none of the compounds was tested for photosensitivity by itself but as a DNA complex, the phenomenon is much more complex. Nonetheless, the bromine atom in this position may contribute to a high atom effect in the photochemistry of the compound [42,46,47].

### 2.3. Molecular Docking “In Silico” Calculations of DNA/AA DACHZs

Molecular docking studies for the derivatives **1**–**24** were conducted using the AutoDock Vina program. The objective was to identify polar contacts and calculate the energy of their DNA binding. All calculated energy binding values, along with DNA base interactions, are given in Table 1. To gain a deeper understanding of the conformational preferences of the compounds within DNA, a 3D calculation program (PyMOL) was employed to identify all polar contacts with both DNA strands.

Generally, one may observe a network of polar contacts and hydrogen bonds arising from the free -NH_2_ group and the carbonyl groups in all derivatives. Upon closer examination of the docking results for each group, the first group (compounds **1**, **2**, **3** and **4**) exhibits good binding with DNA. However, when irradiated in the UV-B area, all compounds were inactive. In the UV-A area, only the compounds **3** and **4** demonstrated significant DNA photocleavage, resulting in both the nicked plasmid (Form II) and linear form (Form III), although this was at the highest concentration (500 μM). In the second group (compounds **5**, **6**, **7** and **8**), a stronger binding to DNA was observed in all 5-Cl derivatives, as they exhibited a higher binding energy compared to the compounds in the previous group. The compounds **5** and **8** showed a higher photocleavage effect after irradiation in the UV-B area, and these same compounds proved to be active even at small concentrations (2 μM) when irradiation occurred in the UV-A area.

Moving on to the third group of compounds (**9**, **10**, **11** and **12**), it is noteworthy that, excluding the compound **12**, the 5-Br derivatives **10** and **11** exhibited a UV-A photocleavage effect (10 μM) at much lower concentrations than UV-B. the compound **9** demonstrated exceptional results under UV-B irradiation, despite having the lowest binding affinity with DNA among all the other compounds in this group. Under UV-A light, the compound **9** remained active even at a concentration of 2 μM.

In the fourth group, all the 4,5-bis-bromo derivatives demonstrated a good binding affinity, with the compound **15** having a binding energy value of −10.1 kcal/mol. However, despite exhibiting the highest binding energy among all the compounds, this one does not appear to be significantly active in either the UV-A or UV-B area. The other three compounds exhibited remarkable photocleavage results under UV-A irradiation at a concentration of 10 μM. Another characteristic of these compounds is the formation of polar contacts solely with the –NH_2_ group. The only exception to this is the compound **16**, which forms hydrogen bonds with both the carbonyl group and the oxygen from the furyl group.

Regarding the 5-I derivatives (**19**, **20**), one may observe the formation of polar contacts through both the –NH_2_ group and the –C=O group in both cases. Moreover, these compounds are particularly active in the UV-A area of irradiation. Plasmid DNA reacted photochemically with the photosensitizers at a concentration of 10 μM, yielding both nicked and linear fragments.

In the case of 4-Cl derivatives (**21**, **22**), although the binding energy remained at a good level, their activity decreased significantly under UVB irradiation, while in the UVA area, their activity increased. This can be partially attributed to the significant absorption of the compounds in the UV-A region.

**Figure 9:**
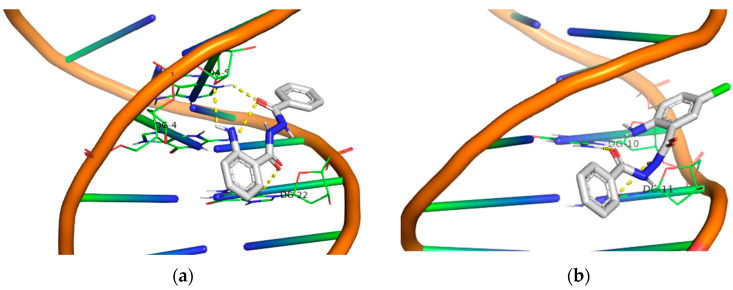
3D structures of the polar contacts of selected compounds of each group. (**a**) Compound **1**; (**b**) Compound **5**.

**Figure 10:**
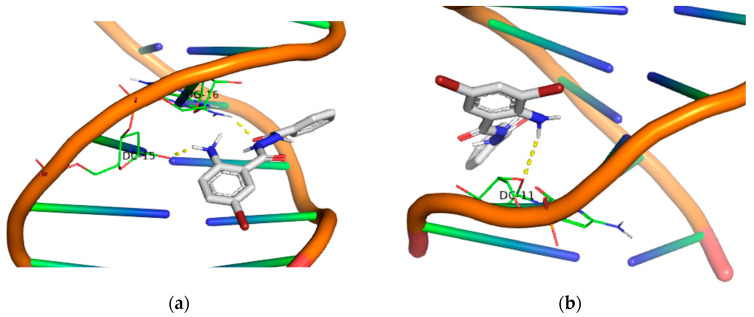
3D structures of the polar contacts of selected compounds of each group. (**a**) Compound **9**; (**b**) Compound **13**.

**Figure 11:**
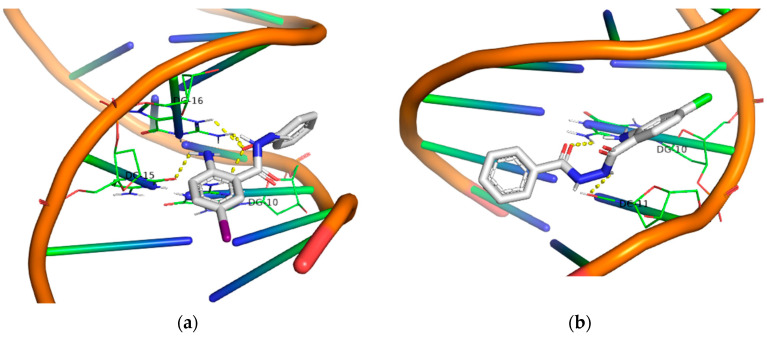
3D structures of the polar contacts of selected compounds of each group. (**a**) Compound **17**; (**b**) Compound **19**.

**Figure 12:**
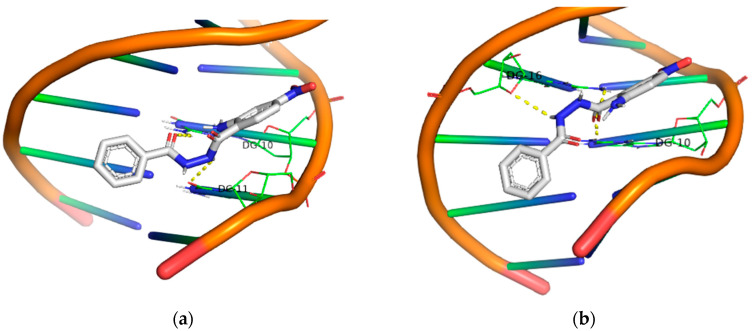
3D structures of the polar contacts of selected compounds of each group. (**a**) Compound **21**; (**b**) Compound **23**.

Finally, all the –NO_2_ derivatives (**21**–**24**) utilized the -NH_2_ and –C=O groups to form hydrogen bonds with the DNA bases. Even though the -NO_2_ group does not participate in this network of polar contacts (with the compound **24** being the only exception), the binding of these compounds to DNA is significantly strong. Although all nitro derivatives proved to be ineffective under UV-B light, in the UV-A area they demonstrate a moderate photocleavage ability, fragmenting DNA at a concentration of 100 μM. In Figure 9, Figure 10, Figure 11 and Figure 12 images of all phenyl substituted AA DACHZ compounds **1**, **5**, **9**, **13**, **17**, **19**, **21** and **23** are shown (Supplementary Materials S.7). 

**Figure 13:**
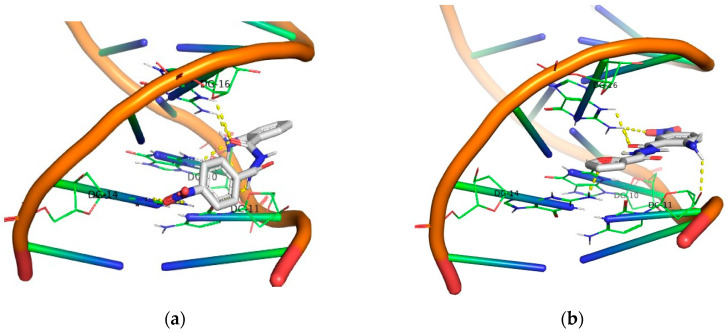
3D structures of the polar contacts of selected compounds of each group. (**a**) Compound **3**; (**b**) Compound **24**.

The comparison of open and closed structures is also considered useful as it can lead to conclusions. In the closed structures [58], we observed the crucial role of the –NO_2_ group in strengthening the binding energy, as this group formed polar contacts with the DNA bases. On the contrary, in open structures, the –NO_2_ group does not appear to play a significant role. Specifically, among the four –NO_2_ derivatives (**21**–**24**), only the compound **24** showed the participation of the –NO_2_ group in interaction with DNA. Furthermore, among the four compounds bearing the –NO_2_ group in the ring of hydrazide (**3**, **7**, **11**, **15**), again only one (**3**) out of four compounds exhibited participation of the -NO_2_ group in polar contacts (Figure 13, Supplementary Materials S.6).

When considering binding energy, it is noteworthy that, despite open structures having more available groups for hydrogen bond formation, the binding in closed structures is slightly stronger than in the open ones, respectively.

### 2.4. Cell Culture Experiments of Selective AA DACHZs with Melanoma Cell Lines

**Figure 14:**
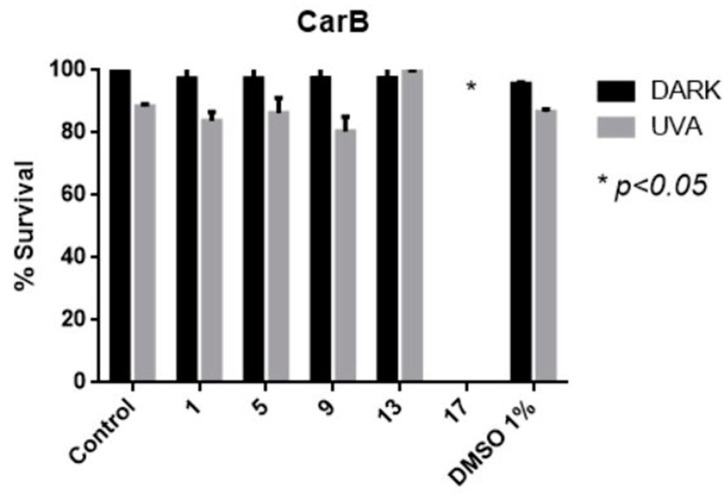
The cytotoxic effect of the compounds **1**, **5**, **9**, **13** and **17** on the CarB cell line. The compound **17** exhibits a high cytotoxic effect.

The highly malignant melanoma cell line CarB were used for cell culture experiments. These cells derive from squamous cell carcinoma of the mouse skin. Generally, skin cancer is preferred in drug photoactivation studies due to the penetration of UV radiation [70]. Moreover, our aim was to test the compounds activity on highly aggressive and metastatic cells. The cells were incubated with 100 μM of each selected compound **1**, **5**, **9**, **13** and **17** and were irradiated at 365 nm (broad band) for 1 h. The selection was based on DNA photocleavage experiments where all compounds **5**, **9** and **13**, **17** were found active at 2 and 10 μM, respectively (Figure 8, Supplementary Materials S.5). Derivative **1** was selected for comparison, since it bears the same (phenyl) group attached on the diacylhydrazine moiety. It was observed that **17** possesses a high cytotoxic role; however, this role is not specific after UV irradiation. Among the compounds **1**, **5**, **9** and **13**, it seems that **9** has a slight phototoxicity; nevertheless, this toxicity is not that impressive at 100 μM (Figure 14).

## 3. Materials and Methods

All commercially available reagent-grade chemicals and solvents were used without further purification. pB322 supercoiled plasmid was purchased from New England Biolabs (Ipswich, MA, USA). pBluescript SK II was laboratory produced. UV–Visible (UV–Vis) spectra were recorded on a Hitachi U–2001 dual beam UV–Vis spectrophotometer (Hitachi, Tokyo, Japan). NMR spectra were recorded on an Agilent 500/54 (Agilent Technologies, Santa Clara, CA, USA) (500 MHz and 125 MHz for ^1^H and ^13^C, respectively) or on a Bruker 300 AM (Bruker, Billerica, MA, USA) (300 MHz and 75 MHz for ^1^H and ^13^C, respectively) spectrometer using DMSO-*d*_6_ as a solvent. *J* values are reported in Hz. High-resolution mass spectra measured with an LTQ ORBITRAP XL with an ETD-Thermo Fisher Scientific Ion Source (Thermo Scientific, Waltham, MA, USA): Electrospray Ionization (ESI) positive mode Mass Analyser: Orbitrap. All samples containing pBR322 or pBluescript SK II plasmid were irradiated at pH 6.8 with Philips 2 × 9 W/01/2P UV−B narrowband lamps (Amsterdam, The Netherlands) at 312 nm and Philips 2 × 9 W/10/2P UV-A broad band lamps at 365 nm. All reactions were monitored on commercially available pre-coated TLC plates (layer thickness 0.25 mm) of Kieselgel 60 F_254_ (Merck, Darmstadt, Germany). Melting points were measured on GallenKamp MFB-595 melting point apparatuses (GallenKamp, Cambridge, UK) and are uncorrected. The calculation of yields was based on the amount of the crystallized product collected.

### 3.1. Synthesis of AA DACHZs **1**–**24**

Method A: Isatoic anhydride (**I**) (2 mmol), the corresponding hydrazide (**IIIc** or **IIId**) (2 mmol) and Et_3_N (2 mmol) were added in DMF (1.2 mL) and the mixture was stirred at r.t. for 18 h. Iced water was added; the precipitate was filtrated and recrystallized from the proper solvent.

Method B: Modified from ref [66]. A mixture of the corresponding anthranilic acid **IIa** or **IId** (2 mmol) and CDI (2 mmol) in 5 mL THF (dry, commercially available) was stirred at 0 °C for 1 h and then for 2 h at r.t. Hydrazide (**IIIa**–**d**) was added diluted in 5 mL THF and the mixture was stirred overnight at r.t. The temperature was raised to 55 °C and the mixture was heated for 1 h. After this period, water was added (2 mL) and the mixture was heated for 1 h more, at the same temperature. After concentration of the solvents under reduced pressure, 0.1 M NaOH (30 mL) was added, and the residue was extracted with EA (3 × 30 mL). The organic layers were separated, dried with Na_2_SO_4_, and removed under reduced pressure. In the case of the compounds **1** and **3**, trituration with CH_2_Cl_2_ gave a precipitate which was filtrated, purified by column chromatography and recrystallized from the proper solvent to give the pure compound. For **IId** and all derivatives **13**–**16**, no extraction was necessary. Directly after completion of the reaction, filtration of the precipitate gave the crude product which was purified by recrystallization.

Method C: Modified from ref [67]. A total of 4 mmol of Ph_3_P and 2 mmol of the corresponding anthranilic acid **IIa**–**c** or **IIe**–**h** were individually mixed, added in toluene and dried with the azeotropic removal of any moisture with the solvent under reduced pressure. The solid powder was then added in 20 mL dry THF (commercially available) under argon. Cl_3_C–CN (5 mmol) was poured in the flask and the mixture was stirred at r.t. for 1.5 h. After being dried with azeotropic removal of any moisture with toluene under reduced pressure, the corresponding hydrazide **IIIa**–**d** (2 mmol) was added into the mixture with a subsequent addition of dry Et_3_N (6 mmol). The mixture was stirred from 1 to 12 h and then H_2_O 50 mL was added, and the mixture was extracted with EA (3 × 50 mL). The organic layers were dried with Na_2_SO_4_ and were removed under reduced pressure. The addition of CH_2_Cl_2_ gave a precipitate which was filtered and the obtained solid was recrystallized from the proper solvent.

*2-Amino-N*′*-benzoylbenzohydrazide* (**1**): Method B; white amorphous solid; mp: 180–182 °C (EA/hex), lit: 210–212 °C [20]; yield: 47%; IR (KBr) cm^−1^: 3408, 3272, 1674, 1645, 1614; ^1^H-NMR (DMSO-*d*_6_, 500 MHz) *δ* 10.38 (bs, 1H, NH), 10.17 (bs, 1H, NH), 7.93 (brs, 2H), 7.61 (bs, 2H), 7.52 (s, 2H), 7.20 (s, 1H), 6.75 (s, 1H), 6.55 (s, 1H), 6.43 (bs, 2H, NH_2_) ppm; ^13^C-NMR (DMSO-*d*_6_, 125 MHz) *δ* 168.3, 166.0, 149.9, 132.7, 132.3, 131.8, 128.5, 128.2, 127.5, 116.5, 114.7, 112.56 ppm; HRMS(ESI) *m*/*z* [M+H]^+^: C_14_H_14_N_3_O_2_^+^, calc: 256.1081; found: 256.1080.
*2-Amino-N*′*-(4-chlorobenzoyl)benzohydrazide* (**2**): Method C (1.5 h + 1 h); off-white amorphous solid; mp: 226.4 °C (EA/hex), lit: 238–240 °C [14]; yield: 52%; IR (KBr) cm^−1^: 3414, 3312, 3255, 1678, 1647, 1612; ^1^H-NMR (DMSO-*d*_6_, 500 MHz) *δ* 10.47 (s, 1H, NH), 10.19 (brs, 1H, NH), 7.94 (d, *J* = 8.3 Hz, 2H), 7.60 (d, *J* = 8.3 Hz, 2H), 7.20 (t, *J* = 7.5 Hz, 1H), 6.74 (d, *J* = 8.1 Hz, 1H), 6.55 (t, *J* = 7.4 Hz, 1H), 6.43 (brs, 2H, NH_2_) ppm; ^13^C-NMR (DMSO-*d*_6_, 125 MHz) *δ* 168.3, 165.0, 150.0, 136.7, 132.4, 131.4, 129.4, 128.6, 128.2, 116.4, 114.6, 112.4 ppm; HRMS(ESI) *m*/*z* [M+H]^+^: C_14_H_13_ClN_3_O_2_^+^, calc: 290.0691; found: 290.0689, 290.0660 (3/1).
*2-Amino-N*′*-(4-nitrobenzoyl)benzohydrazide* (**3**): Method A, B, C; yellow amorphous solid; mp: 239.0 °C (EA/EtOH), lit: 238–240 °C [12], 238 °C [71]; yield: 42%, 52%, 47%, respectively for each method used; IR (KBr) cm^−1^: 3407, 3310, 3301, 3244, 1682, 1642, 1613; ^1^H-NMR (DMSO-*d*_6_, 500 MHz) *δ* 10.73 (s, 1H, NH), 10.29 (brs, 1H, NH), 8.37 (d, *J* = 8.8 Hz, 2H), 8.15 (d, *J* = 8.7 Hz, 2H), 7.62 (d, *J* = 7.1 Hz, 1H), 7.20 (dt, *J* = 8.3, 1.3 Hz, 1H), 6.75 (d, *J* = 7.7 Hz, 1H), 6.56 (t, *J* = 7.2 Hz, 1H), 6.45 (brs, 2H, NH_2_) ppm; ^13^C-NMR (DMSO-*d*_6_, 125 MHz) *δ* 168.2, 164.5, 150.0, 149.4, 138.3, 132.5, 129.0, 128.2, 123.8, 116.5, 114.6, 112.1 ppm; HRMS(ESI) *m*/*z* [M+H]^+^: C_14_H_13_N_4_O_4_^+^, calc: 301.0931; found: 301.0933.
*N*′*-(2-Aminobenzoyl)furan-2-carbohydrazide* (**4**): Method A; beige amorphous solid; mp: 194.2–195 °C (EA/EtOH); lit: 285–287 °C [20]; yield: 44%; IR (KBr) cm^−1^: 3414, 3317, 3280, 1680, 1645, 1614; ^1^H-NMR (DMSO-*d*_6_, 500 MHz) *δ* 10.25 (s, 1H, NH), 10.11 (brs, 1H, NH), 7.91 (d, *J* = 0.9 Hz, 1H), 7.58 (dd, *J* = 7.9, 0.9 Hz, 1H), 7.25 (d, *J* = 3.4 Hz, 1H), 7.19 (dt, *J* = 7.0, 1.3 Hz, 1H), 6.74 (dd, *J* = 8.1, 0.7 Hz, 1H), 6.67 (dd, *J* = 3.4, 1.7 Hz, 1H), 6.54 (dt, *J* = 7.8, 0.9 Hz, 1H), 6.42 (brs, 2H, NH_2_) ppm; ^13^C-NMR (DMSO-*d*_6_, 125 MHz) *δ* 168.3, 157.5, 150.0, 146.4, 145.7, 132.4, 128.2, 116.4, 114.6, 114.5, 112.3, 111.9 ppm; HRMS(ESI) *m*/*z* [M+H]^+^: C_12_H_12_N_3_O_3_^+^, calc: 246.0873; found: 246.0874.
*2-Amino-N*′*-benzoyl-5-chlorobenzohydrazide* (**5**): Method C (1.5 h + 2 h); off-white amorphous solid; mp: 203.1 °C (EA/EtOH); yield: 52%; IR (KBr) cm^−1^: 3486, 3372, 3310, 3280, 1684, 1642, 1612; ^1^H-NMR (DMSO-*d*_6_, 500 MHz) *δ* 10.47 (s, 1H, NH), 10.34 (brs, 1H, NH), 7.92 (d, *J* = 7.5 Hz, 2H), 7.67 (brs, 1H), 7.60 (t, *J* = 7.3 Hz, 1H), 7.52 (t, *J* = 7.6 Hz, 2H), 7.24 (dd, *J* = 8.8, 1.8 Hz, 1H), 6.78 (d, *J* = 8.8 Hz, 1H), 6.60 (brs, 2H, NH_2_) ppm; ^13^C-NMR (DMSO-*d*_6_, 125 MHz) *δ* 167.3, 166.0, 148.9, 132.5, 132.2, 132.0, 128.6, 127.5, 127.5, 118.2, 117.8, 113.2 ppm; HRMS(ESI) *m*/*z* [M+H]^+^: C_14_H_13_ClN_3_O_2_^+^, calc: 290.0691; found: 290.0692, 292.0661 (3/1).
*2-Amino-5-chloro-N*′*-(4-chlorobenzoyl)benzohydrazide* (**6**): Method C (1.5 h + 2.5 h); off white amorphous solid; mp: 233.2 °C (EA/EtOH); yield: 41%; IR (KBr) cm^−1^: 3482, 3347, 3222, 3164, 1663, 1636, 1595; ^1^H-NMR (DMSO-*d*_6_, 500 MHz) *δ* 10.55 (s, 1H, NH), 10.36 (brs, 1H, NH), 7.93 (d, *J* = 8.2 Hz, 2H), 7.66 (s, 1H), 7.61 (d, *J* = 8.1 Hz, 2H), 7.24 (d, *J* = 8.0 Hz, 1H), 6.77 (d, *J* = 8.8 Hz, 1H), 6.60 (brs, 2H, NH_2_) ppm; ^13^C-NMR (DMSO-*d*_6_, 125 MHz) *δ* 167.2, 165.0, 149.0, 136.8, 132.3, 131.3, 129.5, 128.8, 127.5, 118.3, 117.8, 113.0 ppm; HRMS(ESI) *m*/*z* [M+H]^+^: C_14_H_12_Cl_2_N_3_O_2_^+^, calc: 324.0301; found: 324.0302, 326.0273, 328.0244, M, M+2, M+4 (9/6/1).
*2-Amino-5-chloro-N*′*-(4-nitrobenzoyl)benzohydrazide* (**7**): Method C (1.5 h + 1 h); light yellow amorphous solid; mp: 235.4 °C (EA/hex); yield: 48%; IR (KBr) cm^−1^: 3503, 3370, 3219, 3047, 1666, 1637, 1600; ^1^H-NMR (DMSO-*d*_6_, 500 MHz) *δ* 10.77 (brs, 1H, NH), 10.44 (brs, 1H, NH), 8.37 (d, *J* = 8.1 Hz, 2H), 8.14 (d, *J* = 8.1 Hz, 2H), 7.66 (s, 1H), 7.24 (d, *J* = 7.3 Hz, 1H), 6.78 (d, *J* = 8.6 Hz, 1H), 6.58 (brs, 2H, NH_2_) ppm; ^13^C-NMR (DMSO-*d*_6_, 125 MHz) *δ* 167.1, 164.5, 149.5, 148.9, 138.2, 132.3, 129.0, 127.4, 123.8, 118.3, 117.8, 112.9 ppm; HRMS(ESI) *m/z* [M+H]^+^: C_14_H_12_ClN_4_O_4_^+^, calc: 335.0542; found: 335.0541, 337.0511 (3/1).
*N*′*-(2-Amino-5-chlorobenzoyl)furan-2-carbohydrazide* (**8**): method C (1.5 h + 1 h); beige amorphous solid; mp: 232.3 °C (EA/hex); yield: 49%; IR (KBr) cm^−1^: 3465, 3435, 3206, 3017, 1618, 1584; ^1^H-NMR (DMSO−*d_6_*, 500 MHz) *δ* 10.34 (s, 1H, NH), 10.28 (brs, 1H, NH), 7.92 (s, 1H), 7.64 (s, 1H), 7.26 (d, *J* = 2.8 Hz, 1H), 7.24 (d, *J* = 9.0 Hz, 1H), 6.77 (d, *J* = 8.9 Hz, 1H), 6.68 (s, 1H), 6.59 (brs, 2H, NH_2_) ppm; ^13^C-NMR (DMSO-*d*_6_, 125 MHz) *δ* 167.2, 157.5, 149.0, 146.3, 145.9, 132.3, 127.4, 118.3, 117.8, 114.7, 113.0, 112.0 ppm; HRMS(ESI) *m*/*z* [M+H]^+^: C_12_H_11_ClN_3_O_3_^+^, calc: 280.0483; found: 280.0481, 282.0451 (3/1).
*2-Amino-N*′*-benzoyl-5-bromobenzohydrazide* (**9**): Method C (1.5 h + 2 h); off-white amorphous solid; mp: 207.0 °C (1,4-dioxane/EtOH); yield: 45%; IR (KBr) cm^−1^: 3411, 3282, 1673, 1644, 1604; ^1^H-NMR (DMSO-*d*_6_, 500 MHz) *δ* 10.43 (s, 1H, NH), 10.31 (brs, 1H, NH), 7.91 (d, *J* = 7.5Hz, 2H), 7.78 (s, 1H), 7.60 (t, *J* = 7.6 Hz, 1H), 7.52 (t, *J* = 7.6 Hz, 2H), 7.33 (d, *J* = 8.9 Hz, 1H), 6.73 (d, *J* = 8.9 Hz, 1H), 6.59 (brs, 2H, NH_2_) ppm; ^13^C-NMR (DMSO-*d*_6_, 125 MHz) *δ* 167.1, 165.9, 149.1, 134.8, 132.5, 131.9, 130.3, 128.5, 127.5, 118.6, 113.9, 104.9 ppm; HRMS(ESI) *m*/*z* [M+H]^+^: C_14_H_13_BrN_3_O_2_^+^, calc: 334.0186; found: 334.0183, 336.0163 (1/1).
*2-Amino-5-bromo-N*′*-(4-chlorobenzoyl)benzohydrazide* (**10**): Method C (1.5 h + 2 h); off-white amorphous solid; mp: 205–207 °C (EA/EtOH); yield: 56%; IR (KBr) cm^−1^: 3376, 3276, 1679, 1645, 1593; ^1^H-NMR (DMSO-*d*_6_, 500 MHz) *δ* 10.54 (brs, 1H, NH), 10.35 (brs, 1H, NH), 7.93 (d, *J* = 8.5 Hz, 2H), 7.77 (d, *J* = 1.9 Hz, 1H), 7.60 (d, *J* = 8.5 Hz, 2H), 7.34 (dd, *J* = 8.8, 2.0 Hz, 1H), 6.73 (d, *J* = 9 Hz, 1H), 6.60 (brs, 2H, NH_2_) ppm; ^13^C-NMR (DMSO-*d*_6_, 125 MHz) *δ* 167.1, 164.9, 149.2, 136.8, 134.9, 131.2, 130.3, 129.4, 128.7, 118.6, 113.8, 104.9 ppm; HRMS(ESI) *m*/*z* [M+H]^+^: C_14_H_12_BrClN_3_O_2_^+^, calc: 367.9796; found: 367.9793, 369.9770, 371.9838 M, M+2, M+4 (3/4/2).
*2-Amino-5-bromo-N*′*-(4-nitrobenzoyl)benzohydrazide* (**11**): Method C (1.5 h + 2 h); beige amorphous solid; mp: 221.5 °C (EA/hex); yield: 79%; IR (KBr) cm^−1^: 3387, 3290, 1682, 1646, 1605; ^1^H-NMR (DMSO-*d*_6_, 500 MHz) *δ* 10.78 (s, 1H, NH), 10.44 (brs, 1H, NH), 8.37 (d, *J* = 8.5 Hz, 2H), 8.14 (d, *J* = 8.5 Hz, 2H), 7.79 (s, 1H), 7.35 (d, *J* = 8.9 Hz, 1H), 6.74 (d, *J* = 8.9 Hz, 1H), 6.61 (brs, 2H, NH_2_) ppm; ^13^C-NMR (DMSO-*d*_6_, 125 MHz) *δ* 167.0, 164.4, 149.4, 149.2, 138.1, 134.9, 130.3, 129.0, 123.8, 118.6, 113.5, 104.9 ppm; HRMS(ESI) *m*/*z* [M+H]^+^: C_14_H_12_BrN_4_O_4_^+^, calc: 379,0036; found: 379.0037, 381.0017 (1/1).
*N*′*-(2-Amino-5-bromobenzoyl)furan-2-carbohydrazide* (**12**): Method C (1.5 h + 2 h); beige amorphous solid; mp: 212.4–217.7 °C (EA/EtOH); yield: 50%; IR (KBr) cm^−1^: 3466, 3360, 3213, 1664, 1614, 1581; ^1^H-NMR (DMSO-*d*_6_, 500 MHz) *δ* 10.32 (s, 1H, NH), 10.27 (brs, 1H, NH), 7.92 (d, J = 0.8 Hz, 1H), 7.75 (d, *J* = 2.1 Hz, 1H), 7.33 (dd, *J* = 8.9, 2.1 Hz, 1H), 7.26 (d, *J* = 3.3 Hz, 1H), 6.72 (d, J = 8.9 Hz, 1H), 6.68 (dd, J = 3.3, 1.6 Hz, 1H), 6.59 (brs, 2H, NH_2_) ppm; ^13^C-NMR (DMSO-*d*_6_, 125 MHz) *δ* 167.1, 157.5, 149.2, 146.3, 145.8, 134.9, 130.3, 118.6, 114.6, 113.7, 111.9, 104. ppm; HRMS(ESI) *m*/*z* [M+H]^+^: C_12_H_11_BrN_3_O_3_^+^, calc: 323.9978; found: 323.9978, 325.9957 (1/1).
*2-Amino-N*′*-benzoyl-3,5-dibromobenzohydrazide* (**13**): Method B; white amorphous solid; mp: 275.9 °C (EtOH); yield: 46%; IR (KBr) cm^−1^: 3464, 3340, 3219, 1668, 1632, 1602; ^1^H-NMR (DMSO-*d*_6_, 500 MHz) *δ* 10.54 (brs, 2H, NH, NH), 7.91 (d, *J* = 7.3 Hz, 2H), 7.81 (s, 2H), 7.59 (t, *J* = 7.1 Hz, 1H), 7.52 (t, *J* = 7.4 Hz, 2H), 6.58 (brs, 2H, NH_2_) ppm; ^13^C-NMR (DMSO-*d*_6_, 125 MHz) *δ* 166.5, 165.9, 145.5, 137.0, 132.3, 132.0, 130.2, 128.6, 127.5, 116.0, 110.3, 105.2 ppm; HRMS(ESI) *m*/*z* [M+H]^+^: C_14_H_12_Br_2_N_3_O_2_^+^, calc: 411,9291; found: 411.9290, 413.9269, 415.9250 (1/2/1).
*2-Amino-3,5-dibromo-N*′*-(4-chlorobenzoyl)benzohydrazide* (**14**): Method B; light yellow amorphous solid; mp: 282.9 °C (1,4-dioxane); yield: 73%; IR (KBr) cm^−1^: 3483, 3342, 3031, 1658, 1630, 1601; ^1^H-NMR (DMSO-*d*_6_, 500 MHz) *δ* 10.64 (brs, 1H, NH), 10.57 (brs, 1H, NH), 7.81 και 7.94 (two doublets overlapped, 4H), 7.62 (s, 1H), 6.58 (brs, 2H, NH_2_) ppm; ^13^C-NMR (DMSO-*d*_6_, 125 MHz) *δ* 166.5, 164.9, 145.5, 137.1, 136.9, 131.0, 130.1, 129.4, 128.7, 115.9, 110.3, 105.2 ppm; HRMS(ESI) *m*/*z* [M+H]^+^: C_14_H_11_Br_2_ClN_3_O_2_^+^, calc: 445.8901; found: 445.8901, 447.8880, 449.8857, 451.8831 (3/7/5/1).
*2-Amino-3,5-dibromo-N*′*-(4-nitrobenzoyl)benzohydrazide* (**15**): Method B; yellow amorphous solid; mp: 283.5 °C (1,4-dioxane); yield: 69%; IR (KBr) cm^−1^: 3467, 3351, 3227, 3024, 1673, 1636, 1604; ^1^H-NMR (DMSO-*d*_6_, 500 MHz) *δ* 10.88 (brs, 1H, NH), 10.68 (brs, 1H, NH), 8.37 (d, *J* = 8.5 Hz, 2H), 8.14 (d, *J* = 8.5 Hz, 2H), 7.82 (s, 2H), 6.59 (brs, 2H, NH_2_) ppm; ^13^C-NMR (DMSO-*d*_6_, 125 MHz) *δ* 166.4, 164.4, 149.5, 145.6, 137.9, 137.2, 130.2, 129.1, 123.8, 115.6, 110.4, 105.2 ppm; HRMS(ESI) *m*/*z* [M+H]^+^: C_14_H_11_Br_2_N_4_O_4_^+^, calc: 456,9142; found: 456.9141, 458.9120, 460.9100 (1/2/1). Despite all our efforts, a small amount of 1,4-dioxane remained after recrystallization.
*N*′*-(2-Amino-3,5-dibromobenzoyl)furan-2-carbohydrazide* (**16**): Method B; beige amorphous solid; mp: 223.3 °C (EA/EtOH); yield: 54%; IR (KBr) cm^−1^: 3469, 3410, 3339, 3198, 1676, 1640, 1600; ^1^H-NMR (DMSO-*d*_6_, 500 MHz) *δ* 10.48 (bs, 1H, NH), 10.41 (s, 1H, NH), 7.92 (s, 1H), 7.80 (s, 1H), 7.78 (s, 1H), 7.27 (d, *J* = 3.1 Hz, 1H), 6.68 (s, 1H), 6.57 (brs, 2H, NH_2_) ppm; ^13^C-NMR (DMSO-*d*_6_, 125 MHz) *δ* 166.4, 157.3, 146.0, 145.9, 145.5, 137.0, 130.1, 115.7, 114.8, 111.9, 110.3, 105.2 ppm; HRMS(ESI) *m*/*z* [M+H]^+^: C_12_H_10_Br_2_N_3_O_3_^+^, calc: 401,9083; found: 401.9083, 403.9062, 405.9042 (1/2/1).
*2-Amino-N*′*-benzoyl-5-iodobenzohydrazide* (**17**): Method C (1.5 h + 2.5 h); off-white amorphous solid; mp: 208.0 °C (EA/EtOH); yield: 46%; IR (KBr) cm^−1^: 3417, 3297, 3274, 1673, 1641, 1603; ^1^H-NMR (DMSO-*d*_6_, 400 MHz) *δ* 10.42 (s, 1H, NH), 10.31 (brs, 1H, NH), 7.92 (d, *J* = 7.5 Hz, 2H), 7.91 (s, 1H), 7.60 (t, *J* = 7.3 Hz, 1H), 7.52 (t, *J* = 7.4 Hz, 2H), 7.44 (d, *J* = 8.6 Hz, 1H), 6.61 (d, *J* = 8.7 Hz, 1H), 6.58 (brs, 2H, NH_2_) ppm; ^13^C-NMR (DMSO-*d*_6_, 100 MHz) *δ* 167.0, 165.9, 149.4, 140.2, 136.0, 132.5, 131.9, 128.5, 127.5, 119.0, 114.9, 74.6 ppm; HRMS(ESI) *m*/*z* [M+H]^+^: C_14_H_13_IN_3_O_2_^+^, calc: 382.0047; found: 382.0046.
*N*′*-(2-Amino-5-iodobenzoyl)furan-2-carbohydrazide* (**18**): Method C (1.5 h + 1.5 h); beige amorphous solid; mp: 198.8 °C (EA/hex); yield: 48%; IR (KBr) cm^−1^: 3435, 3330, 3181, 1686, 1641, 1601; ^1^H-NMR (DMSO-*d*_6_, 500 MHz) *δ* 10.30 (s, 1H, NH), 10.24 (brs, 1H, NH), 7.91 (s, 1H), 7.87 (s, 1H), 7.45 (d, *J* = 8.6 Hz, 1H), 7.25 (s, 1H), 6.67 (s, 1H), 6.61 (d, *J* = 8.7 Hz, 1H), 6.57 (brs, 2H, NH_2_) ppm; ^13^C-NMR (DMSO-*d*_6_, 125 MHz) *δ* 167.0, 157.5, 149.5, 146.3, 145.8, 140.3, 136.0, 119.0, 114.7, 114.6, 111.9, 74.6 ppm; HRMS(ESI) *m*/*z* [M+H]^+^: C_12_H_11_IN_3_O_3_^+^, calc: 371.9840; found: 371.9836.
*2-Amino-N*′*-benzoyl-4-chlorobenzohydrazide* (**19**): Method C (1.5 h + 1.5 h); white amorphous solid; mp: 230.1 °C (EA/EtOH); yield: 58%; IR (KBr) cm^−1^: 3458, 3353, 3205, 1607, 1566; ^1^H-NMR (DMSO-*d*_6_, 500 MHz) *δ* 10.40 (s, 1H, NH), 10.26 (s, 1H, NH), 7.91 (d, *J* = 7.5 Hz, 2H), 7.62 (d, *J* = 8.6 Hz, 1H), 7.59 (t, *J* = 7.2 Hz, 1H), 7.51 (t, *J* = 7.5 Hz, 2H), 6.82 (s, 1H), 6.69 (brs, 2H, NH_2_), 6.58 (d, *J* = 8.4 Hz, 1H) ppm; ^13^C-NMR (DMSO-*d*_6_, 125 MHz) *δ* 167.5, 166.0, 151.2, 136.9, 132.6, 131.9, 130.0, 128.5, 127.5, 115.2, 114.4, 111.3 ppm; HRMS(ESI) *m*/*z* [M+H]^+^: C_14_H_13_ClN_3_O_2_^+^, calc: 290.0691; found: 290.0690, 292.0661 (3/1).
*N*′*-(2-Amino-4-chlorobenzoyl)furan-2-carbohydrazide* (**20**): Method C (1.5 h + 1.5 h); white amorphous solid; mp: 207.8 °C (EA/EtOH); yield: 57%; IR (KBr) cm^−1^: 3397, 3303, 1681, 1648, 1613; ^1^H-NMR (DMSO-*d*_6_, 500 MHz) *δ* 10.28 (s, 1H, NH), 10.21 (s, 1H, NH), 7.91 (s, 1H), 7.59 (d, *J* = 8.4 Hz, 1H), 7.25 (s, 1H), 6.81 (s, 1H), 6.68 (brs, 3H, 1H + NH_2_), 6.57 (d, *J* = 8.3 Hz, 1H) ppm; ^13^C-NMR (DMSO-*d*_6_, 100 MHz) *δ* 167.6, 157.6, 151.3, 146.3, 145.8, 136.9, 130.0, 115.2, 114.6, 114.4, 111.9, 111.0 ppm; HRMS(ESI) *m*/*z* [M+H]^+^: C_12_H_11_ClN_3_O_3_^+^, calc: 280.0483; found: 280.0483, 280.0452 (3/1).
*2-Amino-N*′*-benzoyl-4-nitrobenzohydrazide* (**21**): Method C (1.5 h + 3 h); yellow amorphous solid; mp: 244.4 °C (EA/EtOH); yield: 45%; IR (KBr) cm^−1^: 3406, 3275, 1671, 1646, 1622; ^1^H-NMR (DMSO-*d*_6_, 500 MHz) *δ* 10.53 (s, 2H, NH, NH), 7.92 (d, *J* = 7.5 Hz, 2H), 7.90 (s, 1H), 7.78 (d, *J* = 8.5 Hz, 1H), 7.63 (s, 1H), 7.60 (t, *J* = 7.8 Hz, 1H), 7.53 (t, *J* = 7.4 Hz, 2H), 7.34 (d, *J* = 8.5 Hz, 1H), 6.87 (brs, 2H, NH_2_) ppm; ^13^C-NMR (DMSO-*d*_6_, 125 MHz) *δ* 166.9, 166.0, 150.3, 149.8, 132.4, 132.0, 129.8, 128.6, 127.5, 118.1, 110.2, 108.4 ppm; HRMS(ESI) *m*/*z* [M+H]^+^: C_14_H_13_N_4_O_4_^+^, calc: 301.0931; found: 301.0932.
*N*′*-(2-Amino-4-nitrobenzoyl)furan-2-carbohydrazide* (**22**): Method C (1.5 h + 6 h); yellow amorphous solid; mp: 242.0 °C (EA/EtOH); yield: 47%; IR (KBr) cm^−1^: 3446, 3366, 3212, 3129, 1682, 1627, 1564; ^1^H-NMR (DMSO-*d*_6_, 500 MHz) *δ* 10.47 (s, 1H, NH), 10.42 (s, 1H, NH), 7.92 (s, 1H), 7.75 (d, *J* = 8.6 Hz, 1H), 7.62 (s, 1H), 7.32 (d, *J* = 8.6 Hz, 1H), 7.26 (d, *J* = 2.1 Hz, 1H), 6.85 (brs, 2H, NH_2_), 6.68 (dd, *J* = 3.0, 1.5 Hz, 1H) ppm; ^13^C-NMR (DMSO-*d*_6_, 125 MHz) *δ* 166.9, 157.5, 150.3, 149.9, 146.2, 145.9, 129.8, 117.8, 114.8, 112.0, 110.2, 108.4 ppm; HRMS(ESI) *m*/*z* [M+H]^+^: C_12_H_11_N_4_O_5_^+^, calc: 291.0724; found: 291.0725.
*2-Amino-N*′*-benzoyl-5-nitrobenzohydrazide* (**23**): Method C (1.5 h + 12 h); yellow amorphous solid; mp: 311.1 °C (1,4-dioxane/EtOH); yield: 20%; IR (KBr) cm^−1^: 3398, 3362, 3297, 3263, 3177, 1689, 1645, 1619; ^1^H-NMR (DMSO-*d*_6_, 500 MHz) *δ* 10.68 (s, 1H, NH), 10.51 (s, 1H, NH), 8.64 (s, 1H), 8.08 (d, *J* = 9.0 Hz, 1H), 7.92 (d, *J* = 7.5 Hz, 2H), 7.72 (brs, 2H, NH_2_), 7.61 (t, *J* = 7.3 Hz, 1H), 7.53 (t, *J* = 7.4 Hz, 2H), 6.86 (d, *J* = 9.2 Hz, 1H), ppm; ^13^C-NMR (DMSO-*d*_6_, 75 MHz) *δ* 166.8, 166.0, 155.3, 135.0, 132.4, 132.0, 128.6, 127.9, 127.5, 126.0, 116.1, 111.0 ppm; HRMS(ESI) *m*/*z* [M+H]^+^: C_14_H_13_N_4_O_4_^+^, calc: 301.0931; found: 301.0932.
*N*′*-(2-Amino-5-nitrobenzoyl)furan-2-carbohydrazide* (**24**): Method C (1.5 h + 12 h); yellow amorphous solid; mp: >350 °C (1,4-dioxane/EtOH); yield: 26%; IR (KBr) cm^−1^: 3401, 3294, 3253, 1688, 1643, 1618; ^1^H-NMR (DMSO-*d*_6_, 300 MHz) *δ* 10.64 (s, 1H, NH), 10.41 (s, 1H, NH), 8.61 (d, *J* = 2.6 Hz, 1H), 8.08 (dd, *J* = 9.3, 2.6 Hz, 1H), 7.94 (s, 1H), 7.73 (brs, 2H, NH_2_), 7.27 (d, *J* = 3.5 Hz, 1H), 6.86 (d, *J* = 9.3 Hz, 1H), 6.69 (s, 1H) ppm; ^13^C-NMR (DMSO-*d*_6_, 75 MHz) *δ* 166.8, 157.4, 155.3, 146.2, 145.9, 135.0, 128.0, 126.0, 116.1, 114.8, 112.0, 110.7 ppm; HRMS(ESI) *m*/*z* [M+H]^+^: C_12_H_11_N_4_O_5_^+^, calc: 291.0724; Found: 291.0729.

### 3.2. DNA Photo-Cleavage Experiments

The compounds **1**–**24** were individually incubated with plasmid DNA at the desired concentration, in Eppendorf vials and/or were irradiated with UV-B (312 nm, 2 × 9 W) or UV-A (365 nm, 2 × 9 W), and in 10 cm distance under aerobic conditions at room temperature for 30 min and 2 h, respectively. The conditions of the photobiological reaction and gel electrophoresis, quantification of DNA-cleaving activity and calculation of ss % and ds % damage protocols have been described previously [72]. All experiments were performed at least twice.

### 3.3. Molecular Docking Studies

Organic compounds were fully optimized for their minimized energy at the B3LYP/6-31g* level of theory with the LanL2DZ basis set for iodine in the case of the compounds **13** and **18** as implemented in the Gaussian 09 [73] suite of programs (Revision B.01). The crystal data of the B-DNA dodecamer d(CGCGAATTCGCG)2 (PDB 1D:1BNA) were downloaded from the Protein Data Bank [74]. The docking analysis was performed using the AutoDock Vina program [75] (https:vina.scripps.edu, accessed on 21 January 2024). The DNA was adapted for docking by removing water molecules and polar hydrogens, and Gasteiger charges were added by AutoDock 4.2 Tools (ADT) before performing docking calculations. A grid box with a size of 60 × 80 × 114 with 0.375 Å spacing was used to encompass the whole DNA. The rigid docking protocol and 100 runs of the Lamarckian genetic algorithm for searching ligand conformations were performed. PyMOL [76] was used for the representation of the docking results and interactions between DNA and compounds.

### 3.4. Cell Culture Experiments

The CarB cell line, from mouse skin squamous cell carcinoma was a kind gift from V. Zoumpourlis, from National Hellenic Research Foundation, and was used to test the cytotoxic effect of the compounds. Cells were cultured under aseptic conditions using DMEM basal medium (31885-023; Gibco, Grand Island, NY, USA) supplemented with 10% fetal bovine serum (FB1000/500, Biosera, East Sussex, UK), 100 units/mL penicillin and 100 μg/mL streptomycin (15140-122, Gibco) and 2 mM L-Glutamine (25030; Gibco). The cell line was maintained at standard conditions (37 °C, 5% CO_2_) in a humidified atmosphere and cells were used at 70–90% confluency. A total of 10,000 cells were seeded per well. A UV-A lamp was placed 10 cm over the 96-well plate. A 1 h incubation with 100 μM and 200 μM of each compound was followed by 1 h irradiation with UV-A (365 nm). Then, compounds were removed, the medium was replaced and a cytotoxicity assay was performed 24 h later. The Resazurin Cell Viability Assay (CA035, Canvax, Boecillo, Spain) was used for fluorescence measurements according to the manufacturer’s guidelines. Essentially, a non-irradiated 96-well plate was used as a control, under the same conditions. Incubation with 10% resazurin (7 h) was followed by fluorescence measurement at λ_em_ = 590 nm and λ_ex_ = 530/560 nm in a VarioSkan lux reader (ThermoFisher Scientific, Waltham, MA, USA).

## 4. Conclusions

Due to the importance of diacylhydrazine bridged anthranilic acids, a series of such compounds have been synthesized from the conjugation of commercially available anthranilic acids and hydrazides. The counterparts were carefully chosen to possess substituents with all possible electronic effects. The electron-withdrawing effect of the NO_2_ group in the *p*-position in relation to the amine group of the AA negatively affected the yield of the products, in a method driven by the in situ formation of the anthraniloyl chloride. For all new AA DACHZ derivatives, the yields were calculated based on the amount of product precipitated upon treatment with CH_2_Cl_2_ and were moderate; however, the synthesis was completed in short times compared to other methods tried. All derivatives exhibited a high UV–Vis absorption in the UV-A area of the spectrum and DNA photocleavage was performed under both UV-B and UV-A irradiation. All screenings highlighted the importance of a halogen in the *p*-position in relation to the amine group and the absence of an electron-withdrawing group on the aryl group. Differences were observed in DNA photocleavage under UV-B and UV-A irradiation. The derivative **9** maintained activity under both types of irradiation at very low concentrations (1 and 2 μΜ, for UV-B and UV-A, respectively); however under UV-A irradiation, all halogenated compounds were active at concentrations as low as 2 and 10 μΜ. Molecular docking studies with DNA showed potential interaction sites, although the reactivity was not correlated for all derivatives with their photoactivity towards DNA. Cytotoxicity experiments indicated the iodo derivative **17** as a potent cytotoxic agent and the bromo compound **9** as a slight phototoxic agent. In general, based on the studies described herein, one may keep in mind the high UV-A light absorption of AA DACHZs that allows DNA photocleavage. Since no DNA photocleavage may occur without the compound showing binding to DNA, this new class of DNA photocleavers may hold promise for the development of novel anticancer, antimicrobial and probably insecticidal agents. Finally, open-form anthranilic acid derivatives and their rigid form quinazolinones exhibited very different photoreactivities particularly regarding derivatives containing the nitro group, with the former being inactive and the latter being highly potent DNA photocleavers.

## Figures and Tables

**Table 1:** Calculated energies and interactions of the compounds **1**–**24** with DNA.

Compound	Energy (Kcal/mol)	Interactions (PyMol) Polar Contacts
**1**	−8.9	DG4, DA5, DG22
**2**	−9.0	DG4, DA5, DG22
**3**	−8.1	DG10, DC11, DG14, DG16
**4**	−8.9	DG10, DC11, DC15, DG16
**5**	−9.5	DG10, DC11
**6**	−9.0	DC15, DG16
**7**	−9.8	DG16
**8**	−9.2	DG10, DC15, DG16
**9**	−8.8	DC15, DG16
**10**	−9.0	DC15, DG16
**11**	−9.4	DG10, DC11, DC15, DG16
**12**	−9.0	DG10, DC11, DC15, DG16
**13**	−9.2	DC11
**14**	−9.3	DG10, DC11
**15**	−10.1	DG10, DC11
**16**	−8.9	DG10, DG16
**17**	−9.4	DG10, DC15, DG16
**18**	−9.2	DG10, DC15, DG16
**19**	−9.6	DG10, DC11
**20**	−9.5	DG10, DG16
**21**	−9.9	DG10, DG16
**22**	−9.5	DG10, DC11, DC15, DG16
**23**	−9.8	DG10, DG16
**24**	−9.4	DG10, DC11, DG14, DG16

## Data Availability

Data are contained within the article or Supplementary Material.

## Supplementary Materials

The following supporting information can be downloaded at: https://www.mdpi.com/article/10.3390/molecules29030647/s1, S.1: Copies of NMR spectra of the compounds **1**–**24** and of HRMS measurements; S.2: Gel electrophoresis pictures of the compounds **1**–**24** and AA **IIa**–**h** (UV-B); S.3: Gel electrophoresis pictures of the compound **9**. Concentration, pH and mechanistic studies (UV-B); S.4: UV–Vis spectra of the compounds **1**–**24**; S.5: Gel electrophoresis pictures of the compounds **1**–**24** (UV-A); S.6: Optimized molecular geometries of the compounds **1**–**24** at the B3LYP/6–31G (d) level; S.7: Molecular docking studies for the compounds **1**–**24**.
