# Supplementary material for: Synthesis of 2-Amino-N′-aroyl(het)arylhydrazides, DNA Photocleavage, Molecular Docking and Cytotoxicity Studies against Melanoma CarB Cell Lines
Source: Molecules. 2024 Jan 30;29(3):647. doi: 10.3390/molecules29030647 (PMC10856246; doi:10.3390/molecules29030647)
Supplement: Supplementary file 1 [file molecules-29-00647-s001.zip › molecules-2826255-supplementary.pdf]

Supporting Information

Synthesis of 2-Amino-*N′*-aroyl(het)arylhydrazides, DNA Photocleavage, Molecular Docking and Cytotoxicity Studies Against Melanoma CarB Cell Lines

Achilleas Mitrakas[1], Maria-Eleni Stathopoulou[2], Chrysoula Mikra[3], Chrystalla Konstantinou[2], Stergios Rizos[4], Stella Malichetoudi[1], Alexandros E. Koumbis[3], Maria Koffa[1] and Konstantina C. Fylaktakidou[2,3,*]

[1] Laboratory of Cellular Biology and Cell Cycle, Molecular Biology and Genetics Department, Democritus University of Thrace, University Campus, Dragana, 68100 Alexandroupolis, Greece; amitrak@med.duth.gr (A.M.); stelmali1@mbg.duth.gr (S.M.); mkoffa@mbg.duth.gr (M.K.)

[2] Laboratory of Organic, Bioorganic and Natural Product Chemistry, Molecular Biology and Genetics Department, Democritus University of Thrace, 68100 Alexandroupolis, Greece; marialenastathopoulou@outlook.com (M.-E.K.S.); chrystallakon@outlook.com (C.K.)

[3] Laboratory of Organic Chemistry, Faculty of Chemistry, Aristotle University of Thessaloniki, GR-54124 Thessaloniki, Greece; chrmikgeo@chem.auth.gr (C.M.); akoumbis@chem.auth.gr (A.E.K.)

[4] Department of Chemistry and Chemical Biology, Harvard University, 12 Oxford St., Cambridge, MA 02138, USA; stergiosrizos@fas.harvard.edu

* Correspondence: kfylakta@chem.auth.gr

Table of contents

S.1	*Copies of NMR spectra of compounds 1-24 and HRMS measurements*	S3
S.2	*Gel electrophoresis pictures of compounds 1-24 and AA IIa-h (UV-B)*	S51
S.3	*Gel electrophoresis pictures of compound 9. Concentration, pH, mechanistic studies (UV-B)*	S55
S.4	*UV-Vis Spectra of compounds 1-24*	S57
S.5	*Gel electrophoresis pictures of compounds 1-24 (UV-A)*	S60
S.6	*Optimized molecular geometries of compounds 1-24 at B3LYP/6-31G (d) level*	S64
S.7	*Molecular Docking Studies for the compounds 1-24*	S66

S.1. Copies of NMR spectra of compounds 1-24 and HRMS measurements

2-amino-*N′*-benzoylbenzohydrazide (**1**)

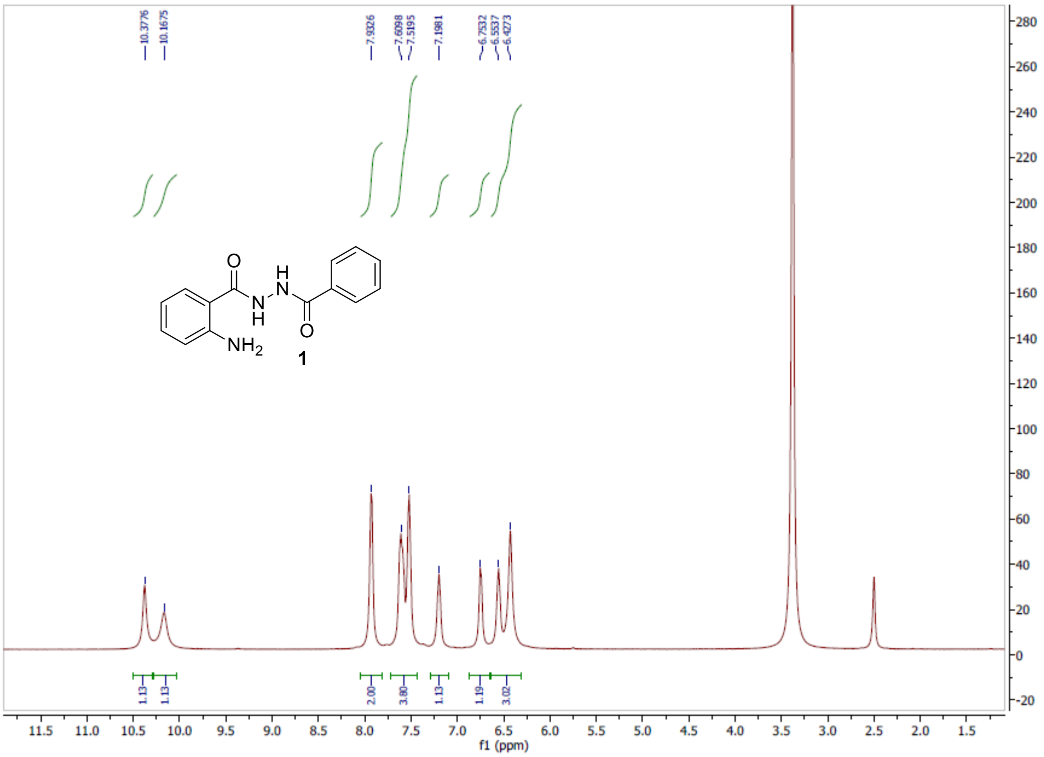

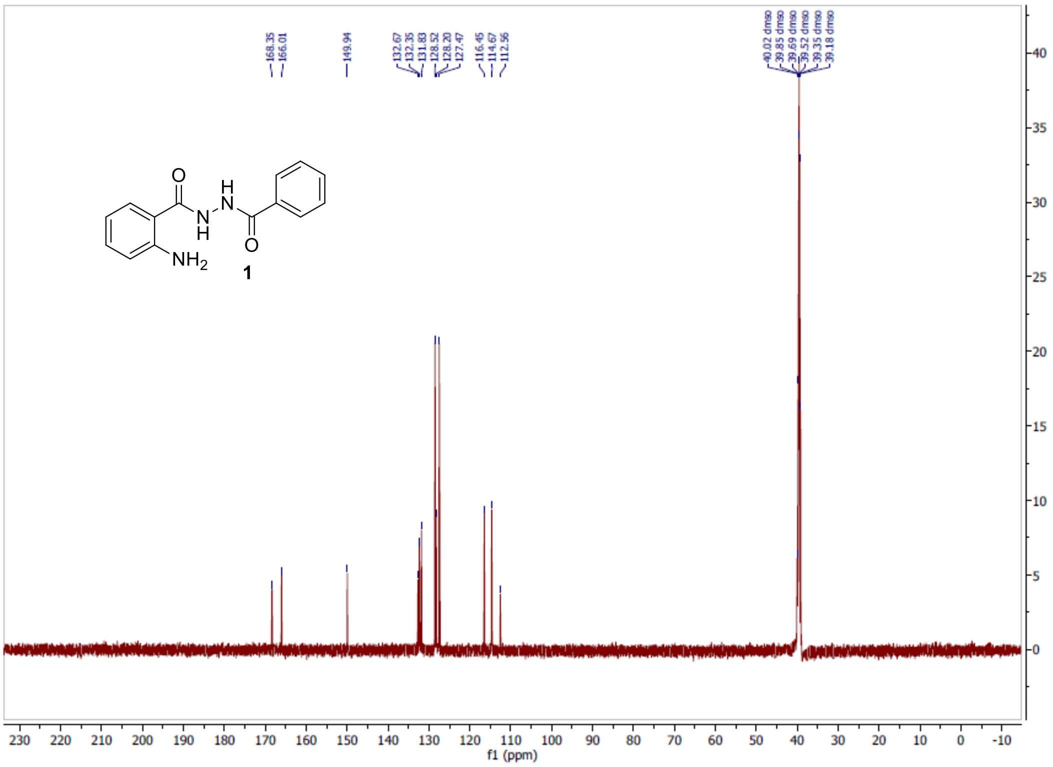

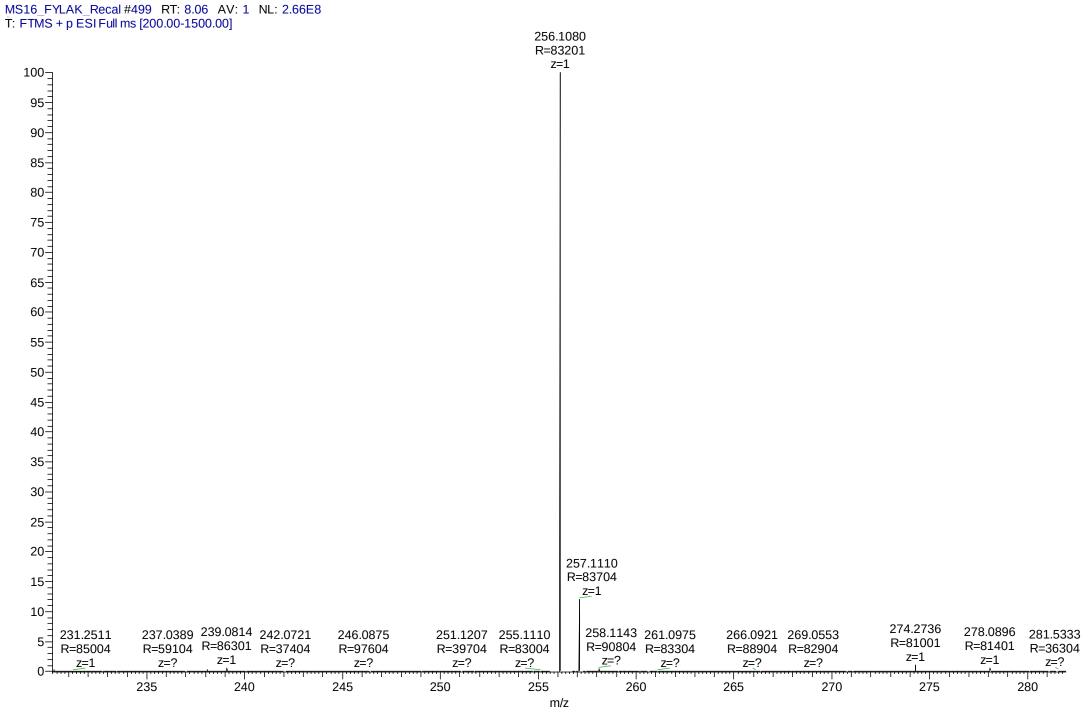

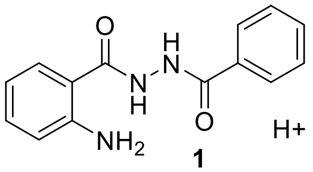

1

Chemical Formula: $C_{14}H_{14}N_3O_2^+$
Exact Mass: 256,1081

2-amino-*N*′-(4-chlorobenzoyl)benzohydrazide (**2**)

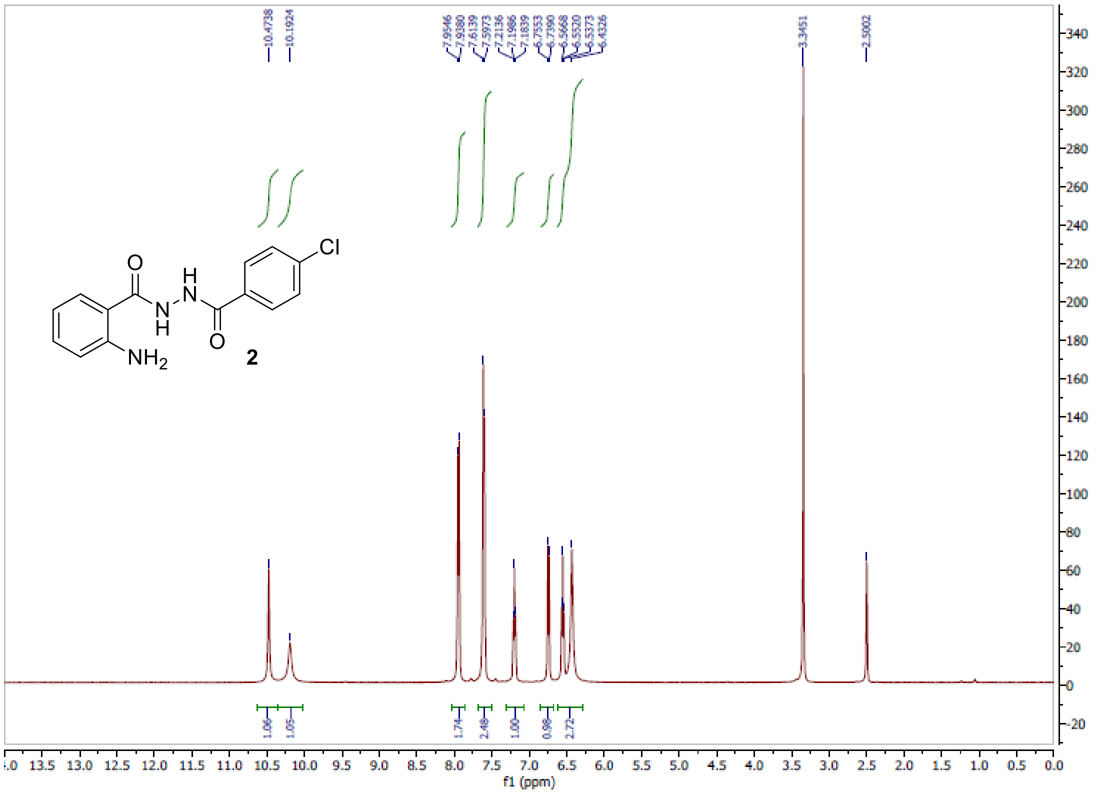

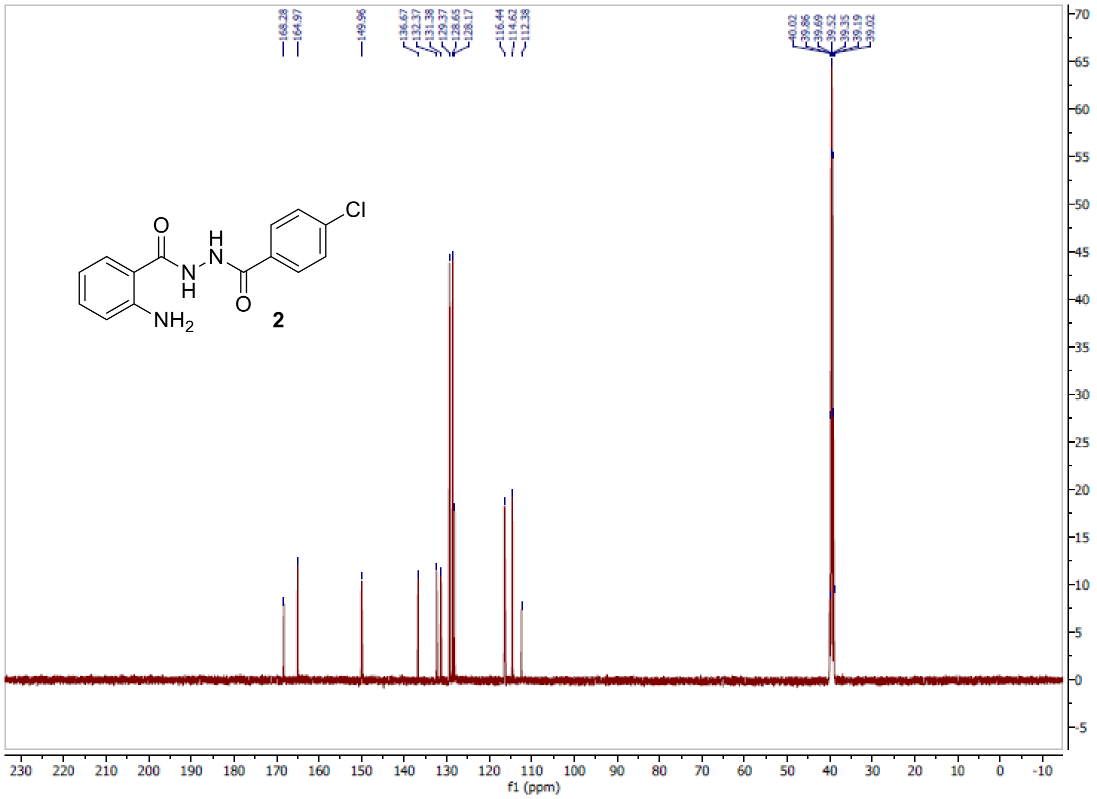

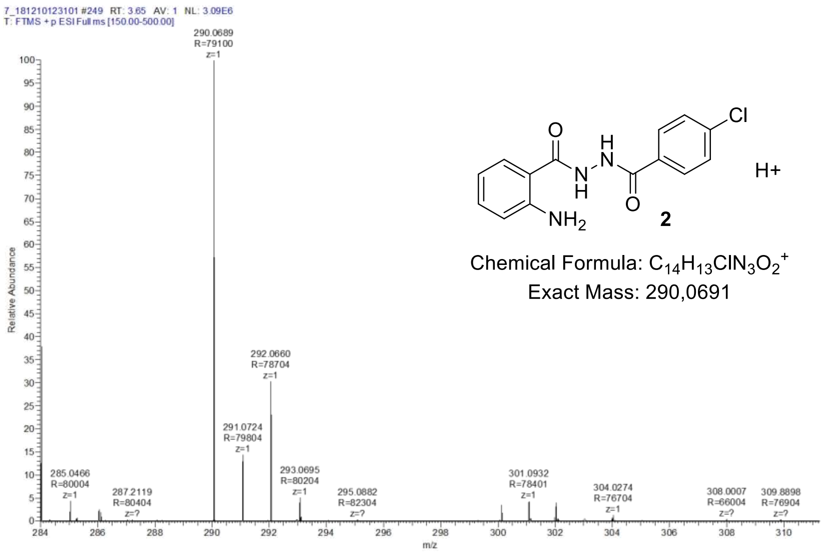
7_181210123101 #249 RT: 3.65 AV: 1 NL: 3.09E6
T: FTMS + p ESI Full ms [150.00-500.00]
290.0689
R=79100
z=1
292.0660
R=78704
z=1
291.0724
R=79804
z=1
285.0466
R=80004
z=1
287.2119
R=80404
z=?
293.0695
R=80204
z=1
295.0882
R=82304
z=?
301.0932
R=78401
z=1
304.0274
R=76704
z=1
308.0007
R=66004
z=?
309.8898
R=76904
z=?
Relative Abundance
m/z
H+
2
Chemical Formula: $C_{14}H_{13}ClN_3O_2^+$
Exact Mass: 290,0691

2-amino-*N′*-(4-nitrobenzoyl)benzohydrazide (**3**)

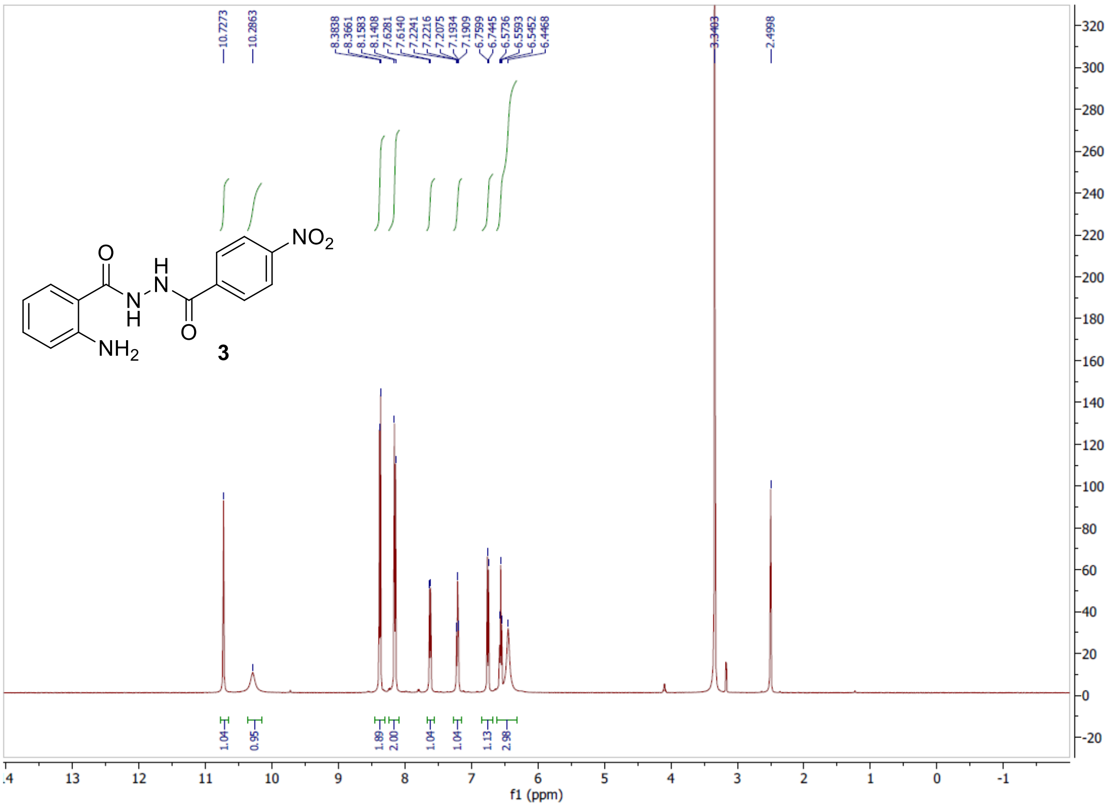

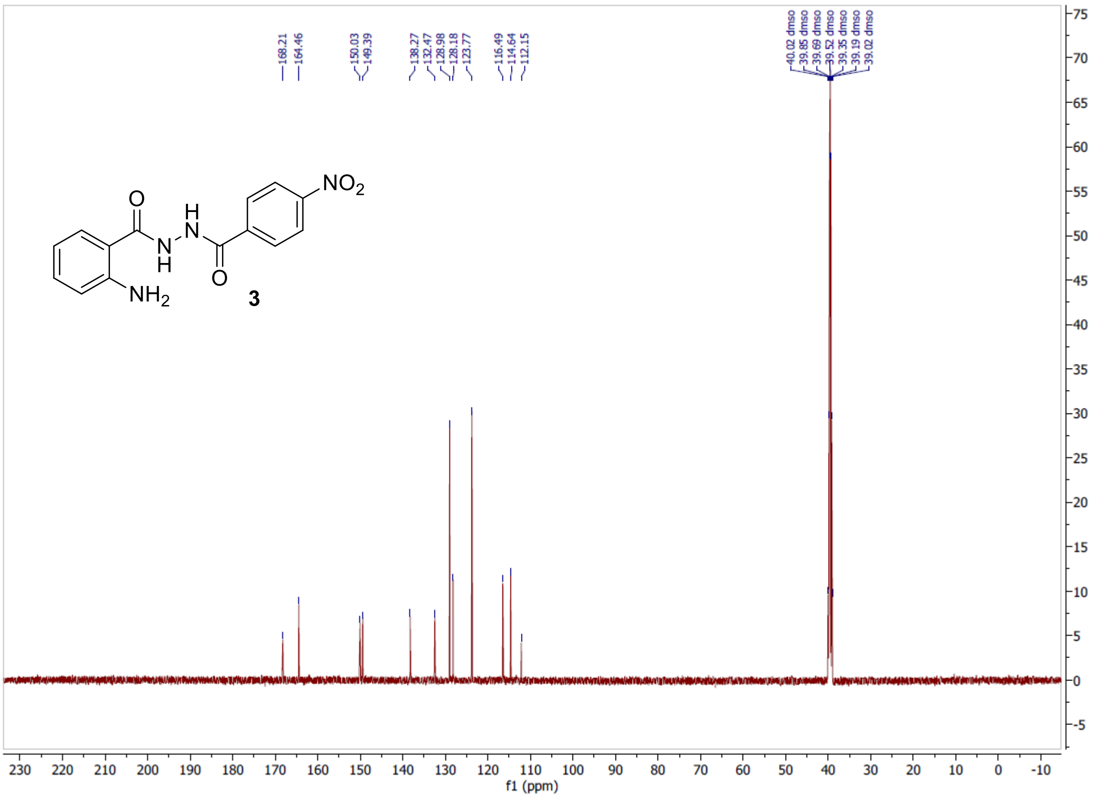

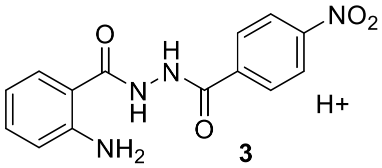

3

Chemical Formula: $C_{14}H_{13}N_4O_4^+$
Exact Mass: 301,0931

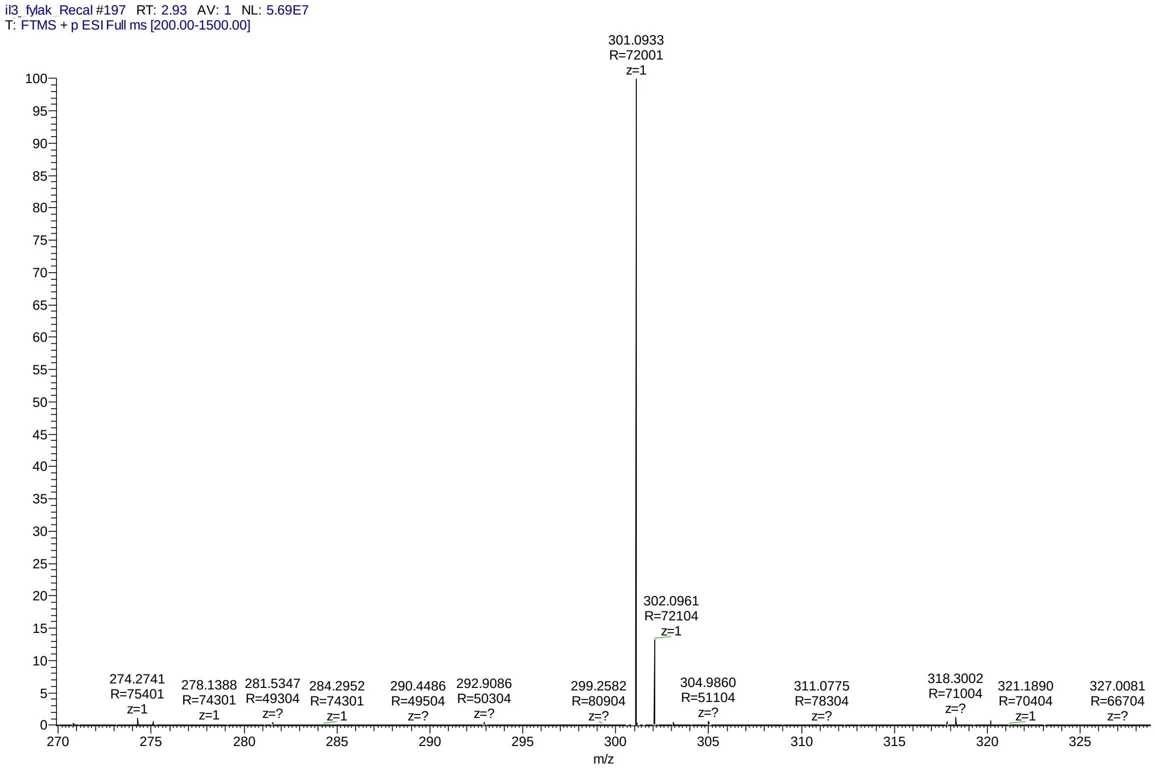

N'-(2-aminobenzoyl)furan-2-carbohydrazide (**4**)

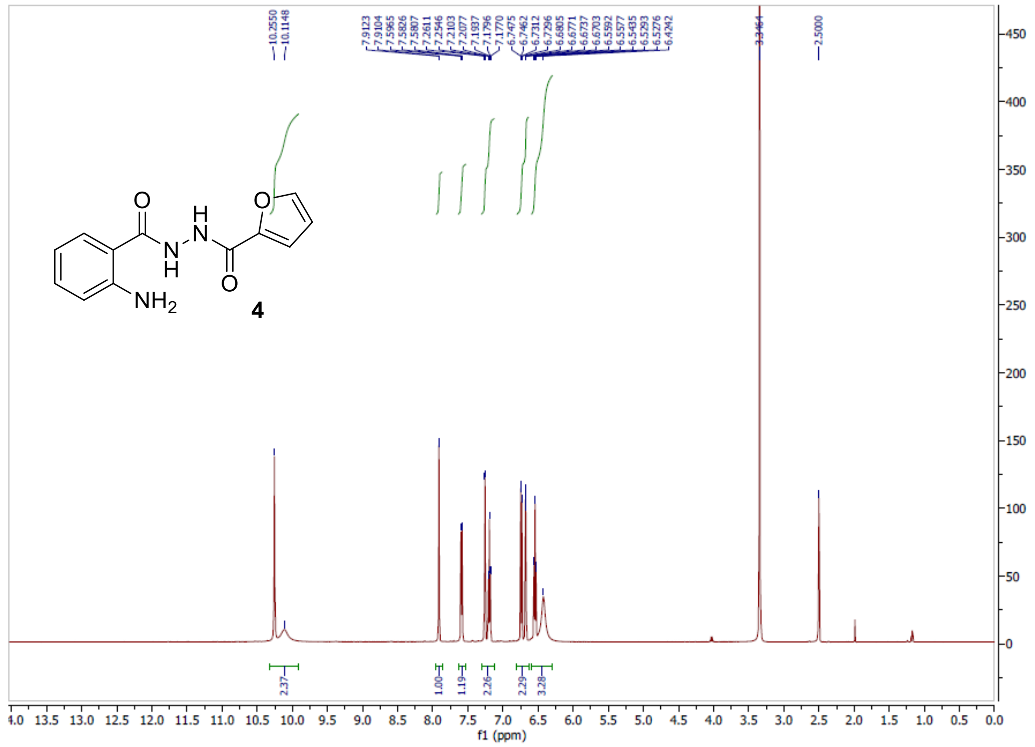

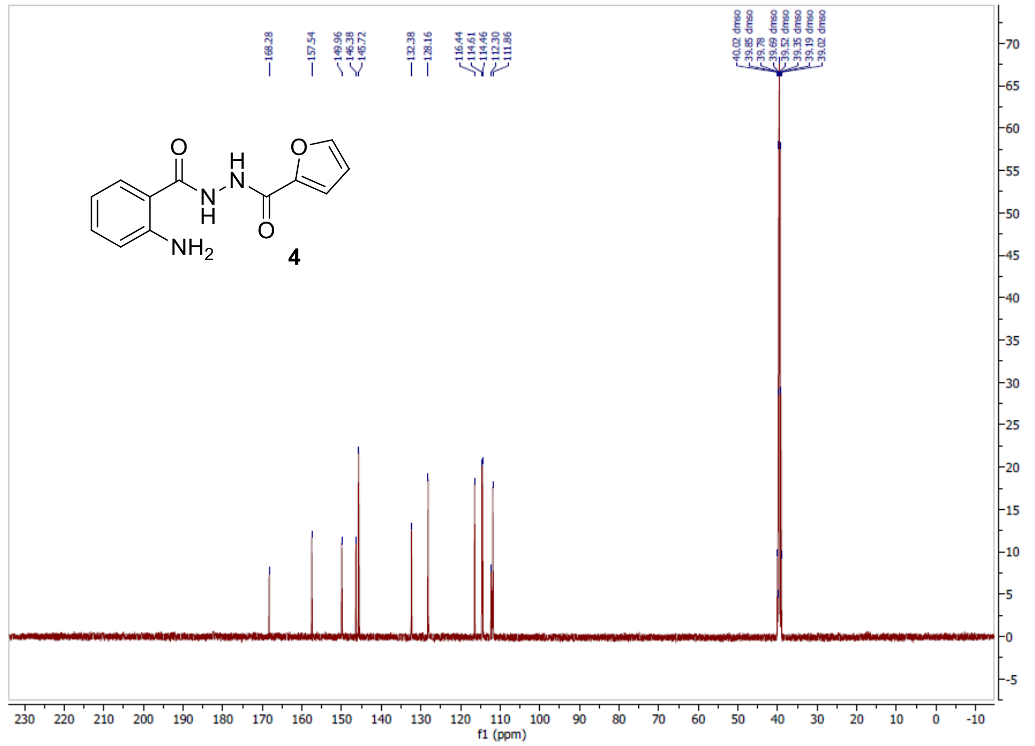

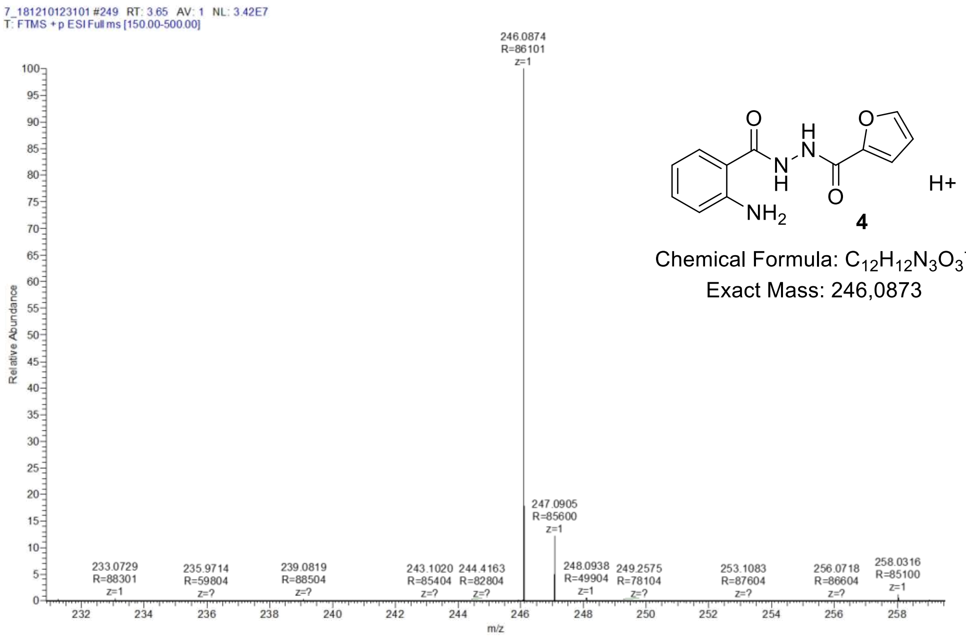

H+

4

Chemical Formula: $C_{12}H_{12}N_3O_3^+$
Exact Mass: 246,0873

2-amino-*N′*-benzoyl-5-chlorobenzohydrazide (**5**)

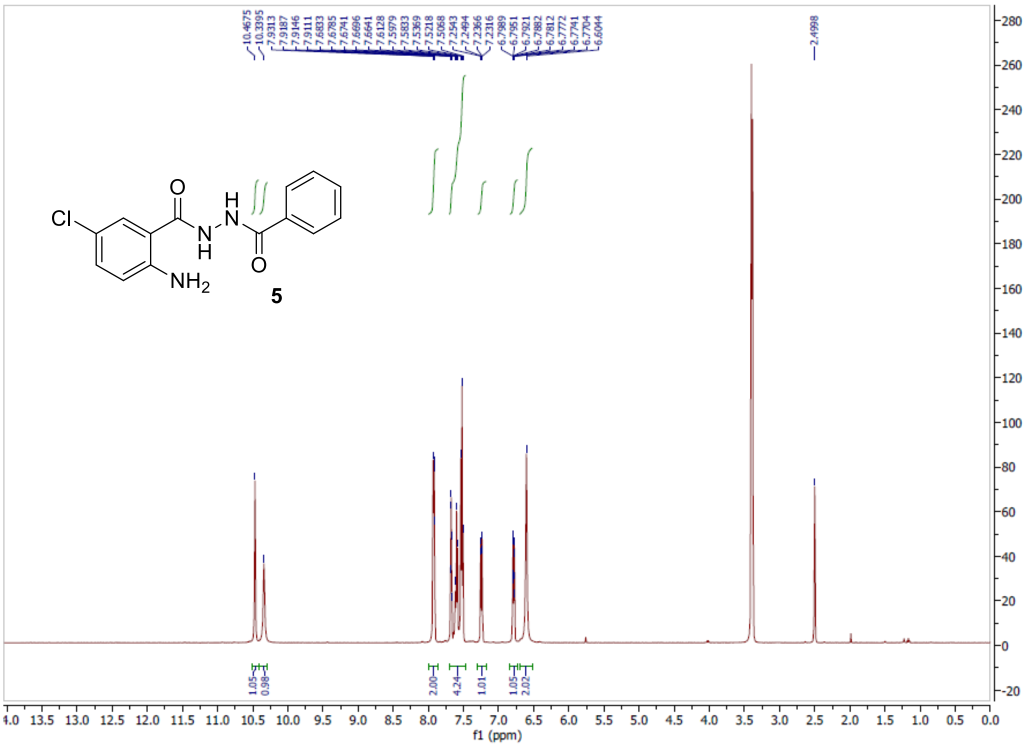

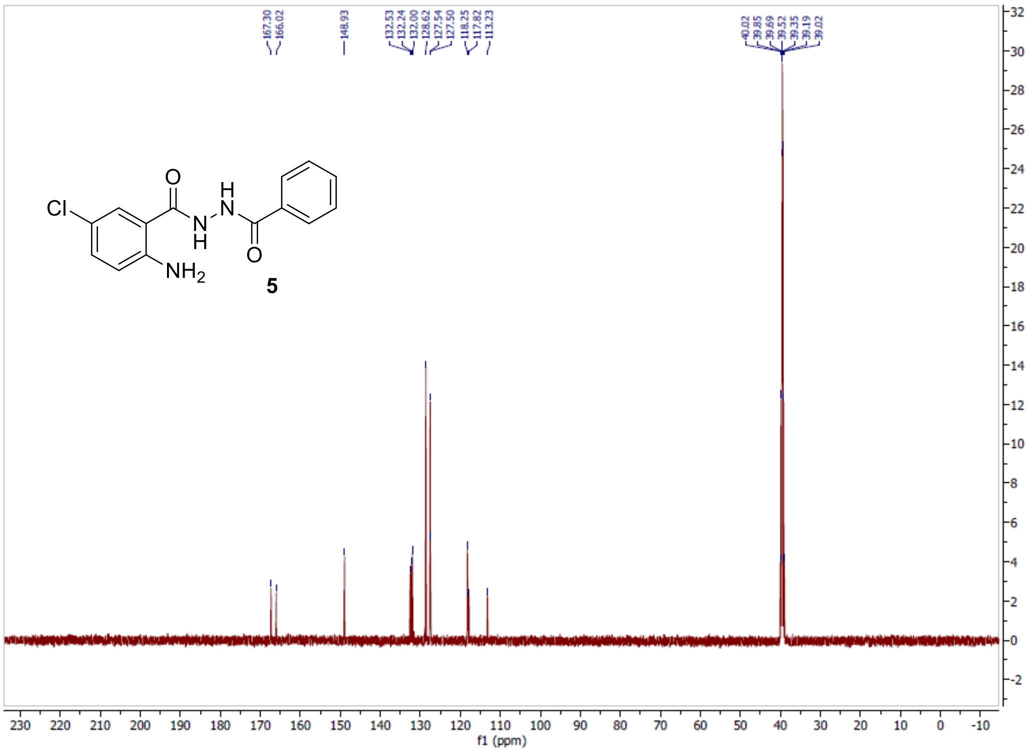

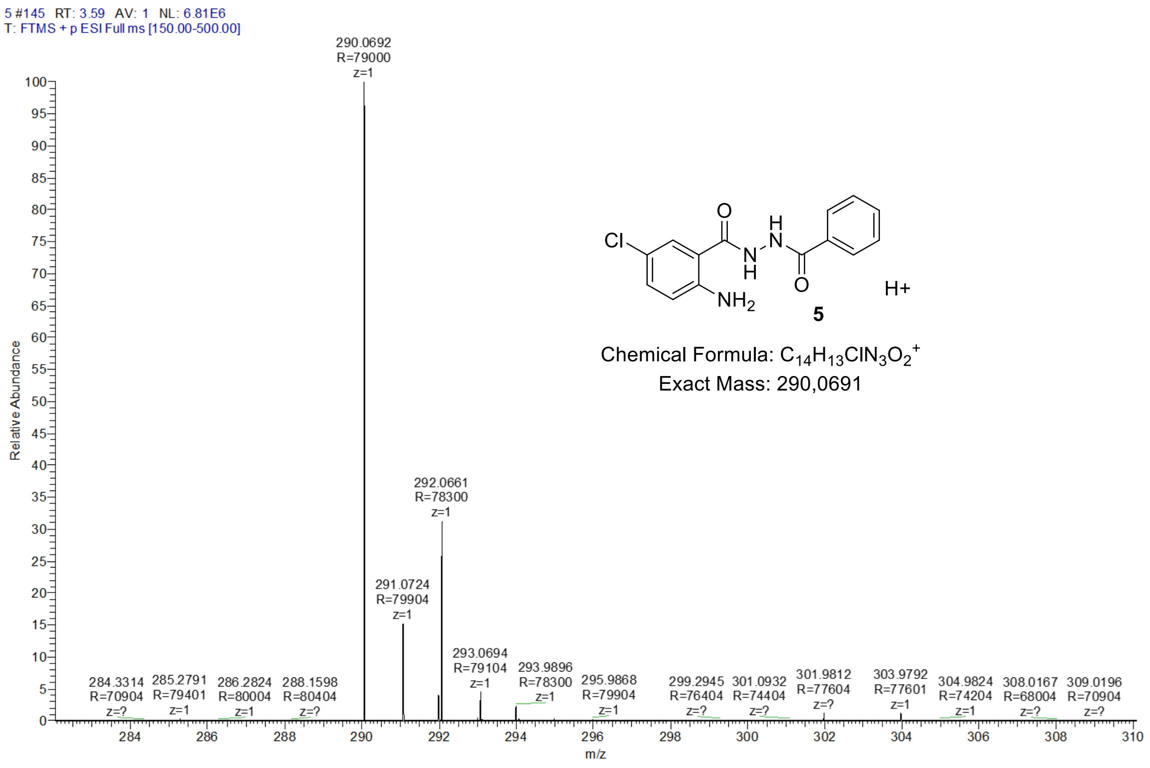
5 #145 RT: 3.59 AV: 1 NL: 6.81E6
T: FTMS + p ESI Full ms [150.00-500.00]
290.0692
R=79000
z=1
292.0661
R=78300
z=1
291.0724
R=79904
z=1
293.0694
R=79104
z=1
284.3314 285.2791 286.2824 288.1598 293.9896 295.9868 299.2945 301.0932 301.9812 303.9792 304.9824 308.0167 309.0196
Relative Abundance
m/z
Cl
NH2
H+
5
Chemical Formula: C14H13ClN3O2+
Exact Mass: 290,0691

2-amino-5-chloro-*N′*-(4-chlorobenzoyl)benzohydrazide (**6**)

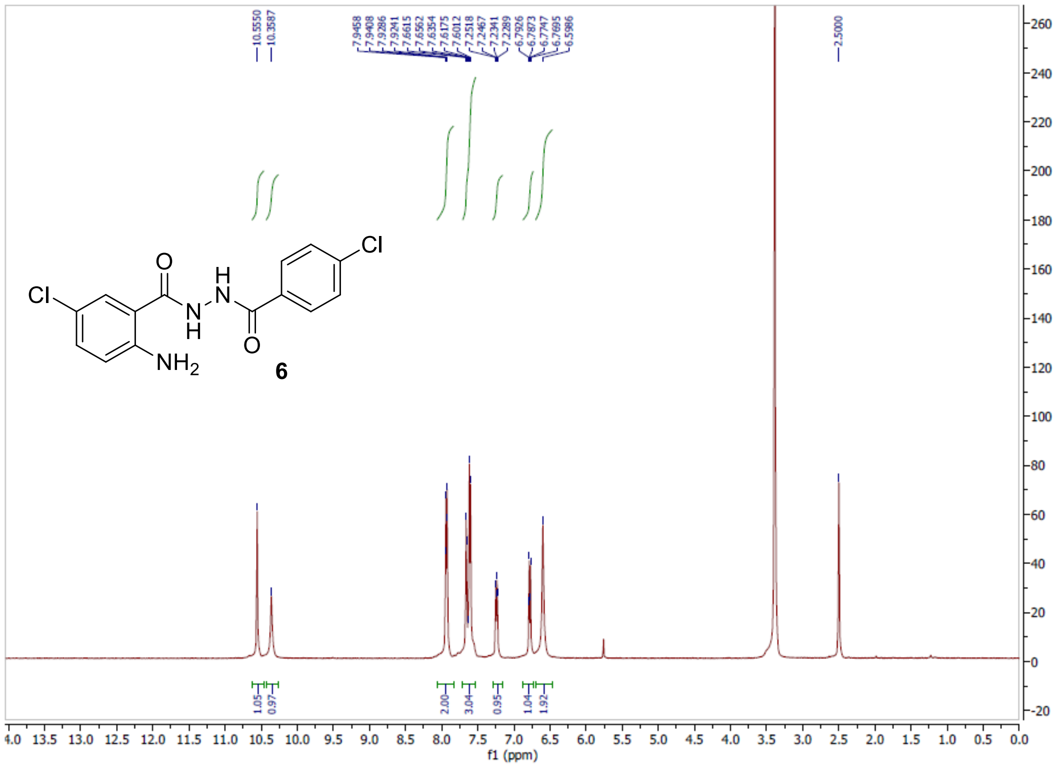

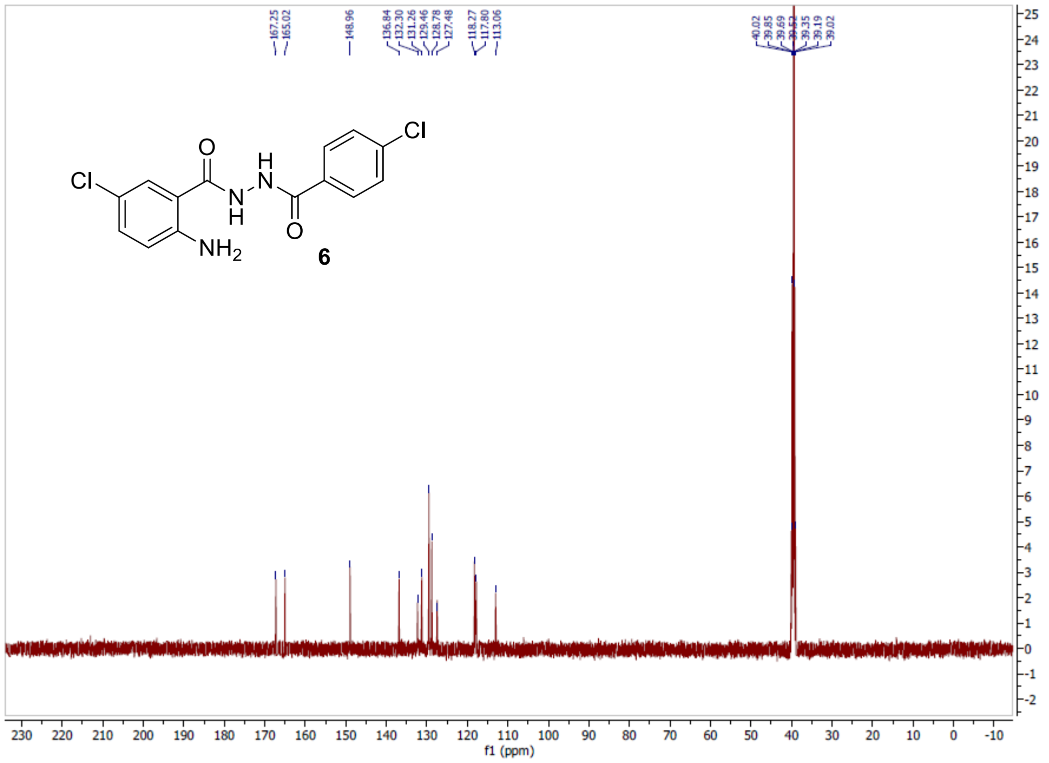

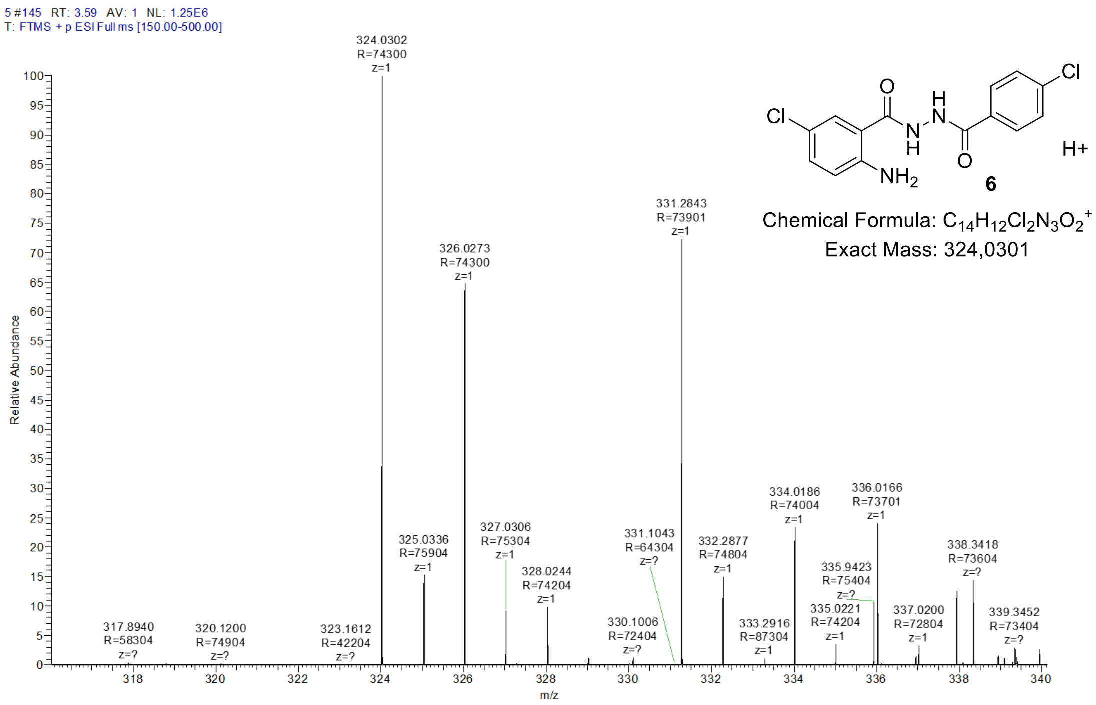

Chemical Formula: $C_{14}H_{12}Cl_2N_3O_2^+$
Exact Mass: 324,0301

2-amino-5-chloro-*N′*-(4-nitrobenzoyl)benzohydrazide (**7**)

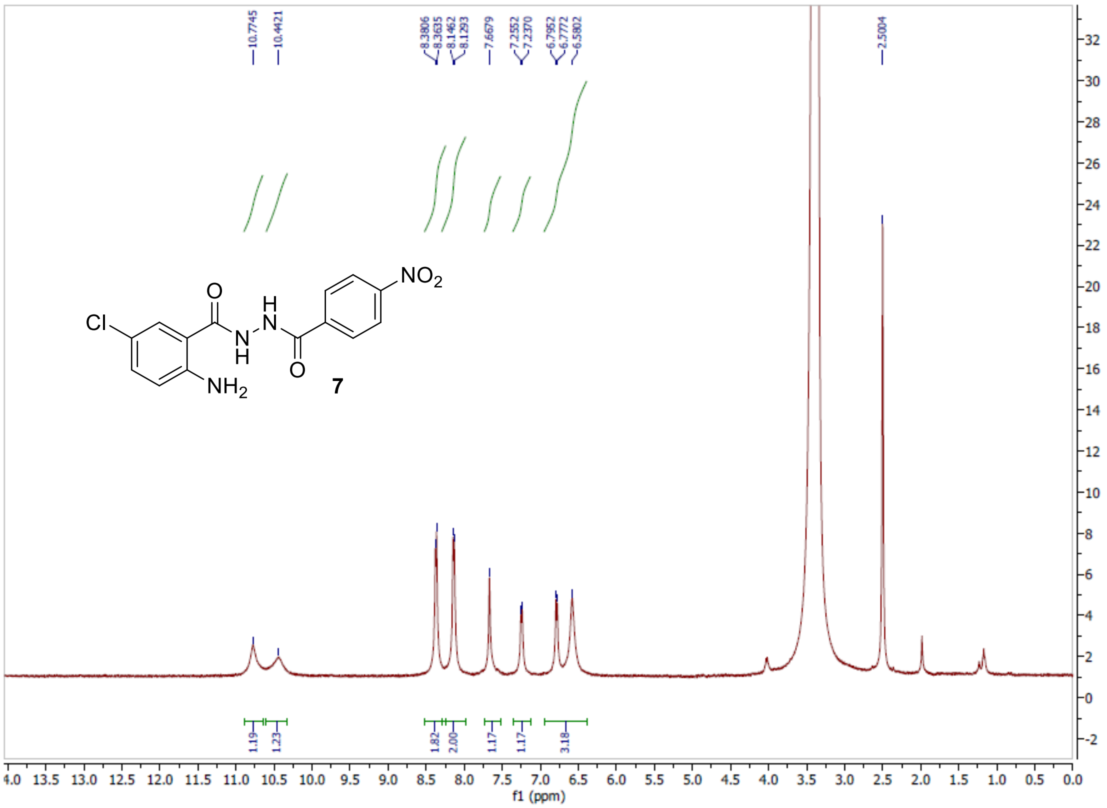

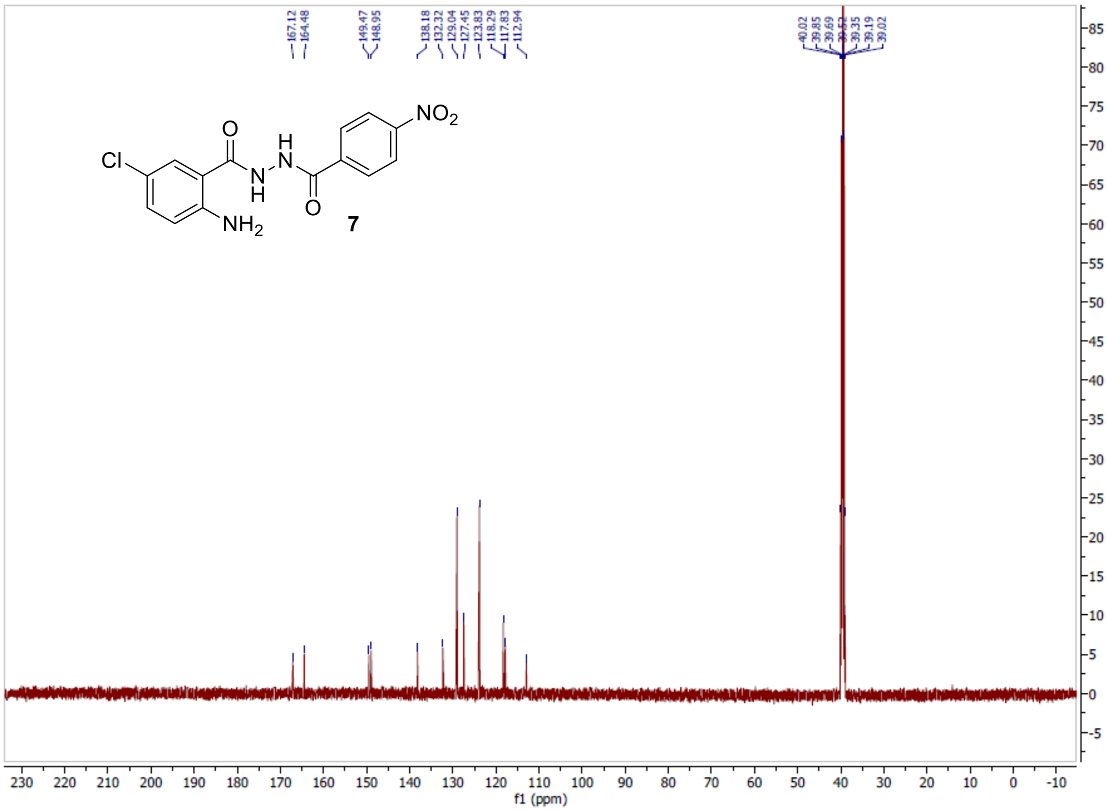

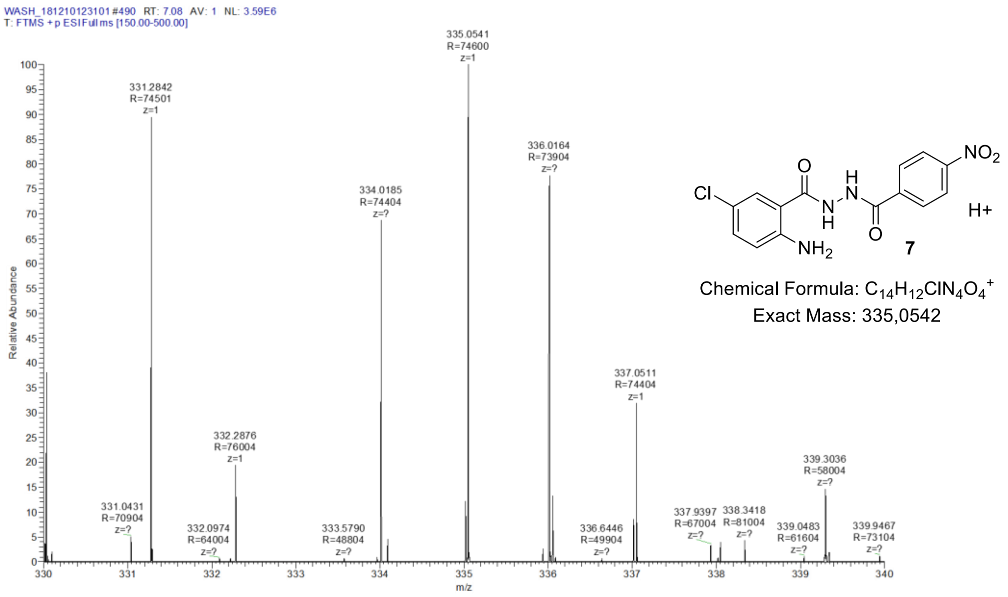
WASH_181210123101 #490 RT: 7.08 AV: 1 NL: 3.59E6
T: FTMS + p ESI Full ms [150.00-500.00]
335.0541
R=74600
z=1
331.2842
R=74501
z=1
336.0164
R=73904
z=?
334.0185
R=74404
z=?
337.0511
R=74404
z=1
332.2876
R=76004
z=1
339.3036
R=58004
z=?
331.0431
R=70904
z=?
332.0974
R=64004
z=?
333.5790
R=48804
z=?
336.6446
R=49904
z=?
337.9397
R=67004
z=?
338.3418
R=81004
z=?
339.0483
R=61604
z=?
339.9467
R=73104
z=?
Relative Abundance
m/z
Cl
NO2
NH2
H+
7
Chemical Formula: $C_{14}H_{12}ClN_4O_4^+$
Exact Mass: 335,0542

N′-(2-amino-5-chlorobenzoyl)furan-2-carbohydrazide (**8**)

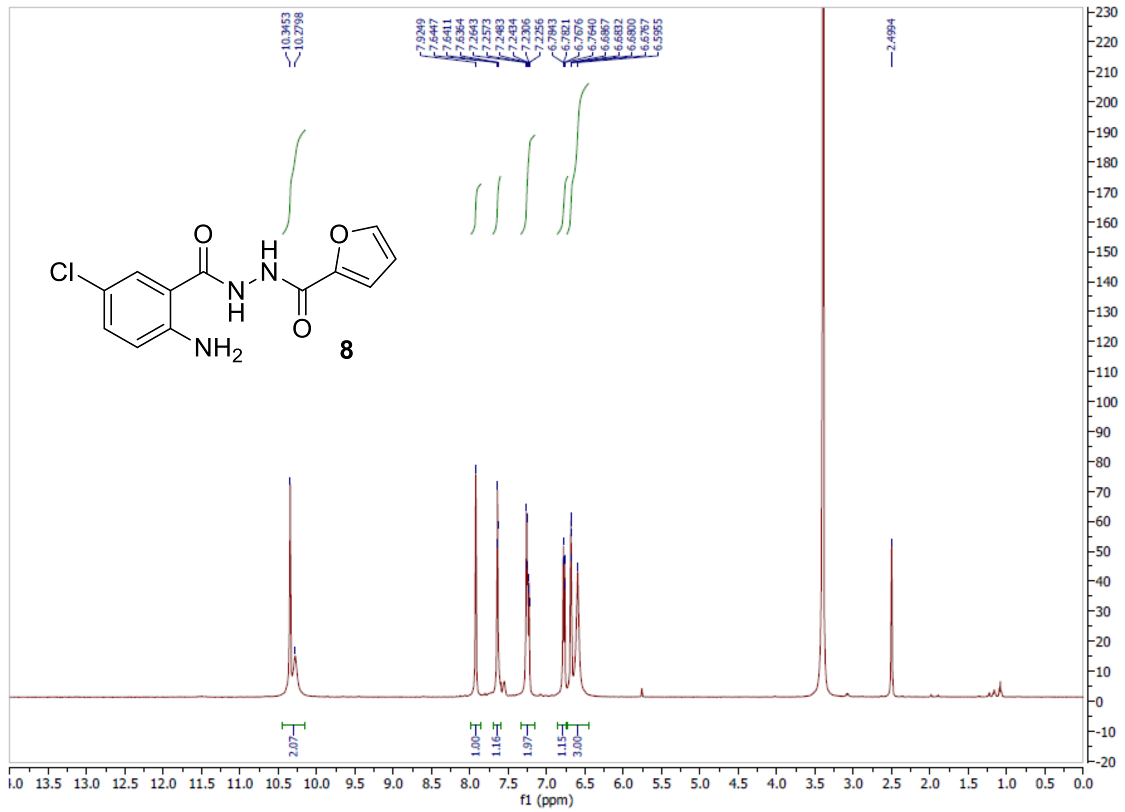

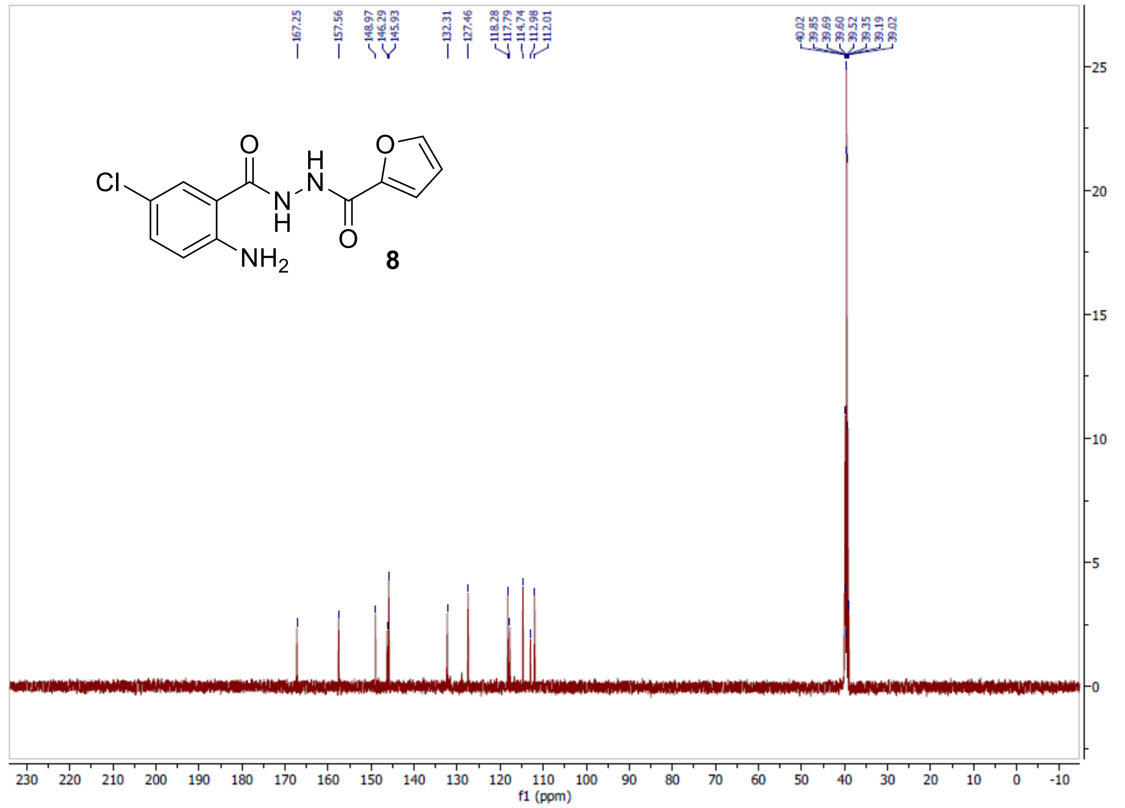

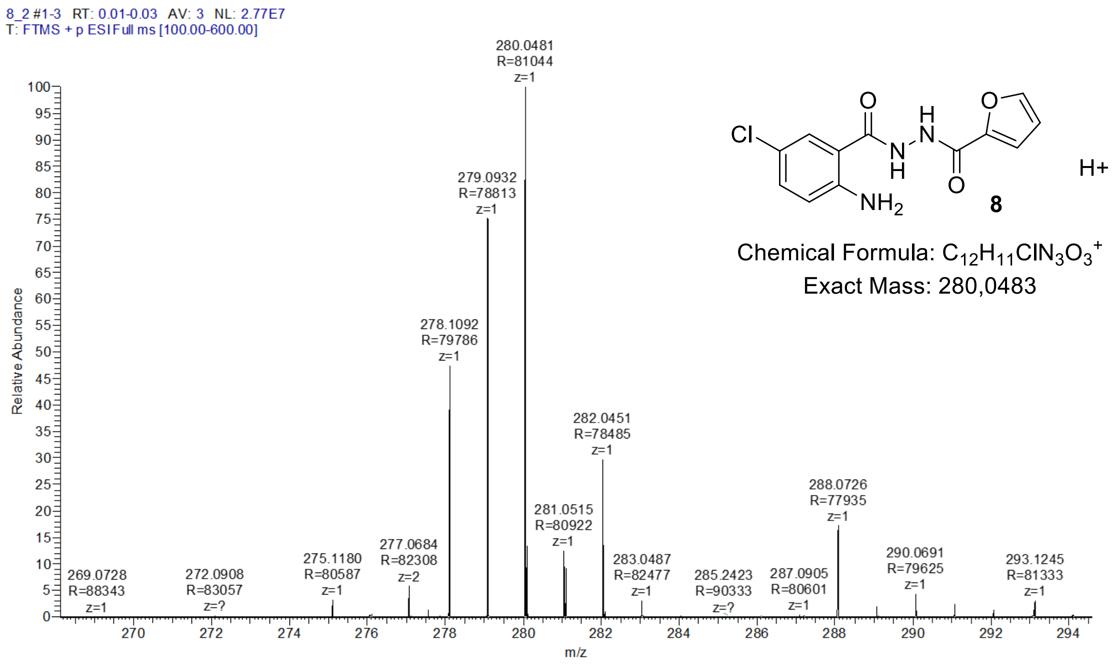
8_2 #1-3 RT: 0.01-0.03 AV: 3 NL: 2.77E7
T: FTMS + p ESI Full ms [100.00-600.00]
280.0481 R=81044 z=1
279.0932 R=78813 z=1
278.1092 R=79786 z=1
282.0451 R=78485 z=1
281.0515 R=80922 z=1
288.0726 R=77935 z=1
277.0684 R=82308 z=2
275.1180 R=80587 z=1
283.0487 R=82477 z=1
290.0691 R=79625 z=1
293.1245 R=81333 z=1
269.0728 R=88343 z=1
272.0908 R=83057 z=?
285.2423 R=90333 z=?
287.0905 R=80601 z=1
Relative Abundance
m/z
Cl
O
H
N
N
H
O
O
NH2
8
H+
Chemical Formula: $C_{12}H_{11}ClN_3O_3^+$
Exact Mass: 280,0483

2-amino-*N′*-benzoyl-5-bromobenzohydrazide (**9**)

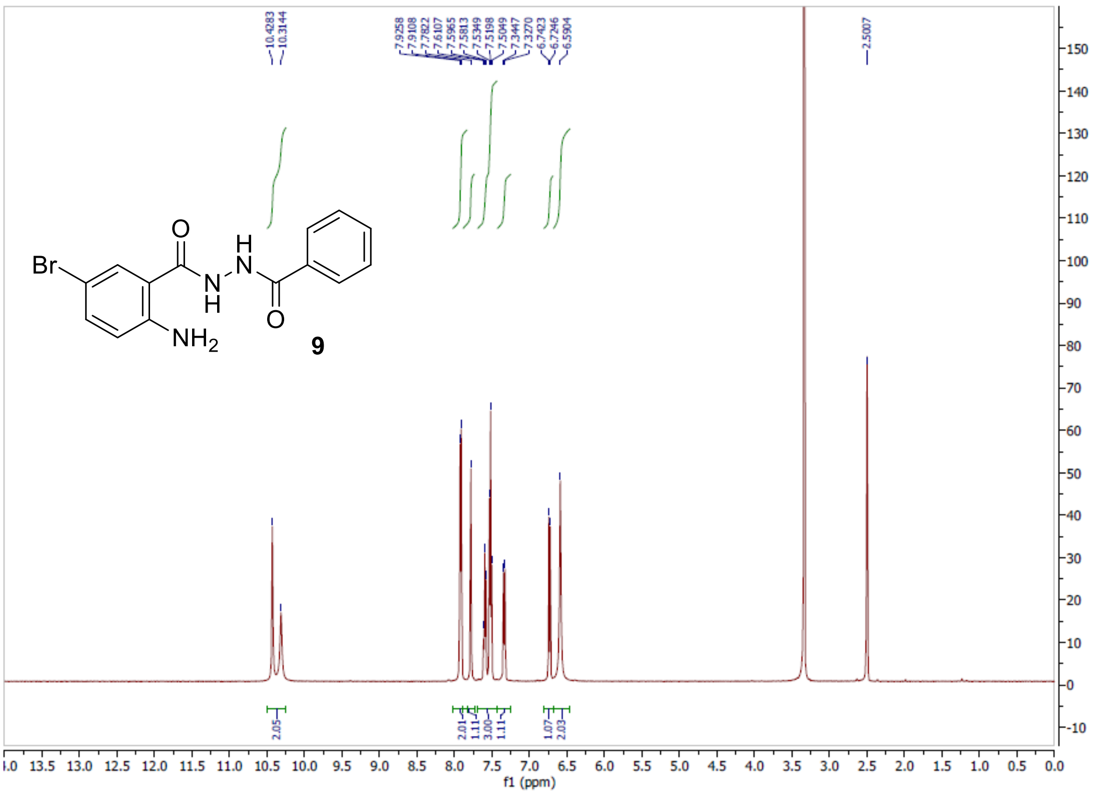

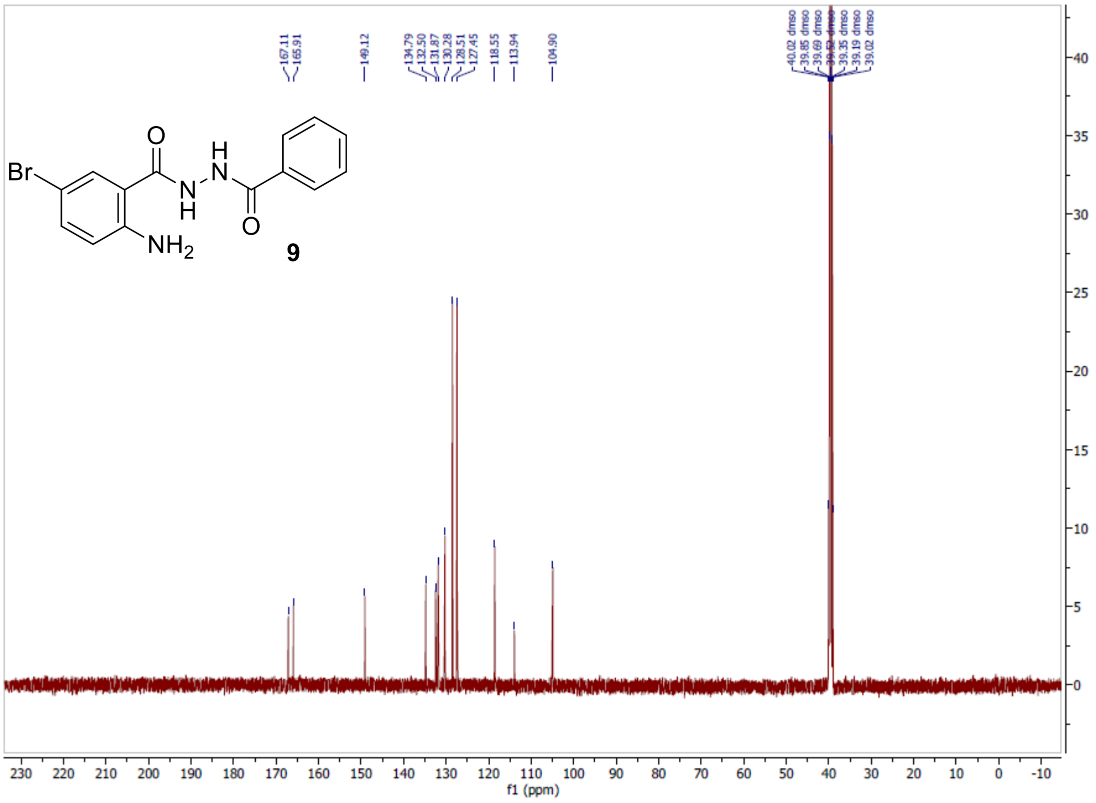

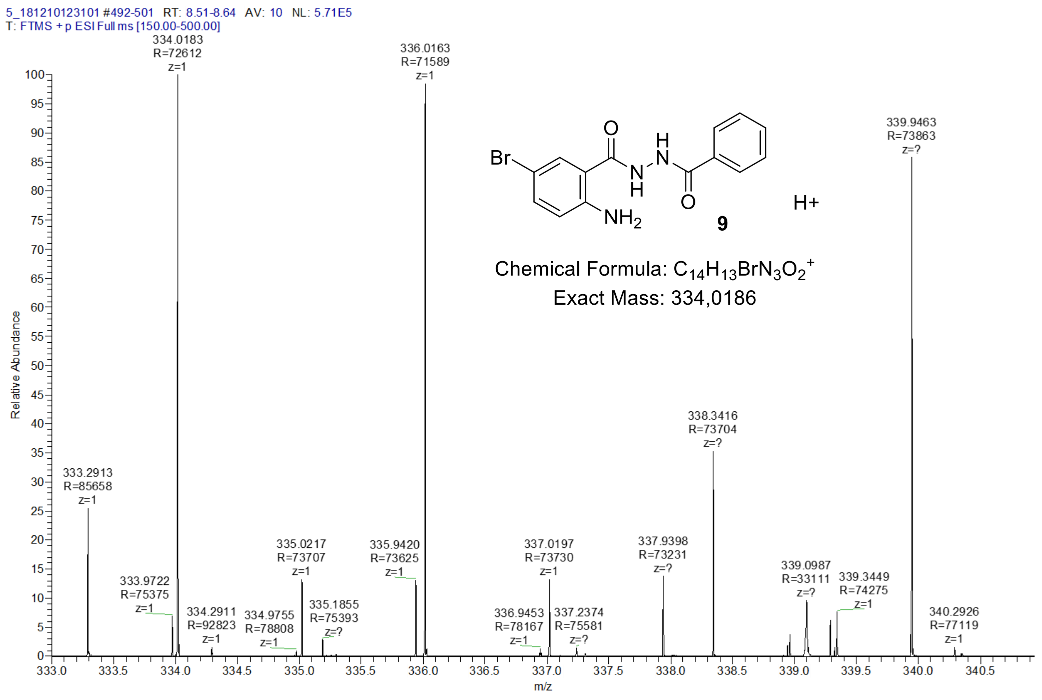

5_181210123101 #492-501 RT: 8.51-8.64 AV: 10 NL: 5.71E5
T: FTMS + p ESI Full ms [150.00-500.00]
Relative Abundance
m/z
334.0183 R=72612 z=1
336.0163 R=71589 z=1
339.9463 R=73863 z=?
338.3416 R=73704 z=?
333.2913 R=85658 z=1
337.9398 R=73231 z=?
335.0217 R=73707 z=1
337.0197 R=73730 z=1
335.9420 R=73625 z=1
339.0987 R=33111 z=?
339.3449 R=74275 z=1
333.9722 R=75375 z=1
334.2911 R=92823 z=1
334.9755 R=78808 z=1
335.1855 R=75393 z=?
336.9453 R=78167 z=1
337.2374 R=75581 z=?
340.2926 R=77119 z=1
Br
O
H
N
NH2
9
H+
Chemical Formula: C14H13BrN3O2+
Exact Mass: 334,0186

2-amino-5-bromo-*N′*-(4-chlorobenzoyl)benzohydrazide (**10**)

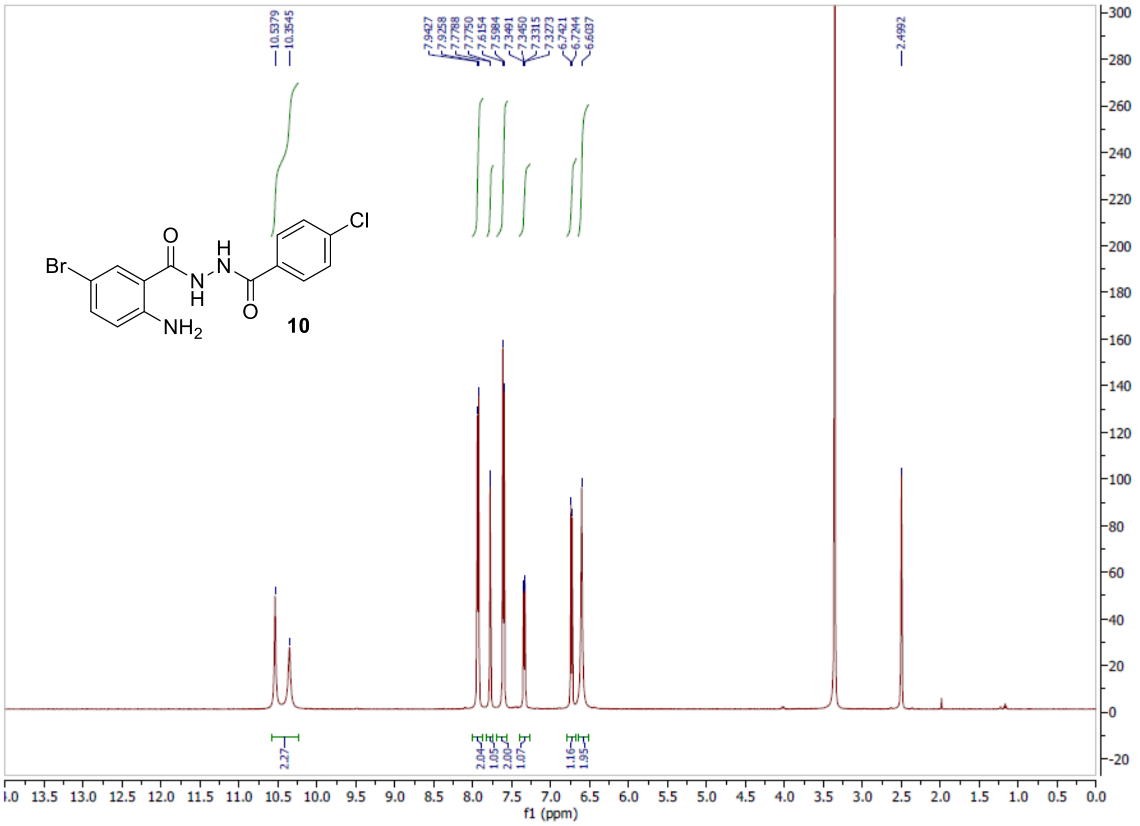

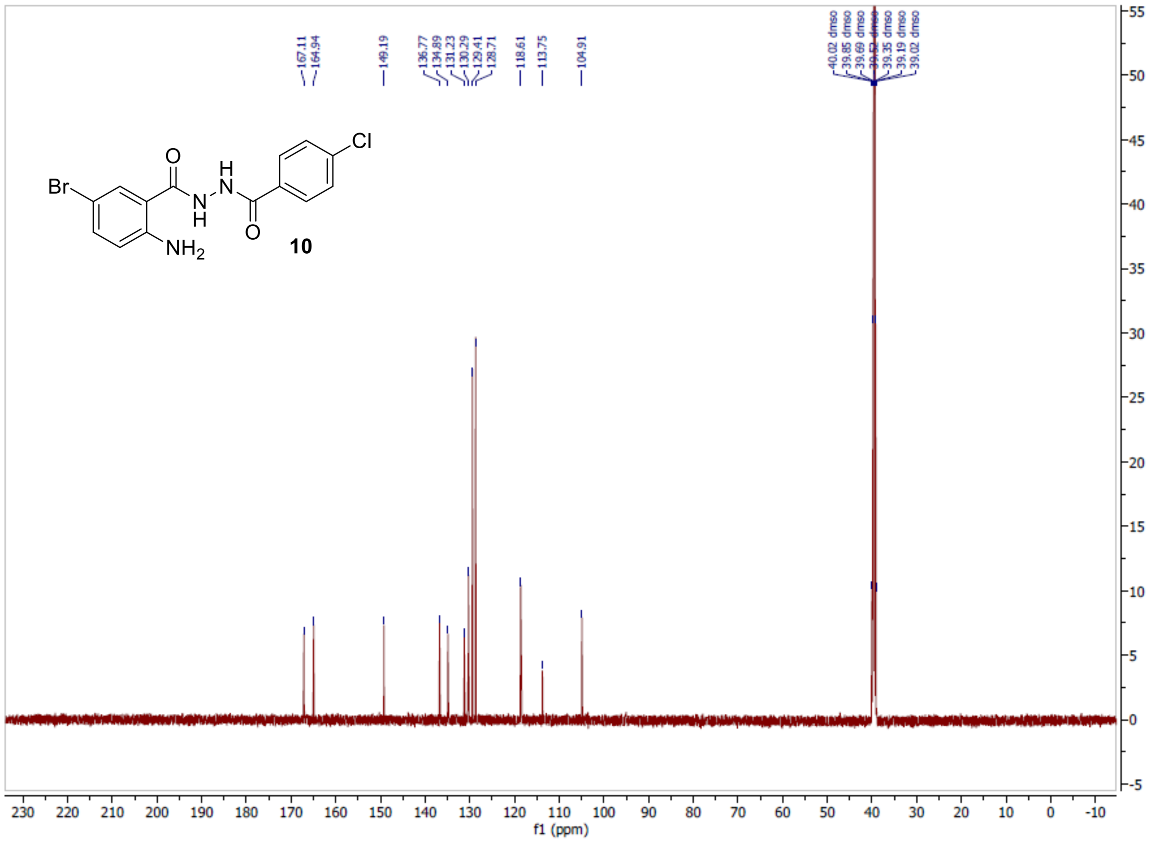

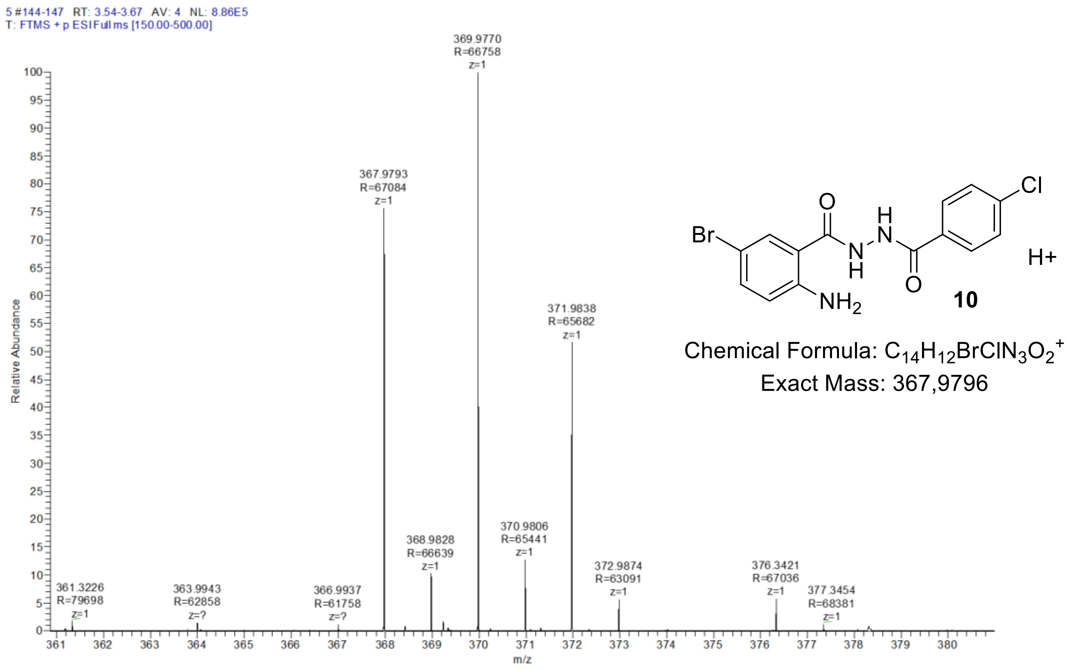

10

Chemical Formula: $C_{14}H_{12}BrClN_3O_2^+$
Exact Mass: 367,9796

2-amino-5-bromo-*N′*-(4-nitrobenzoyl)benzohydrazide (**11**)

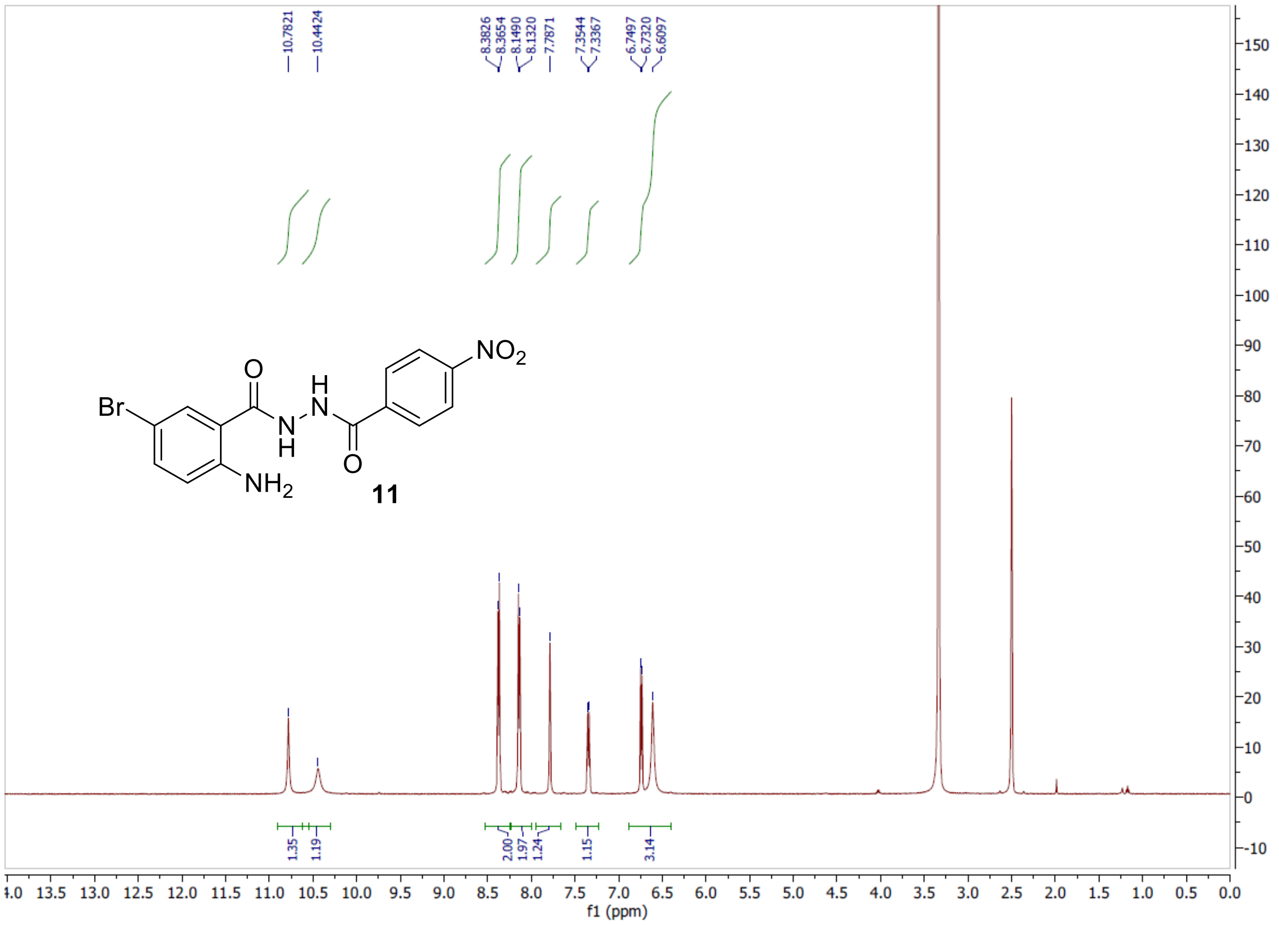

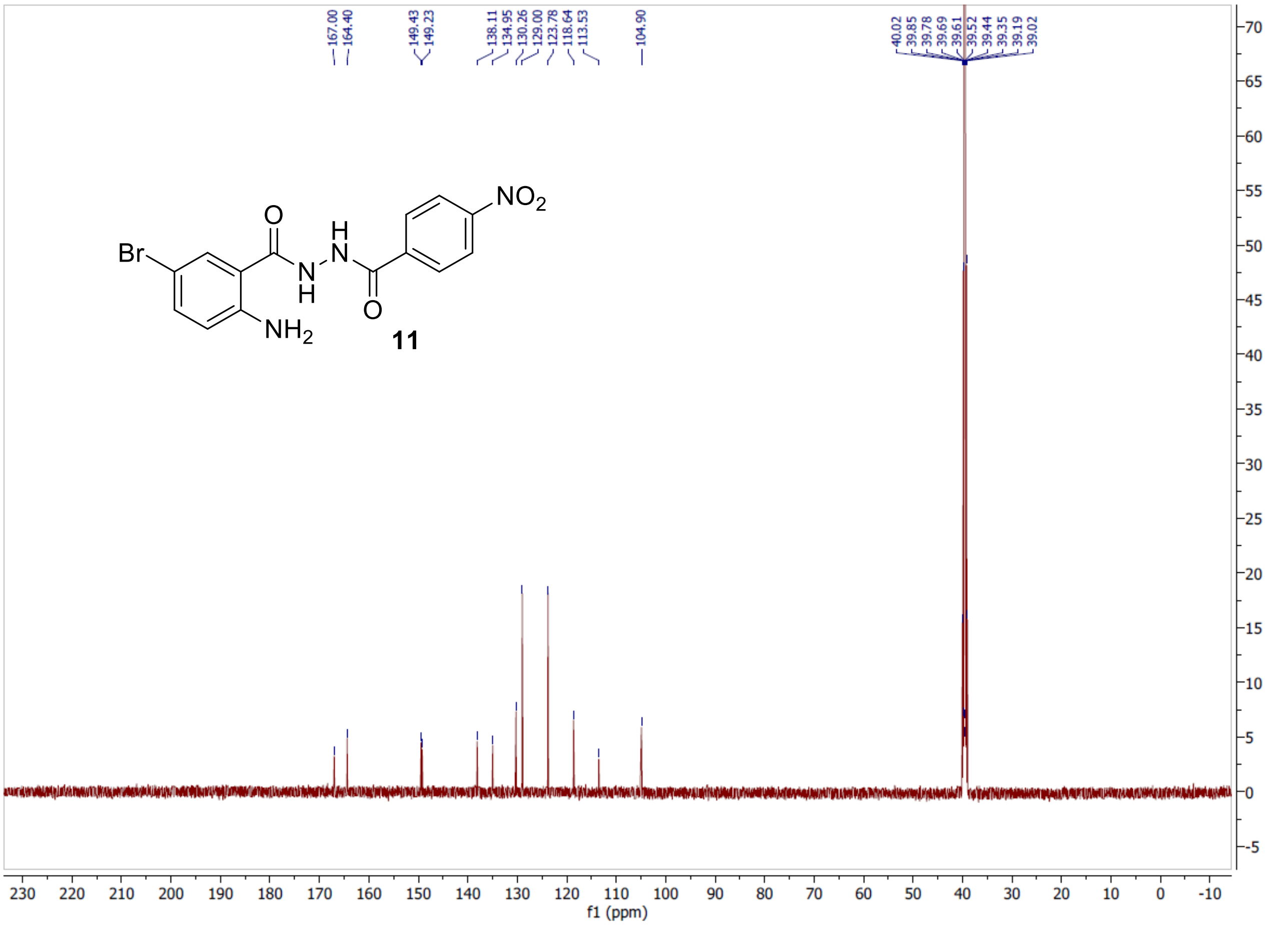

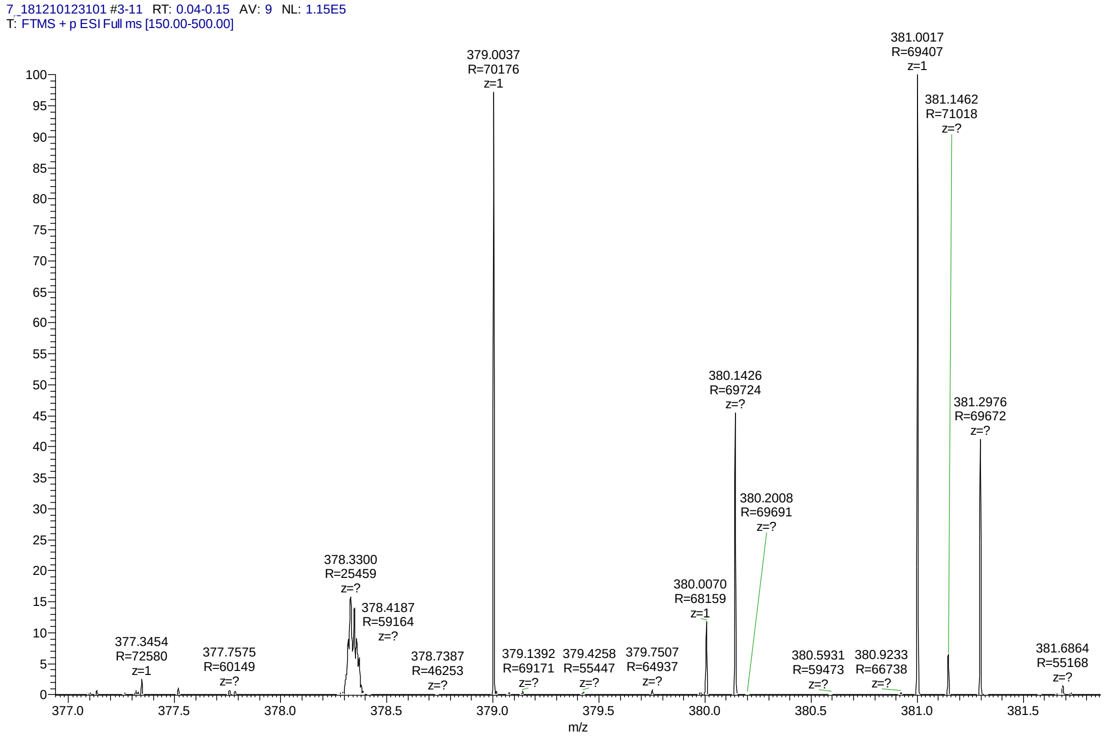

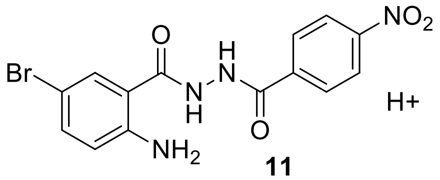

11

Chemical Formula: $C_{14}H_{12}BrN_4O_4^+$

Exact Mass: 379,0036

Molecular Weight: 380,1729

N′-(2-amino-5-bromobenzoyl)furan-2-carbohydrazide (**12**)

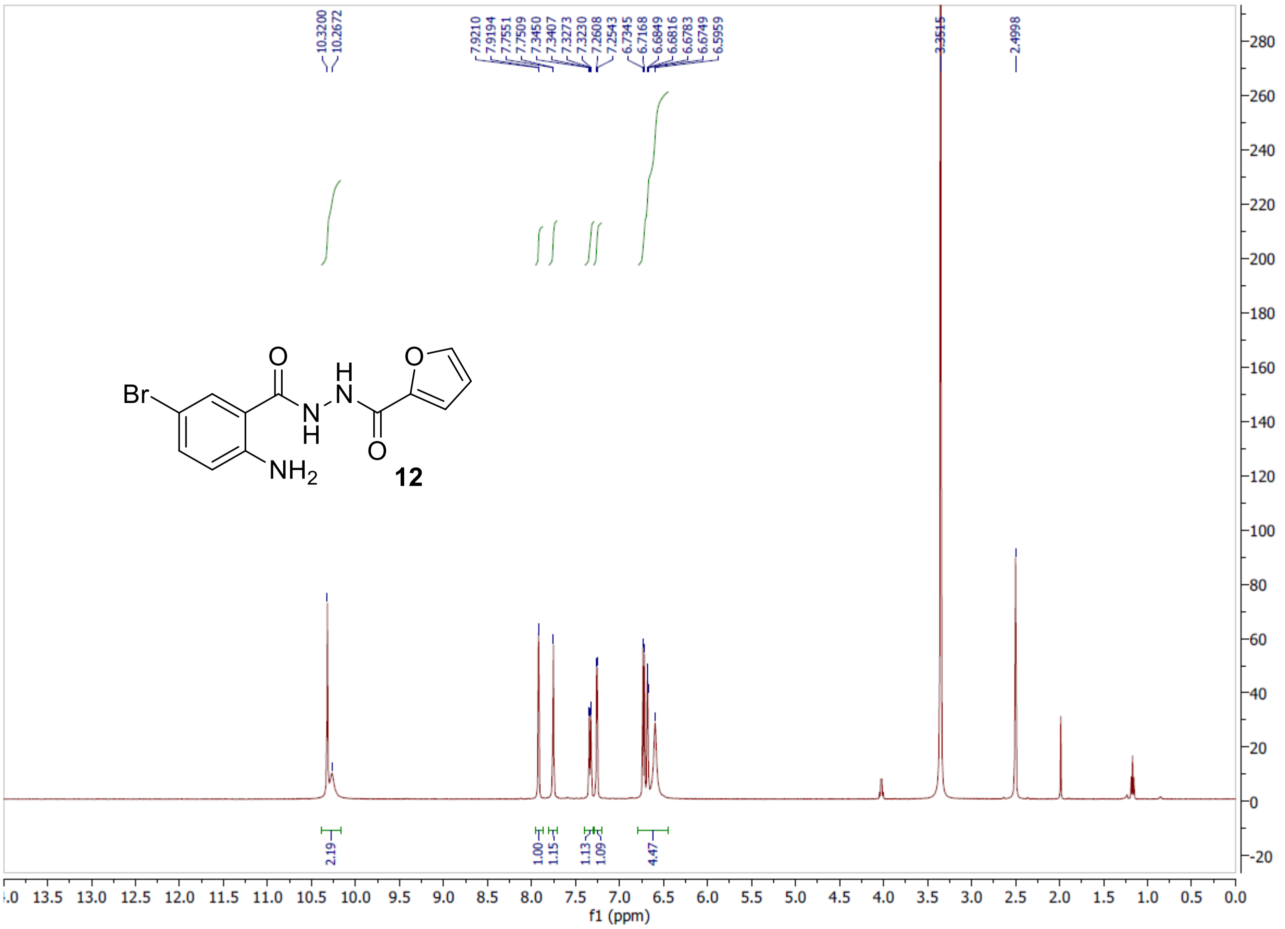

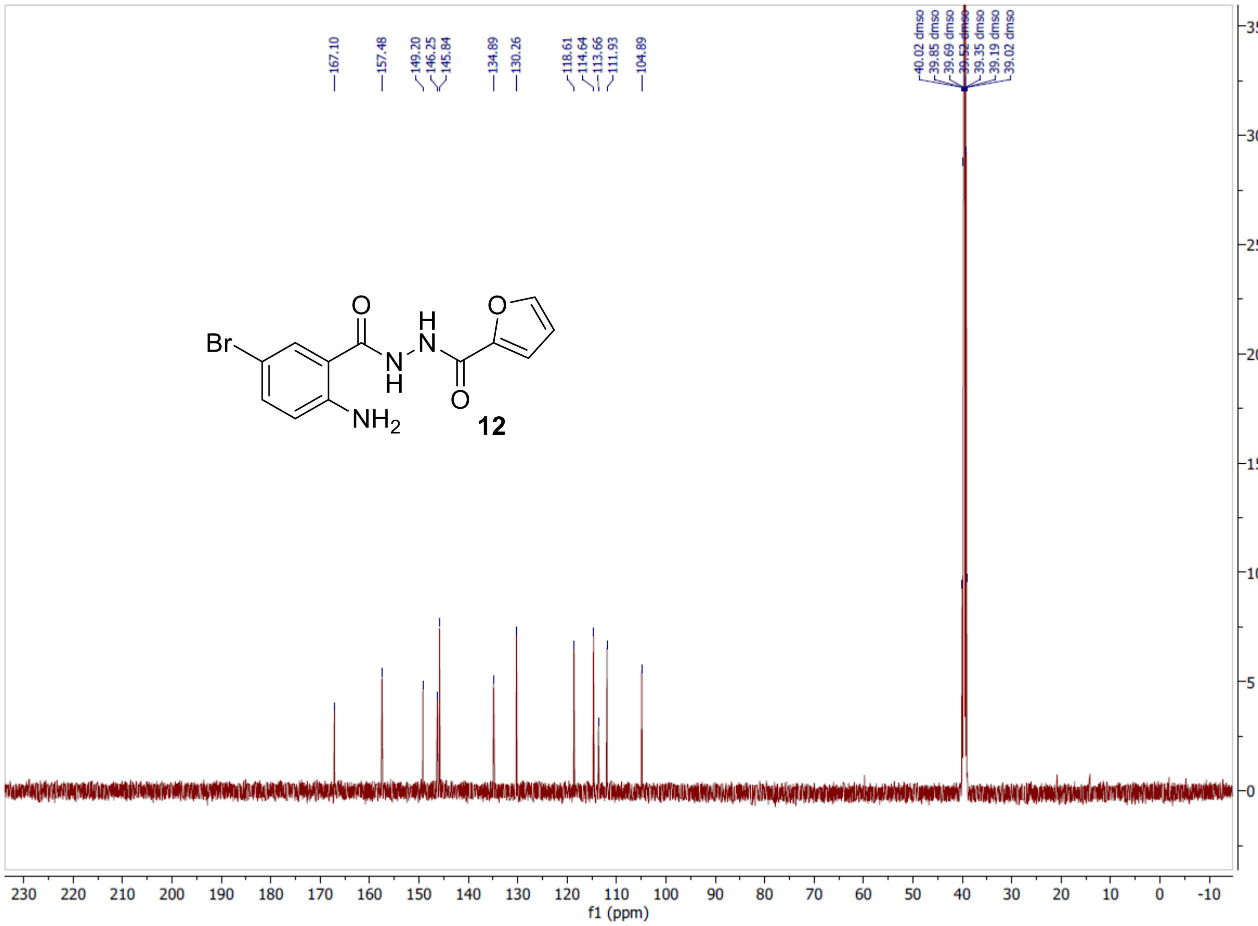

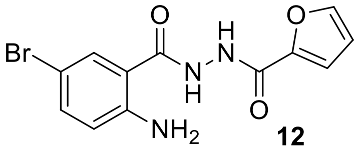

12 H+

Chemical Formula: $C_{12}H_{11}BrN_3O_3^+$

Exact Mass: 323,9978

Molecular Weight: 325,1375

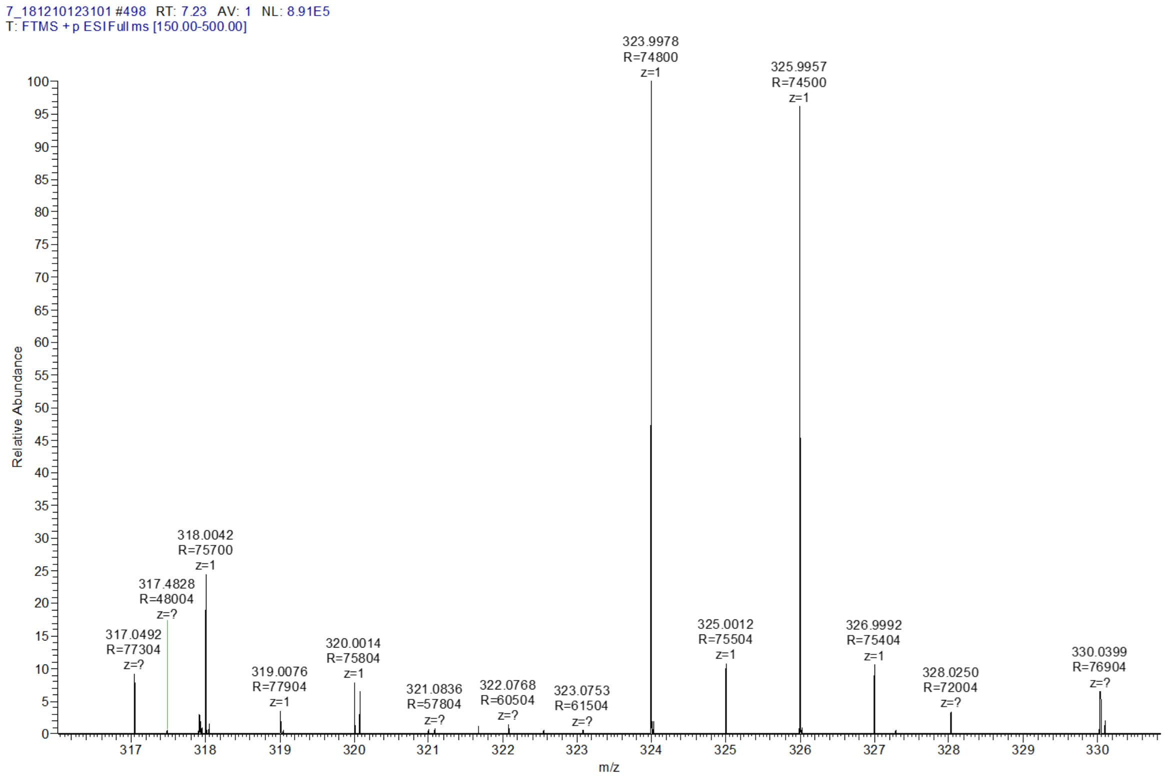

2-amino-*N′*-benzoyl-3,5-dibromobenzohydrazide (**13**)

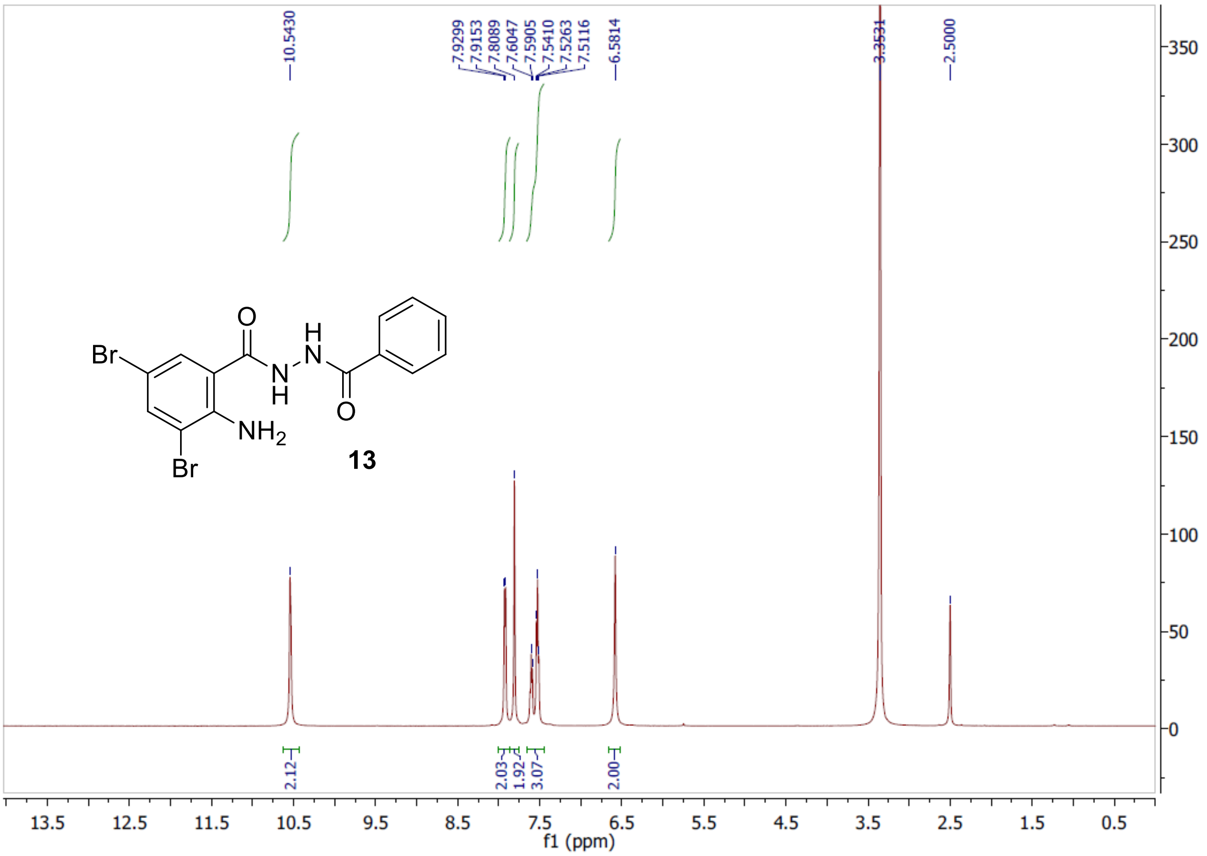

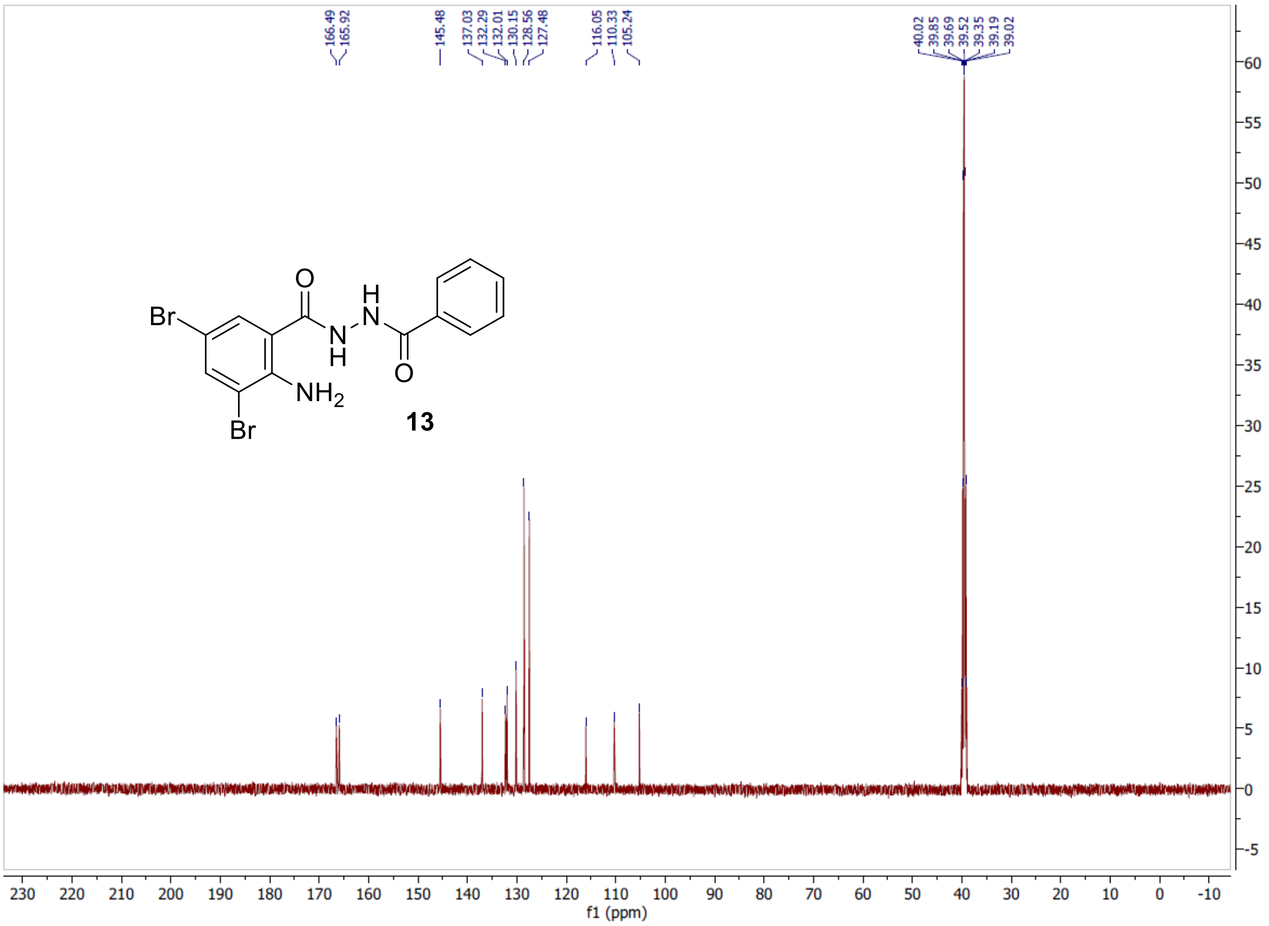

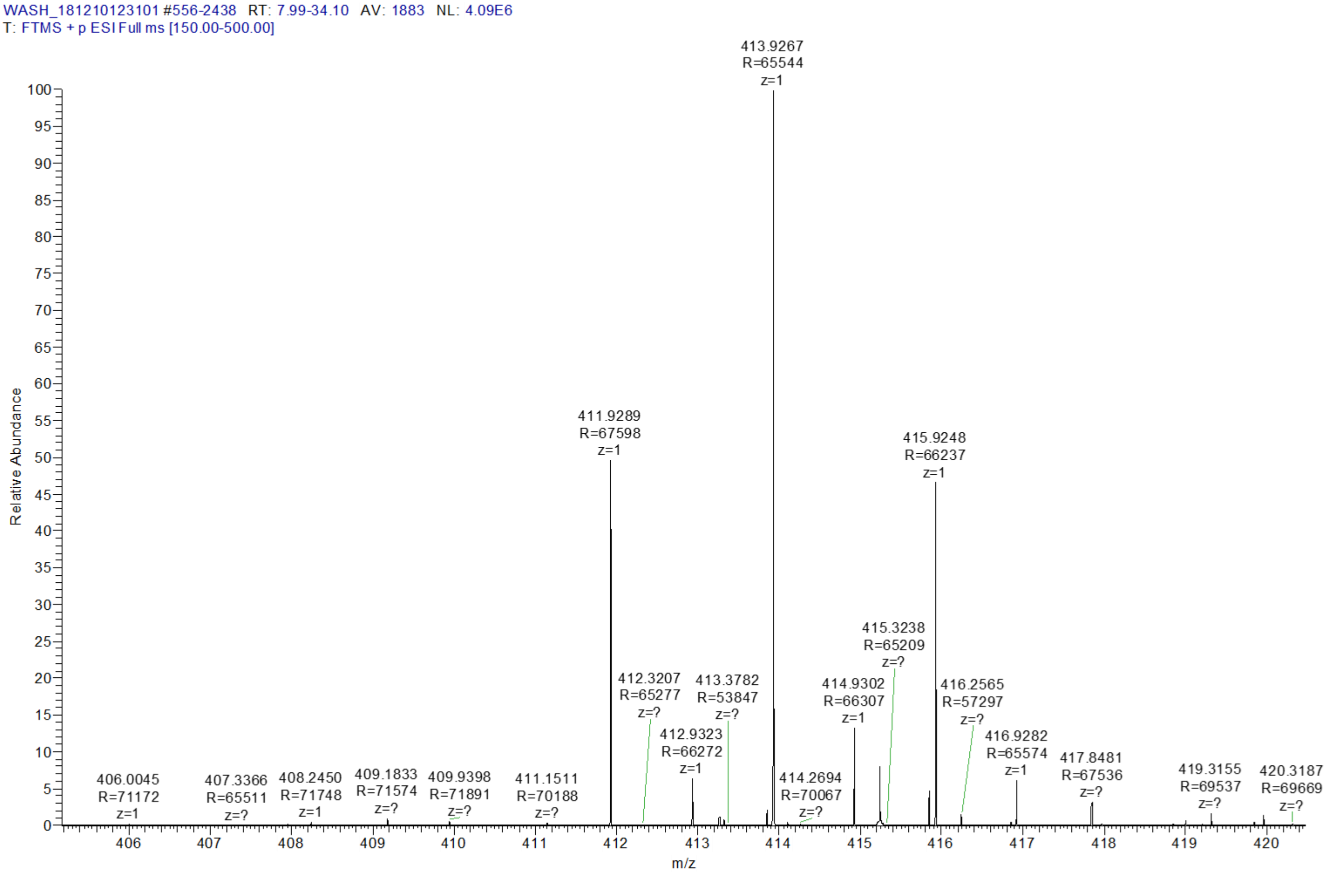

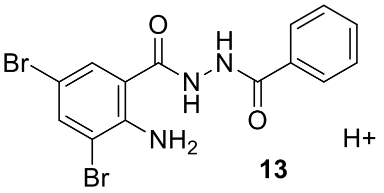

13

Chemical Formula: $C_{14}H_{12}Br_2N_3O_2^+$
Exact Mass: 411,9291
Molecular Weight: 414,0714

2-amino-3,5-dibromo-*N′*-(4-chlorobenzoyl)benzohydrazide (**14**)

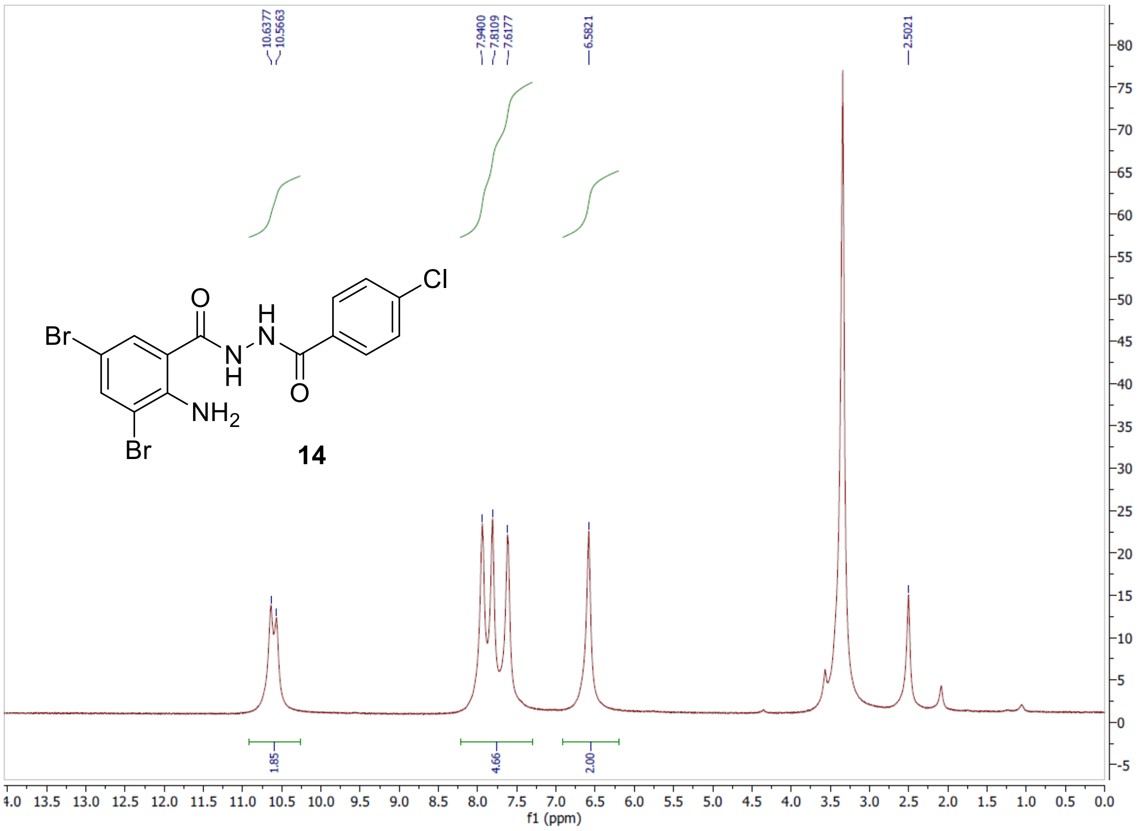

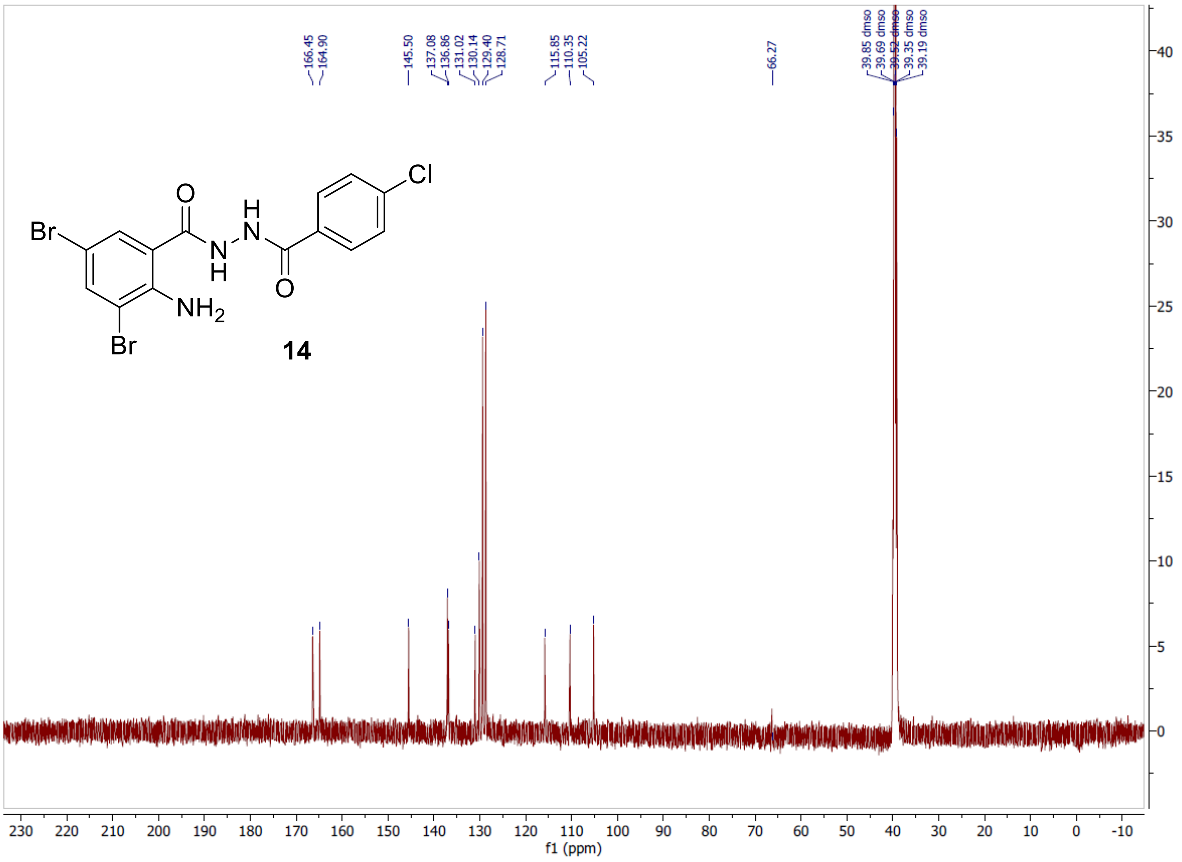

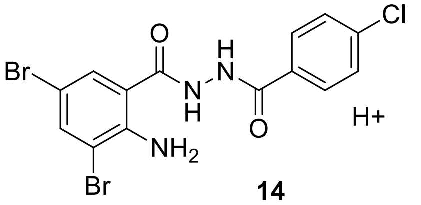

14

Chemical Formula: $C_{14}H_{11}Br_2ClN_3O_2^+$
Exact Mass: 445,8901
Molecular Weight: 448,5165

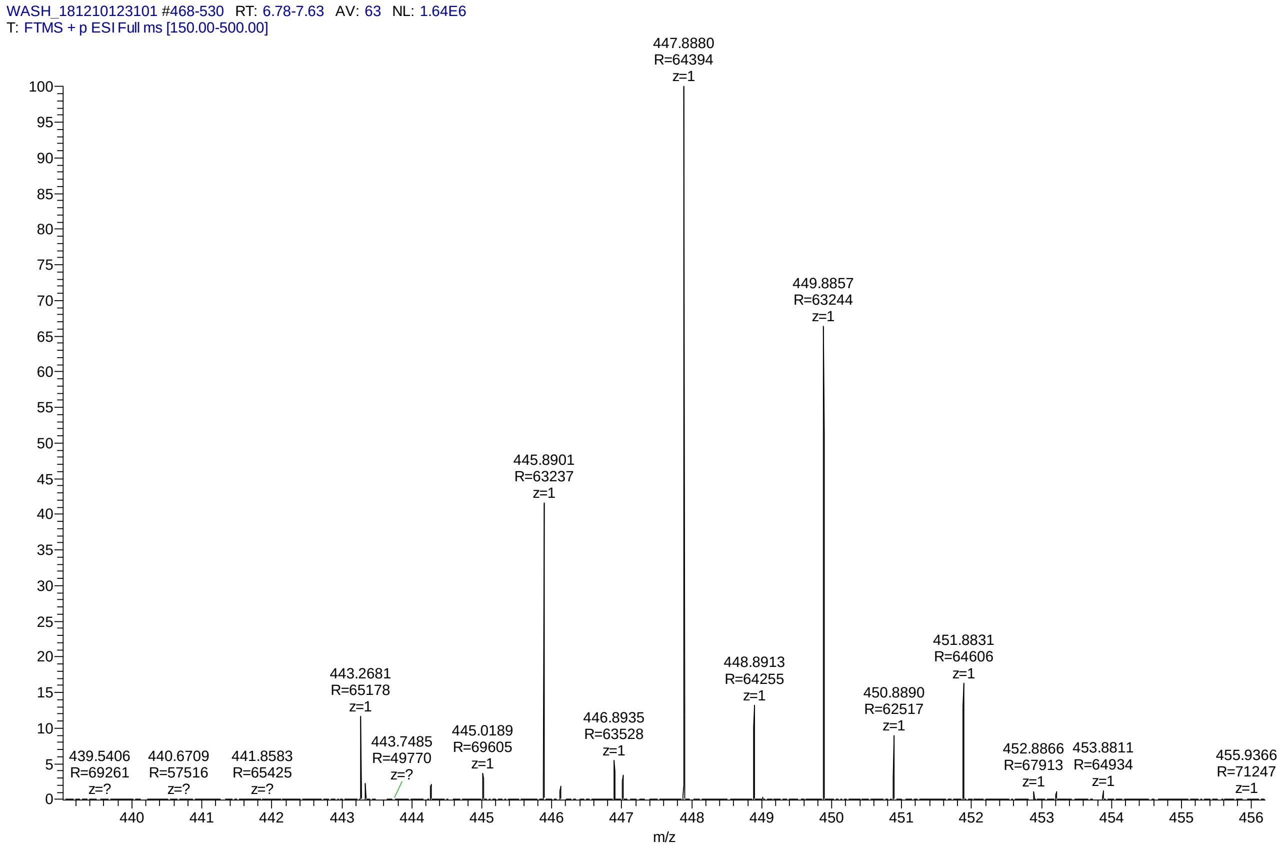

2-amino-3,5-dibromo-*N′*-(4-nitrobenzoyl)benzohydrazide (**15**)

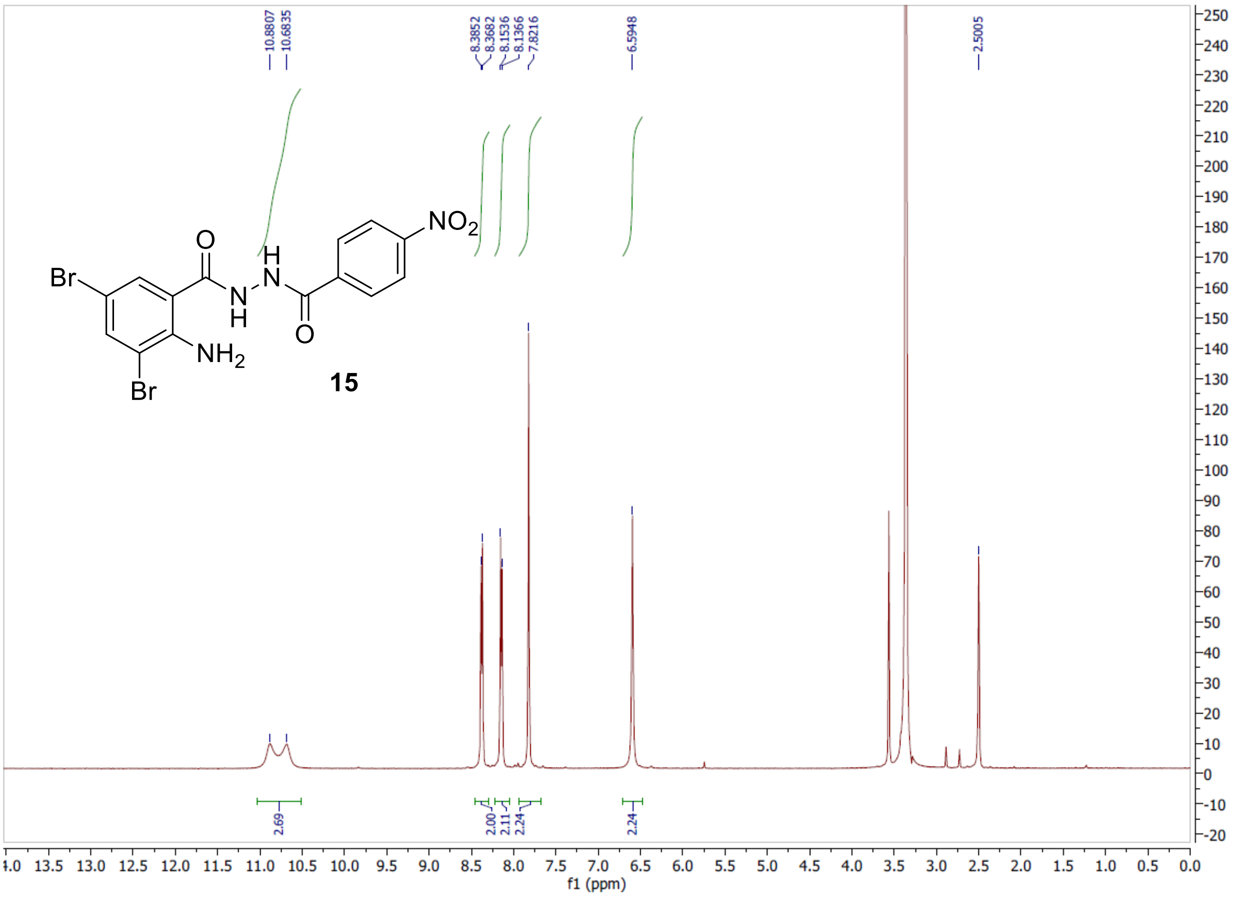

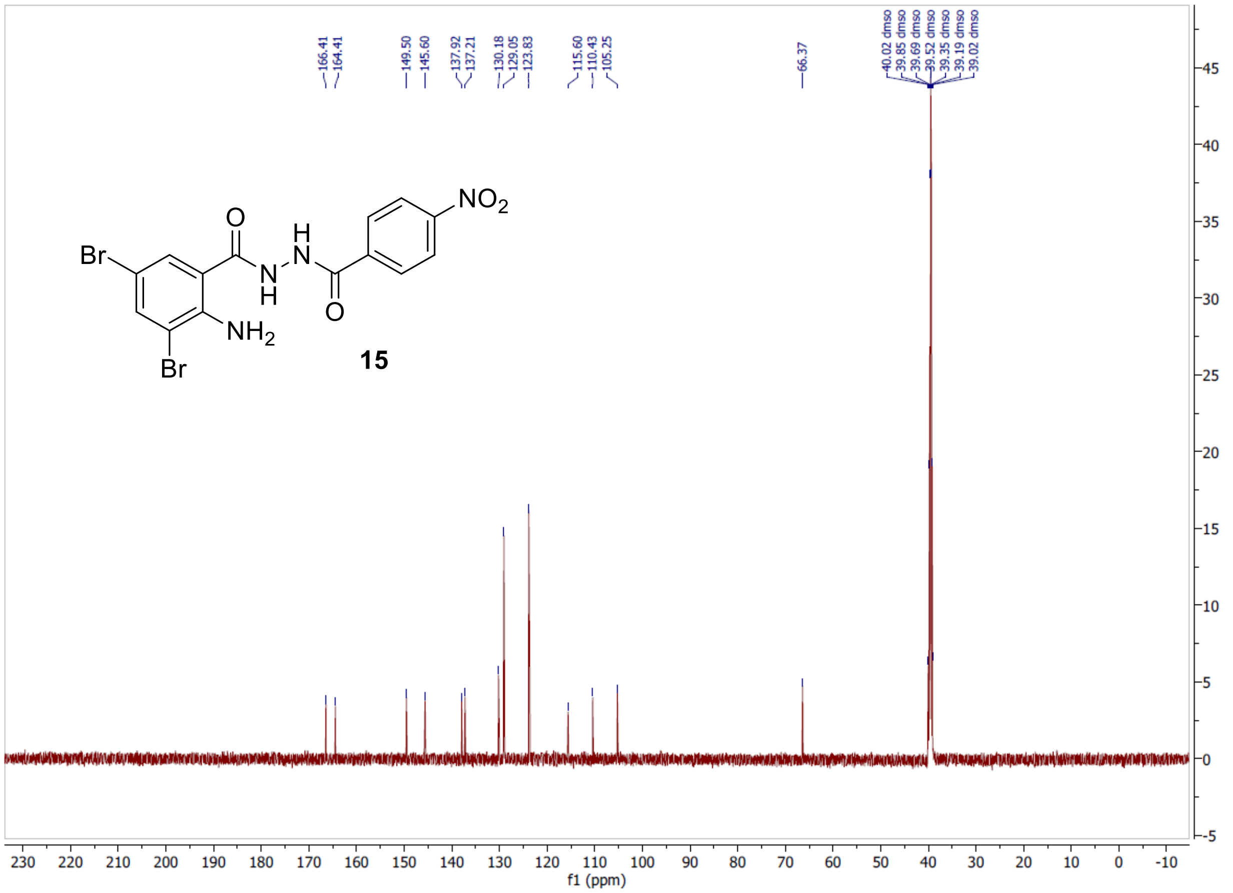

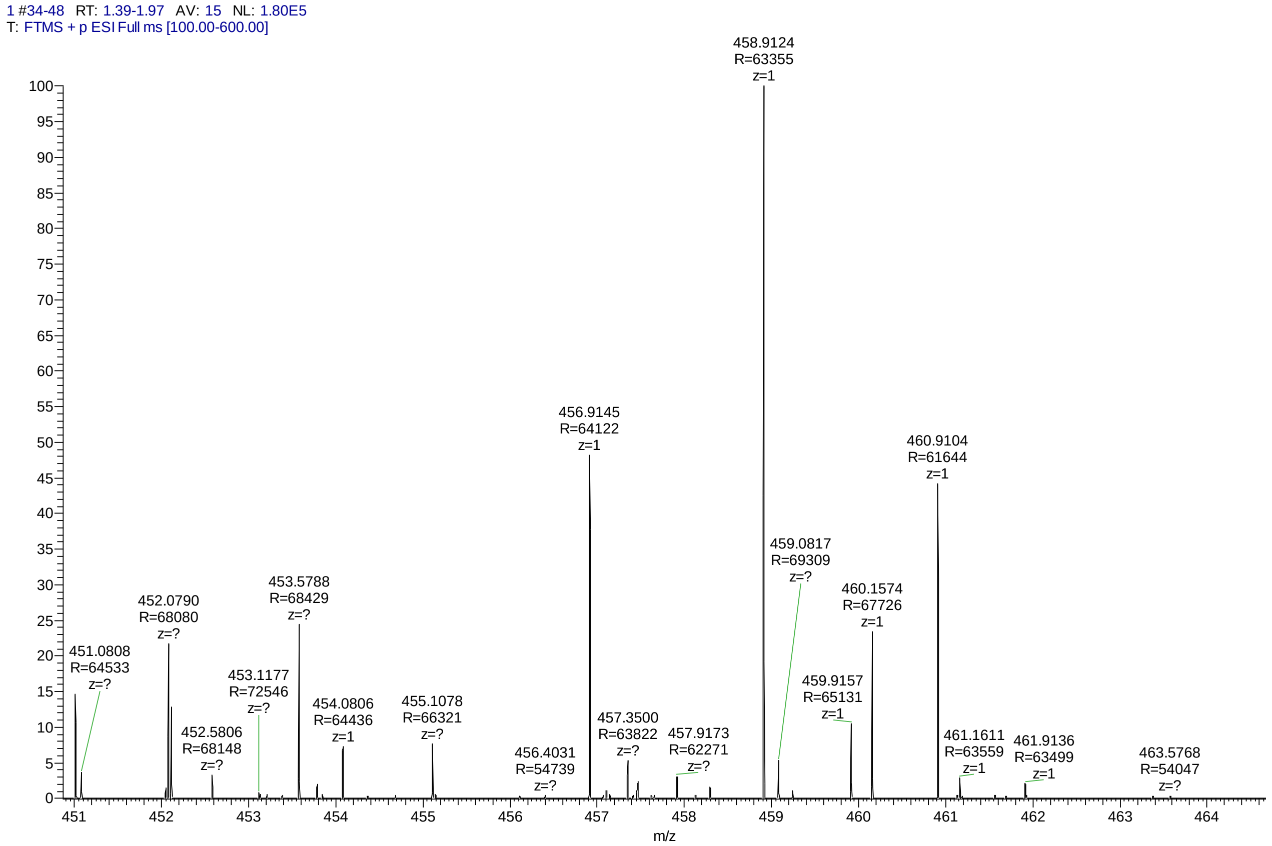

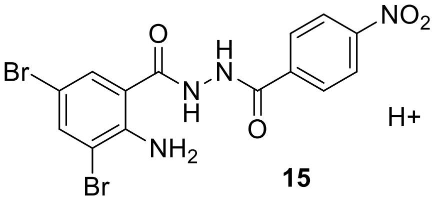

15

Chemical Formula: $C_{14}H_{11}Br_2N_4O_4^+$

Exact Mass: 456,9142

Molecular Weight: 459,0690

N′-(2-amino-3,5-dibromobenzoyl)furan-2-carbohydrazide (**16**)

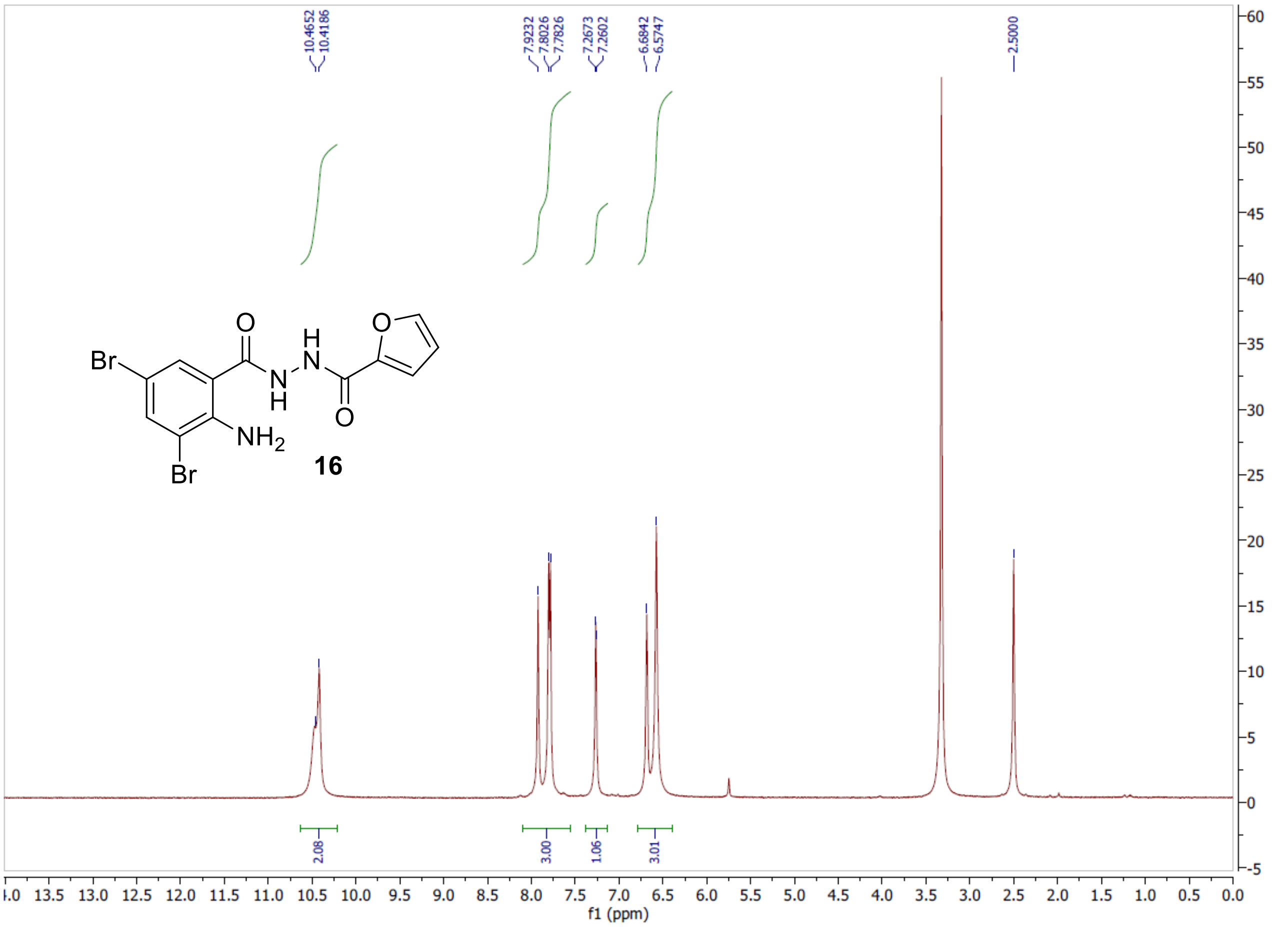

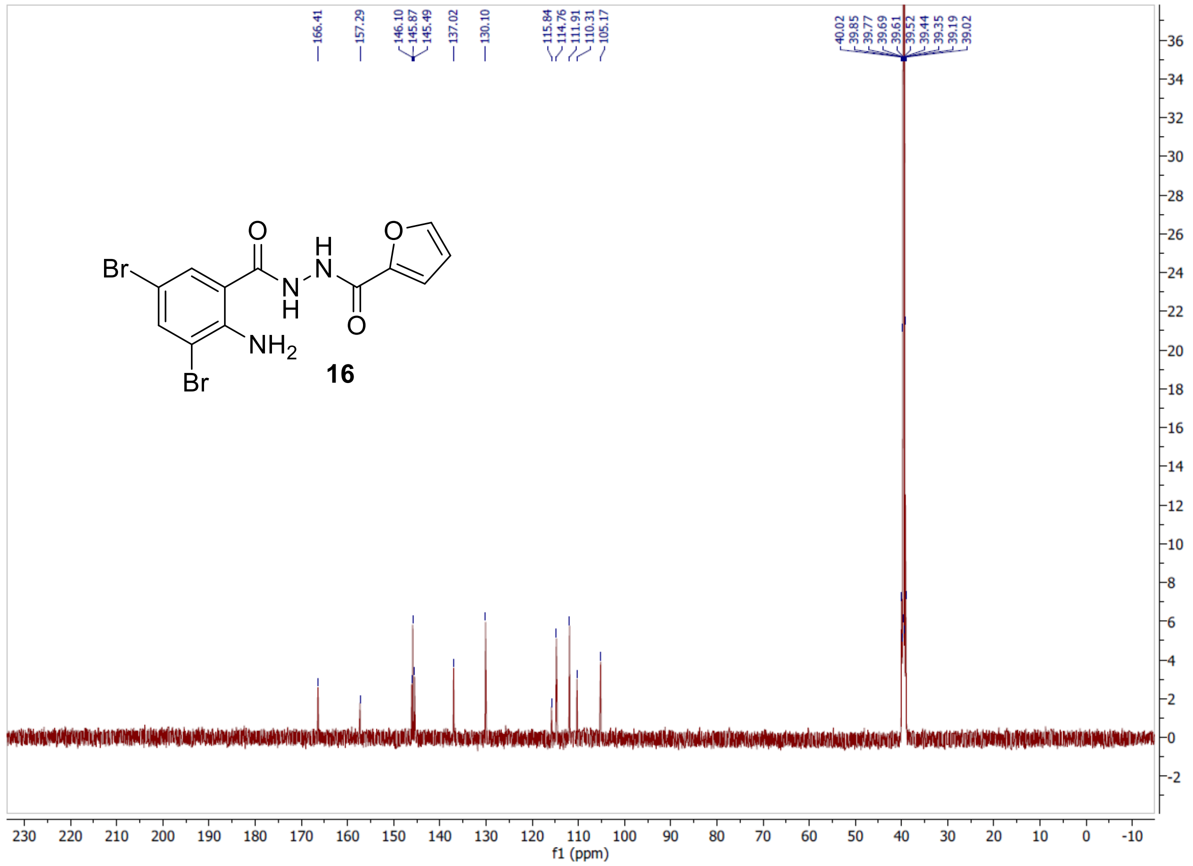

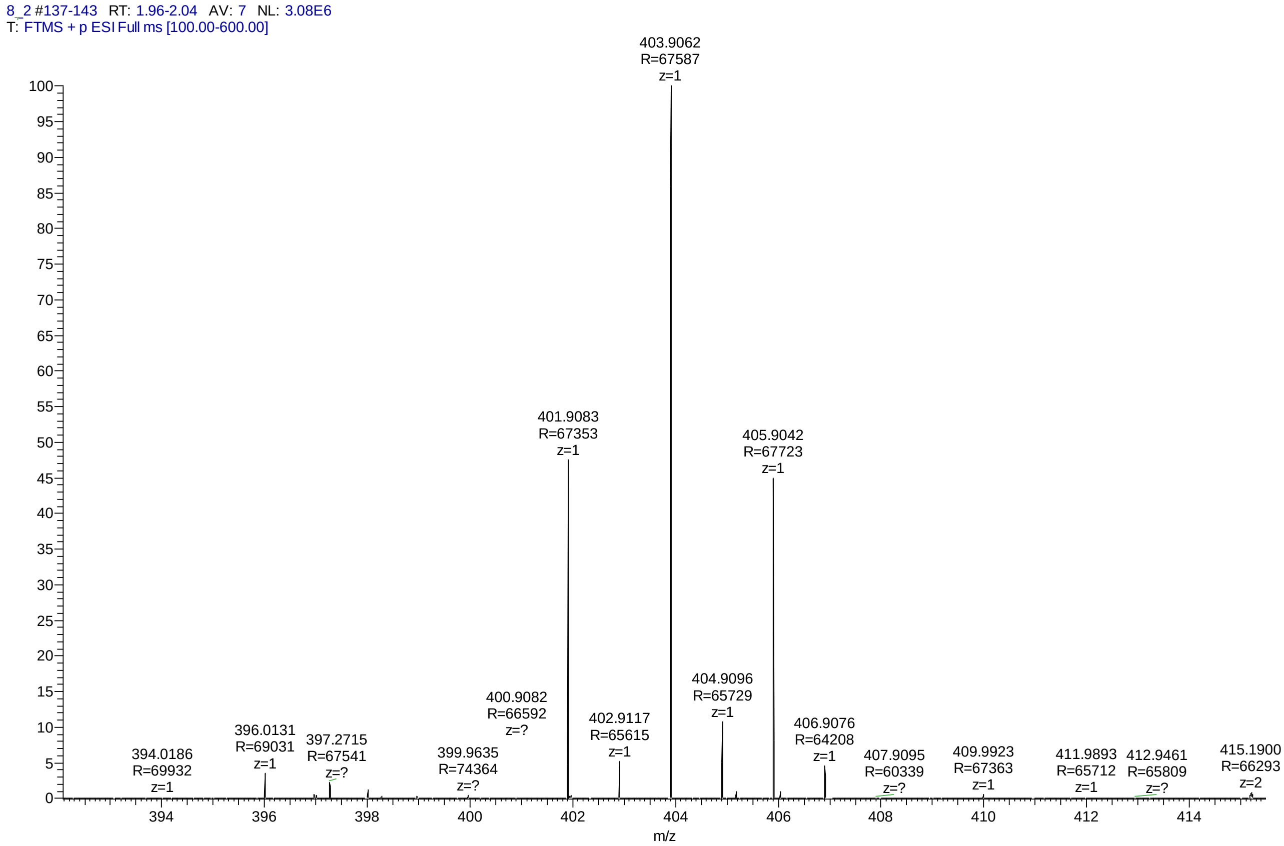

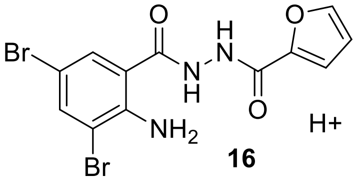

16

Chemical Formula: $C_{12}H_{10}Br_2N_3O_3^+$
Exact Mass: 401,9083
Molecular Weight: 404,0336

2-amino-*N′*-benzoyl-5-iodobenzohydrazide (**17**)

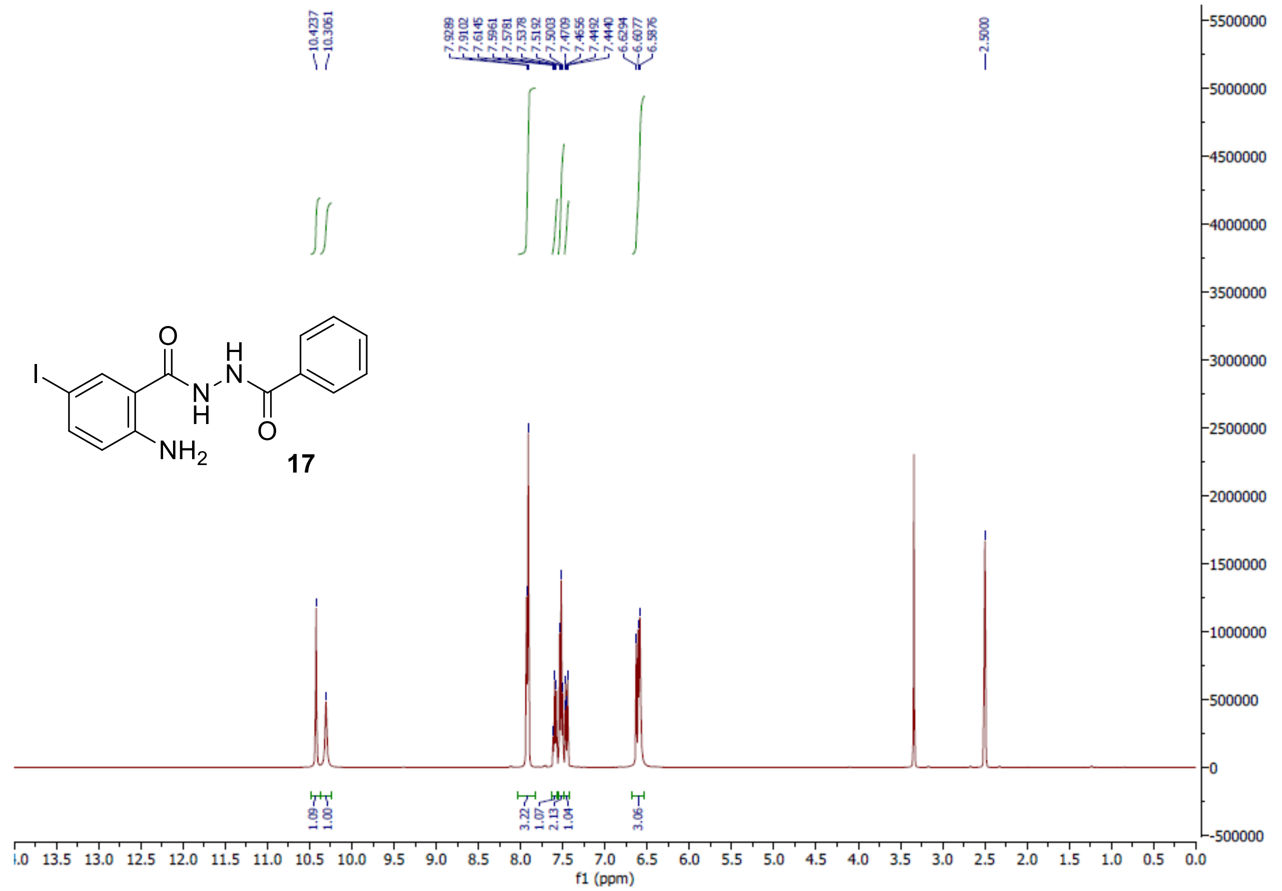

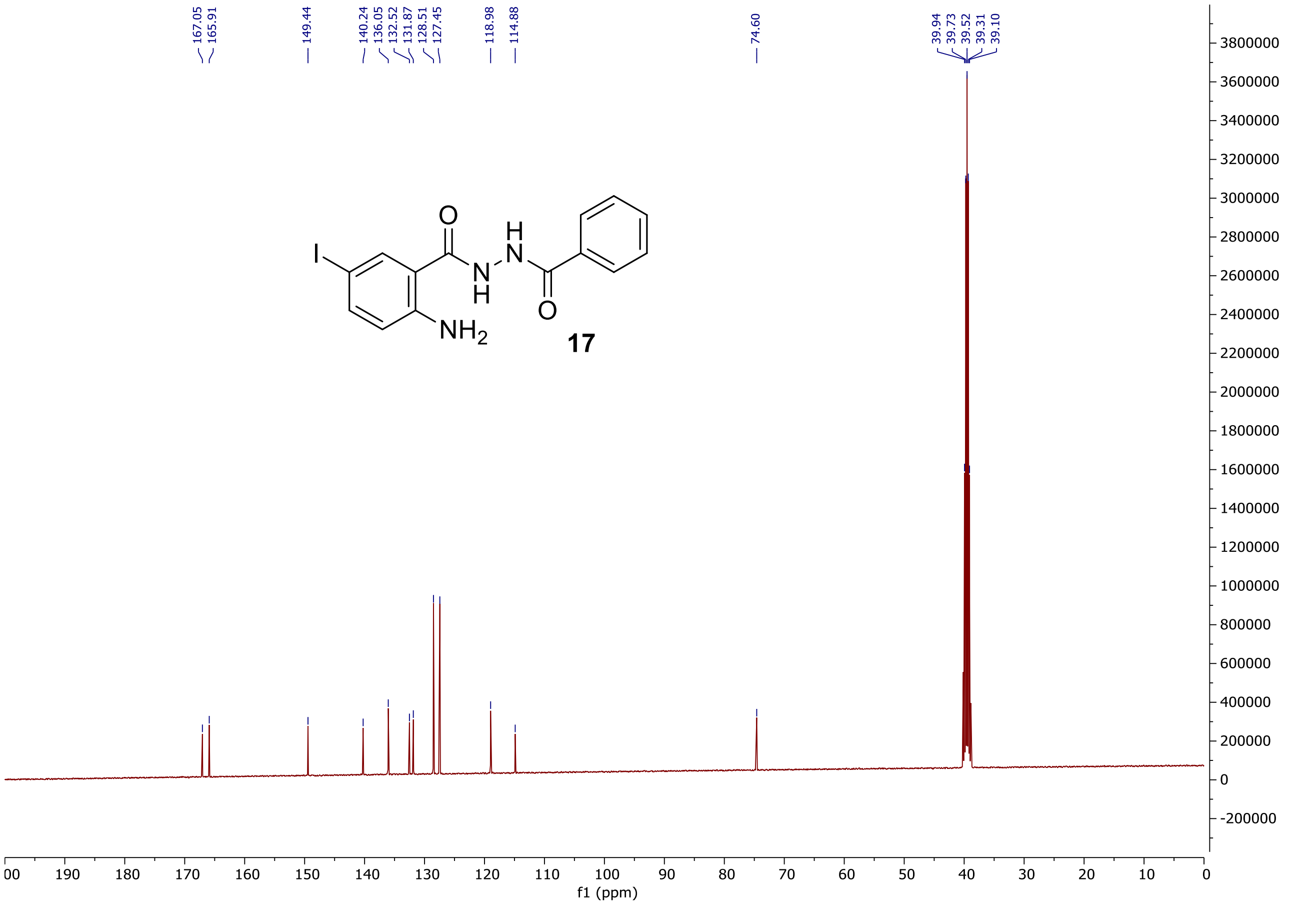

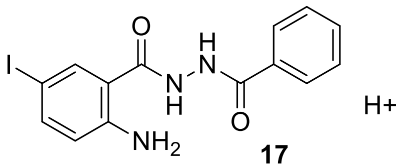

17

Chemical Formula: $C_{14}H_{13}IN_3O_2^+$

Exact Mass: 382,0047

Molecular Weight: 382,1758

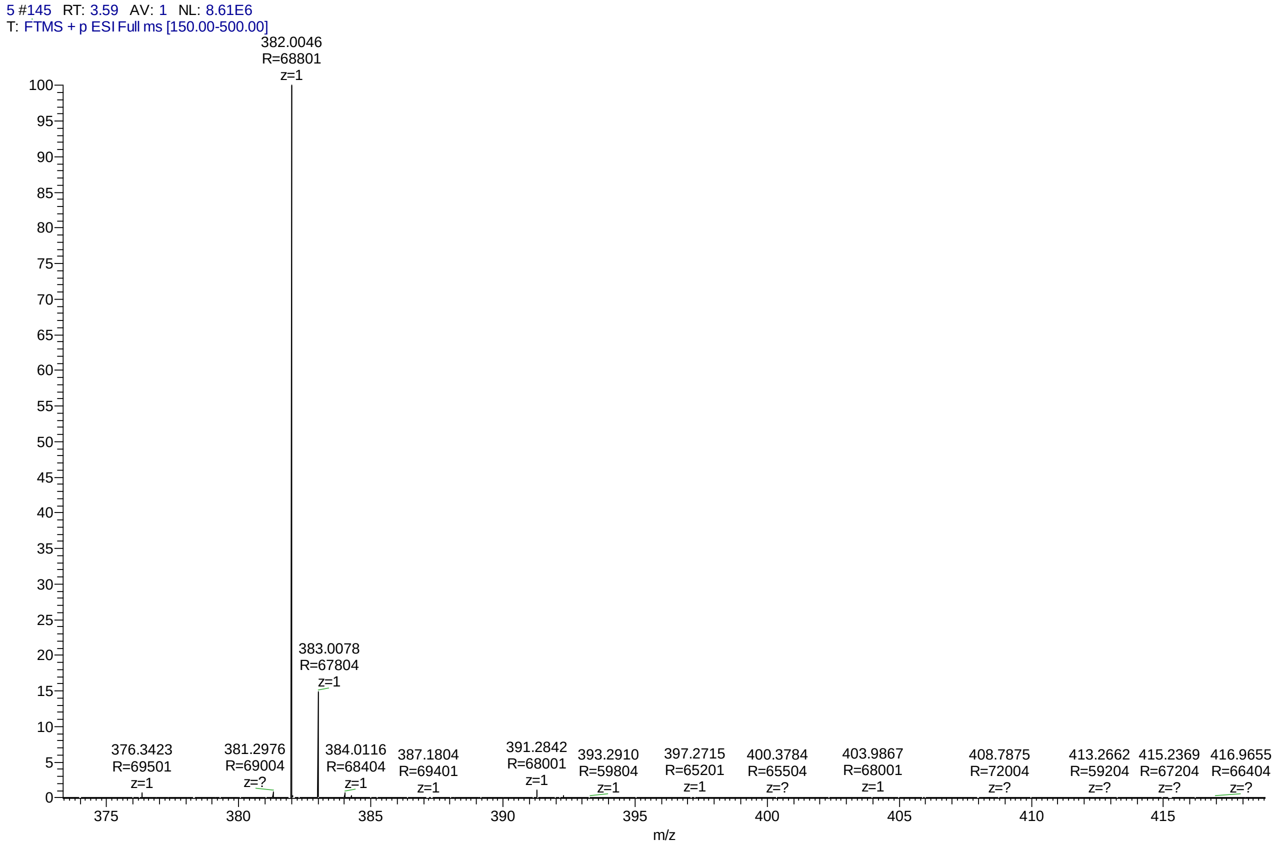

N′-(2-amino-5-iodobenzoyl)furan-2-carbohydrazide (**18**)

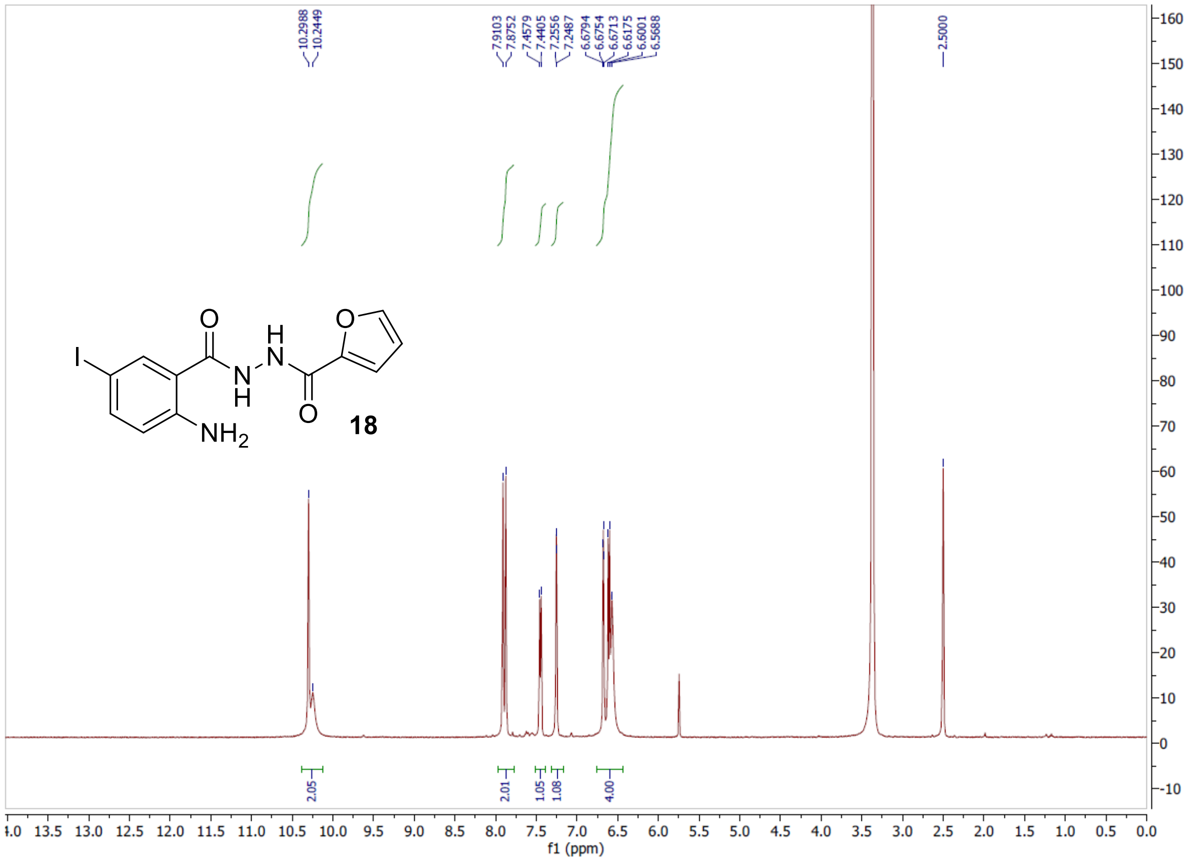

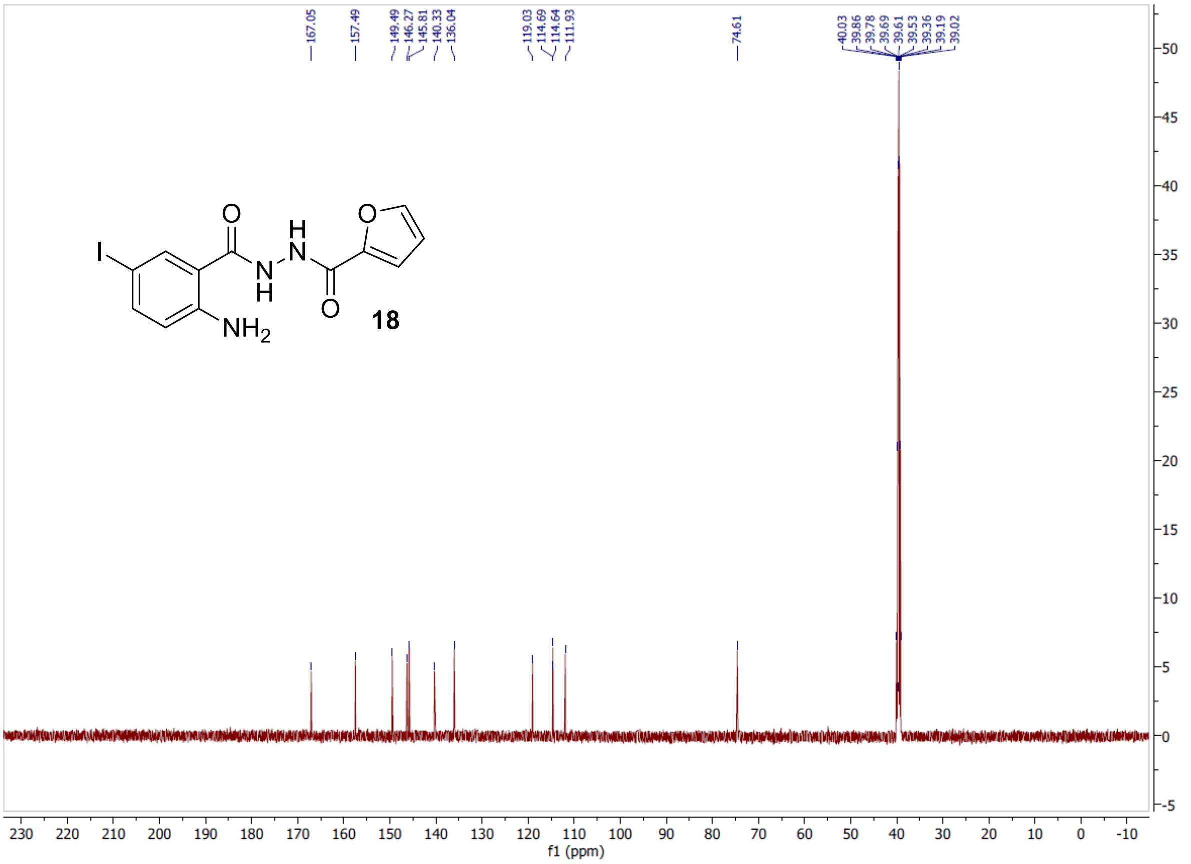

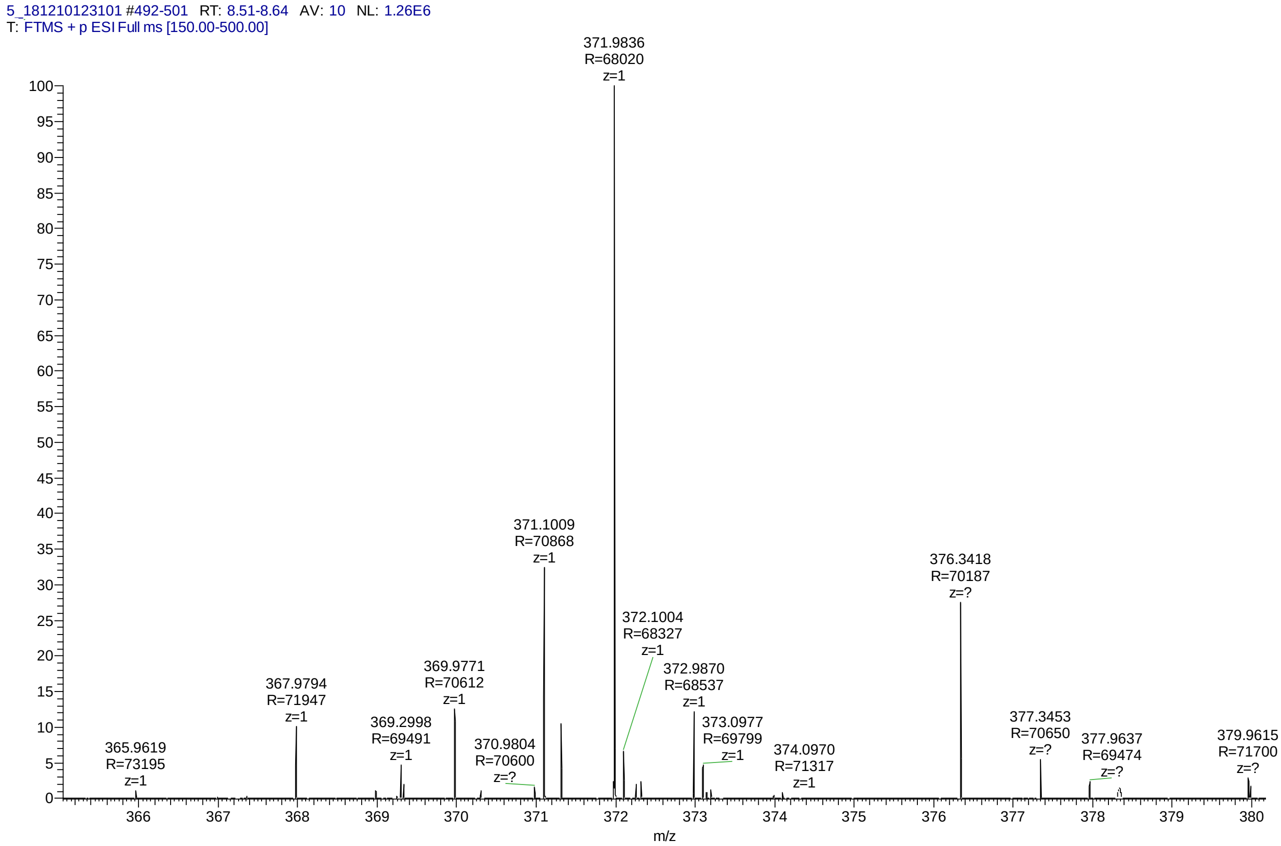

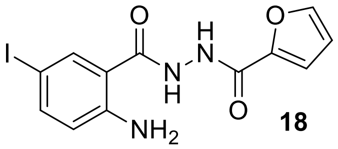

Chemical Formula: $C_{12}H_{11}IN_3O_3^+$
Exact Mass: 371,9840
Molecular Weight: 372,1380

2-amino-*N′*-benzoyl-4-chlorobenzohydrazide (**19**)

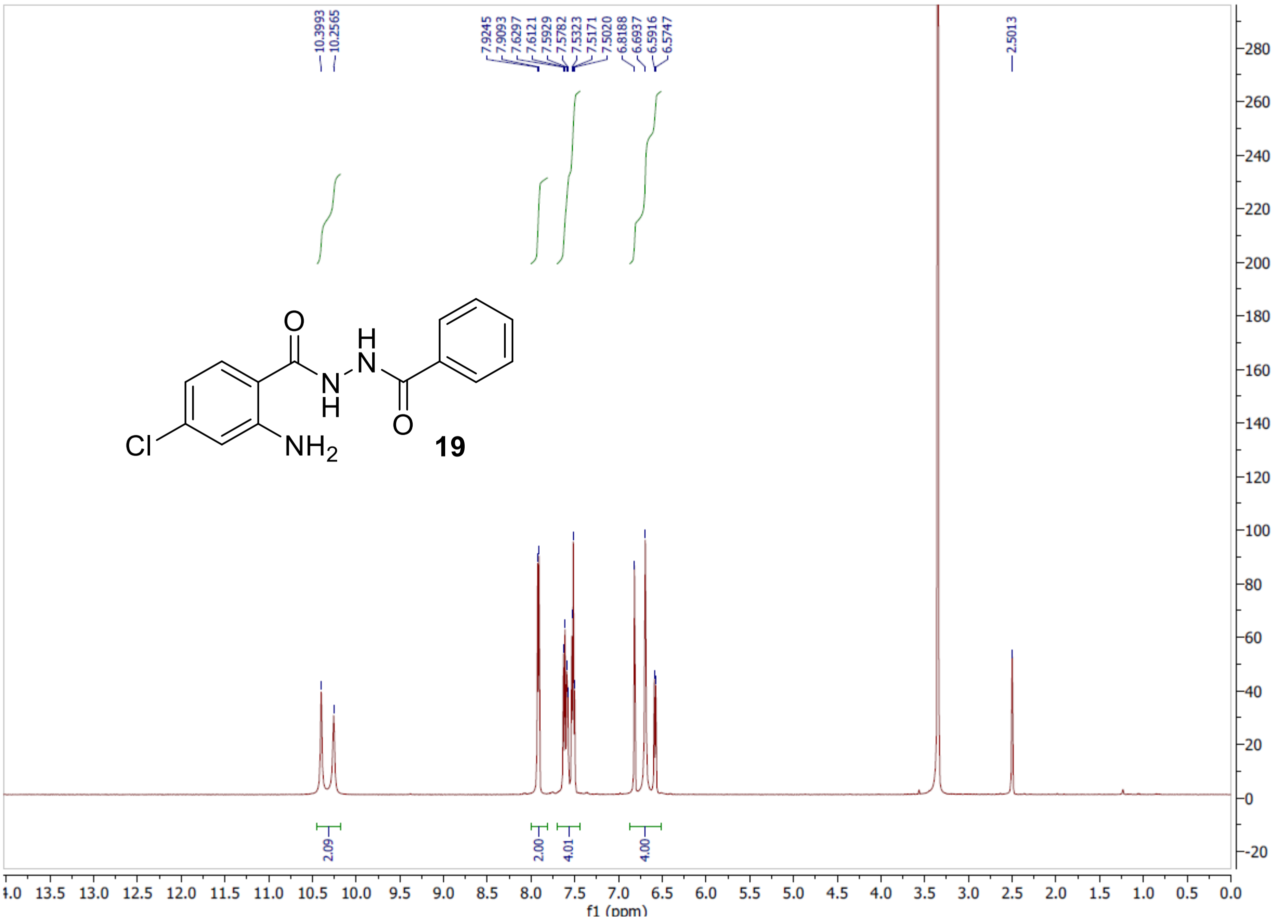

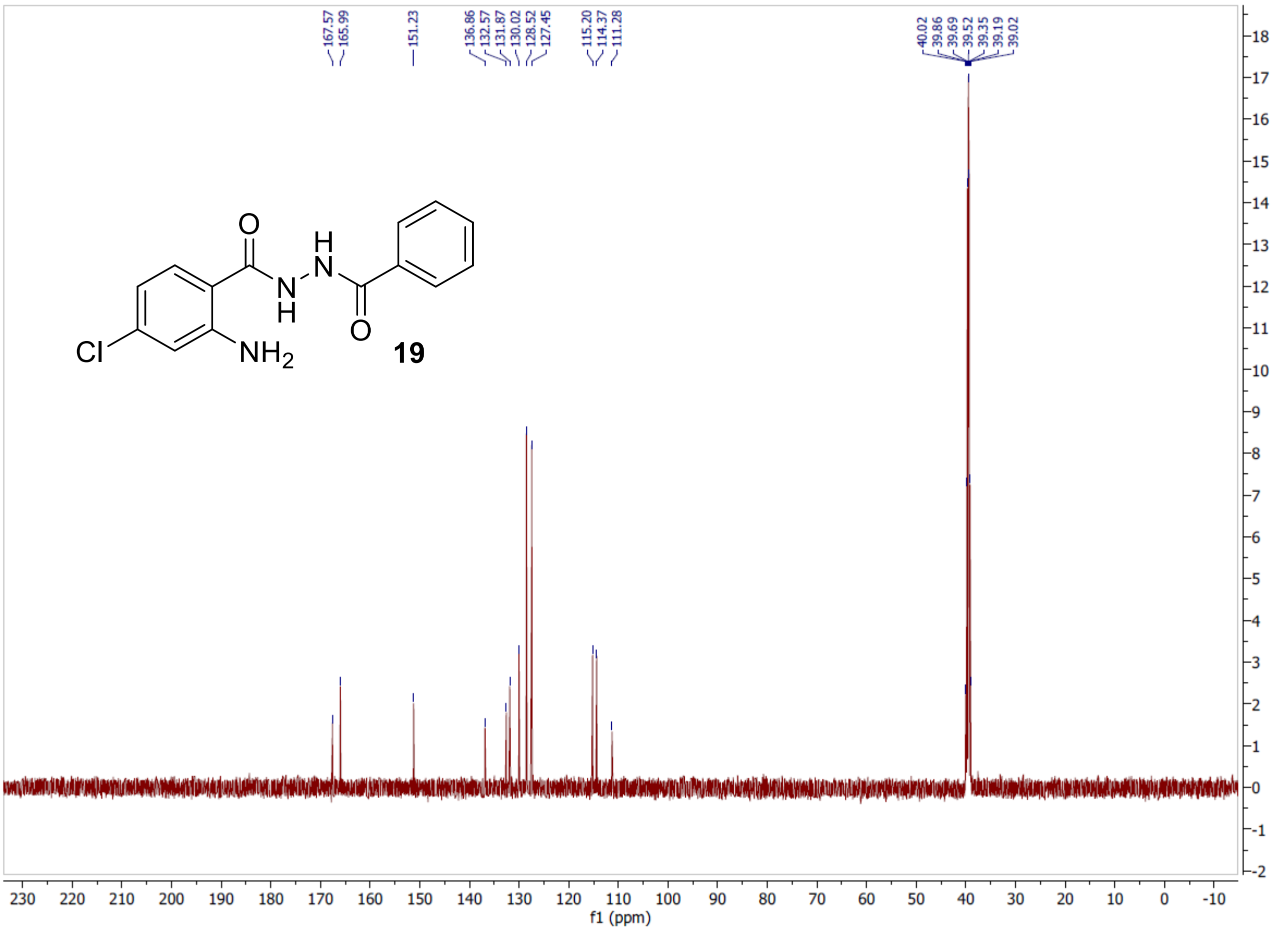

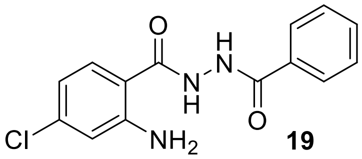

19

H+

Chemical Formula: $C_{14}H_{13}ClN_3O_2^+$
Exact Mass: 290,0691

5_181210123101 #419-428 RT: 7.47-7.60 AV: 10 NL: 2.05E6
T: FTMS + p ESI Full ms [150.00-500.00]

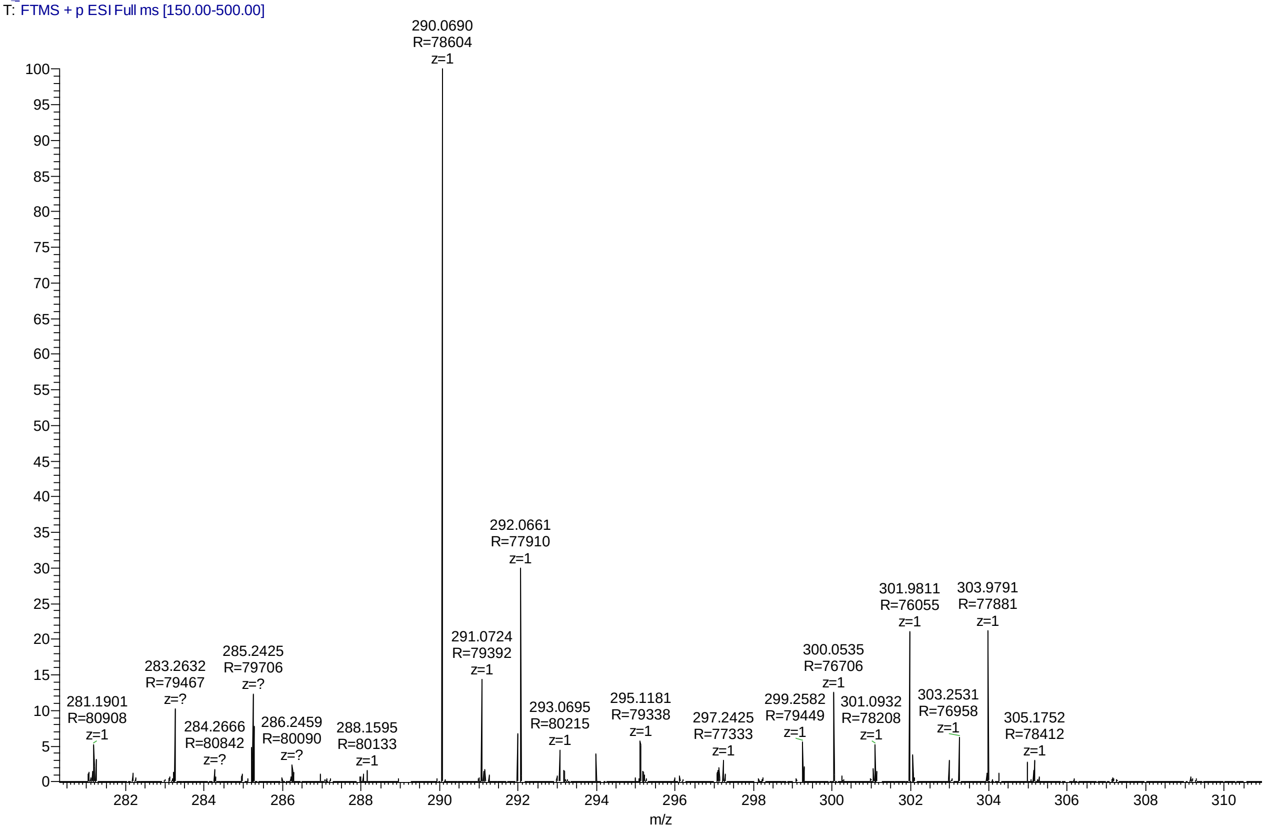

N′-(2-amino-4-chlorobenzoyl)furan-2-carbohydrazide (**20**)

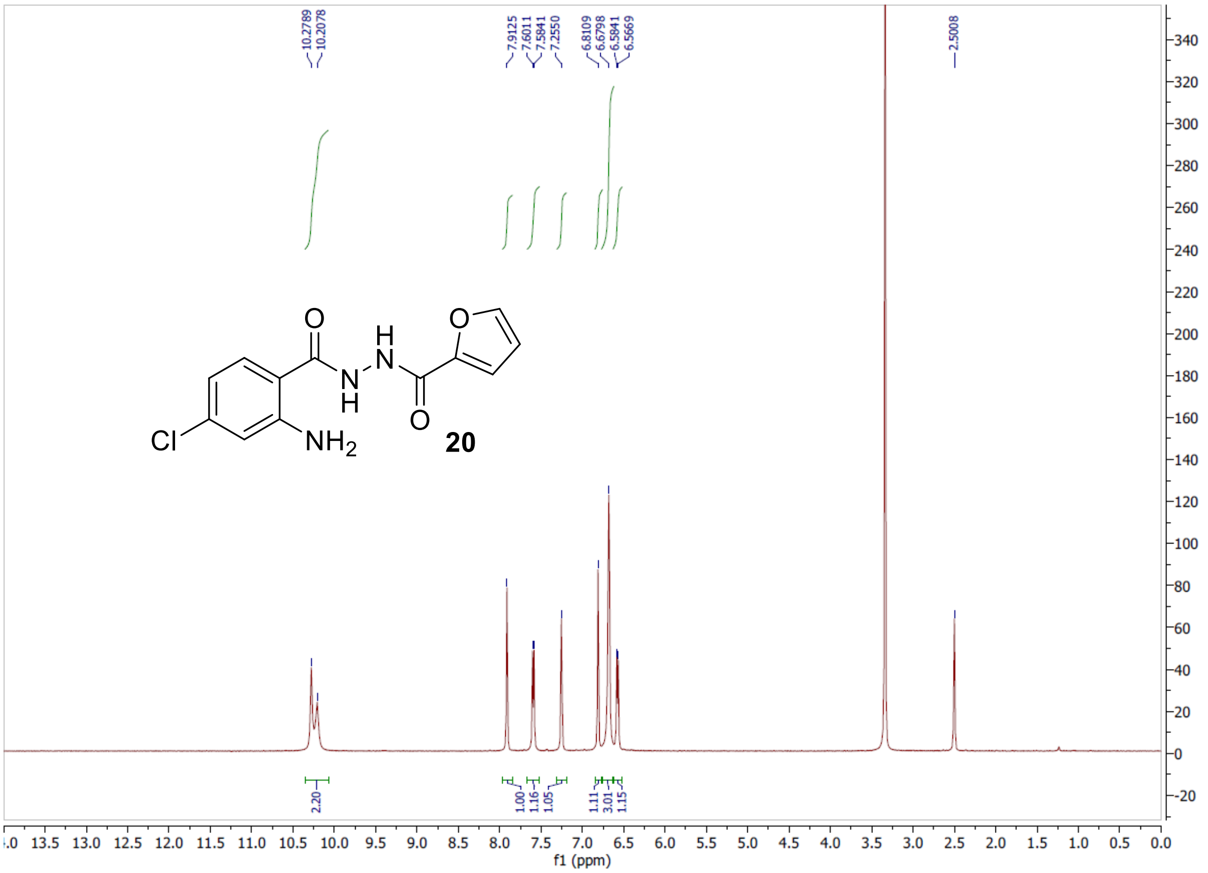

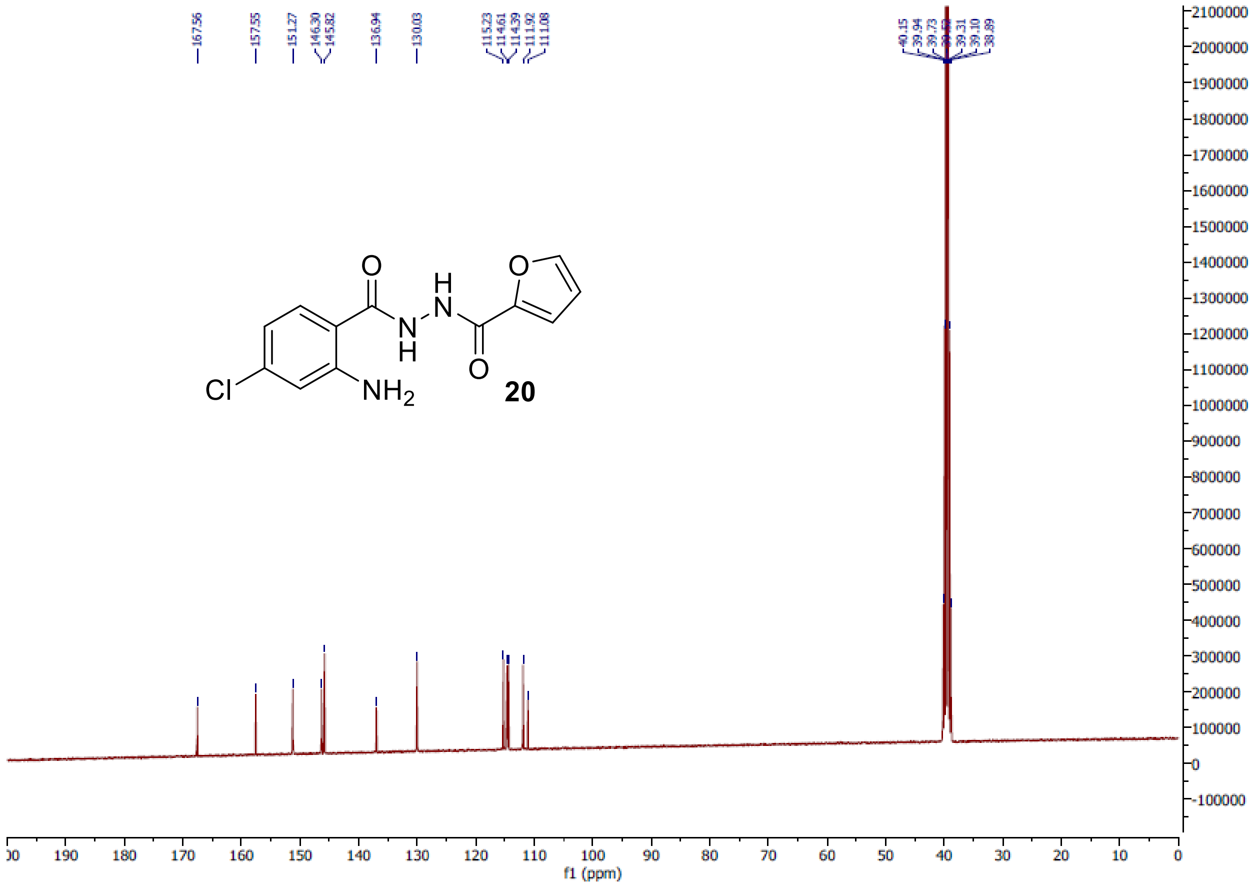

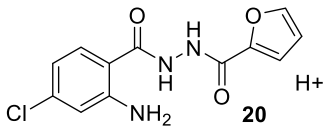

20

Chemical Formula: $C_{12}H_{11}ClN_3O_3^+$

Exact Mass: 280,0483

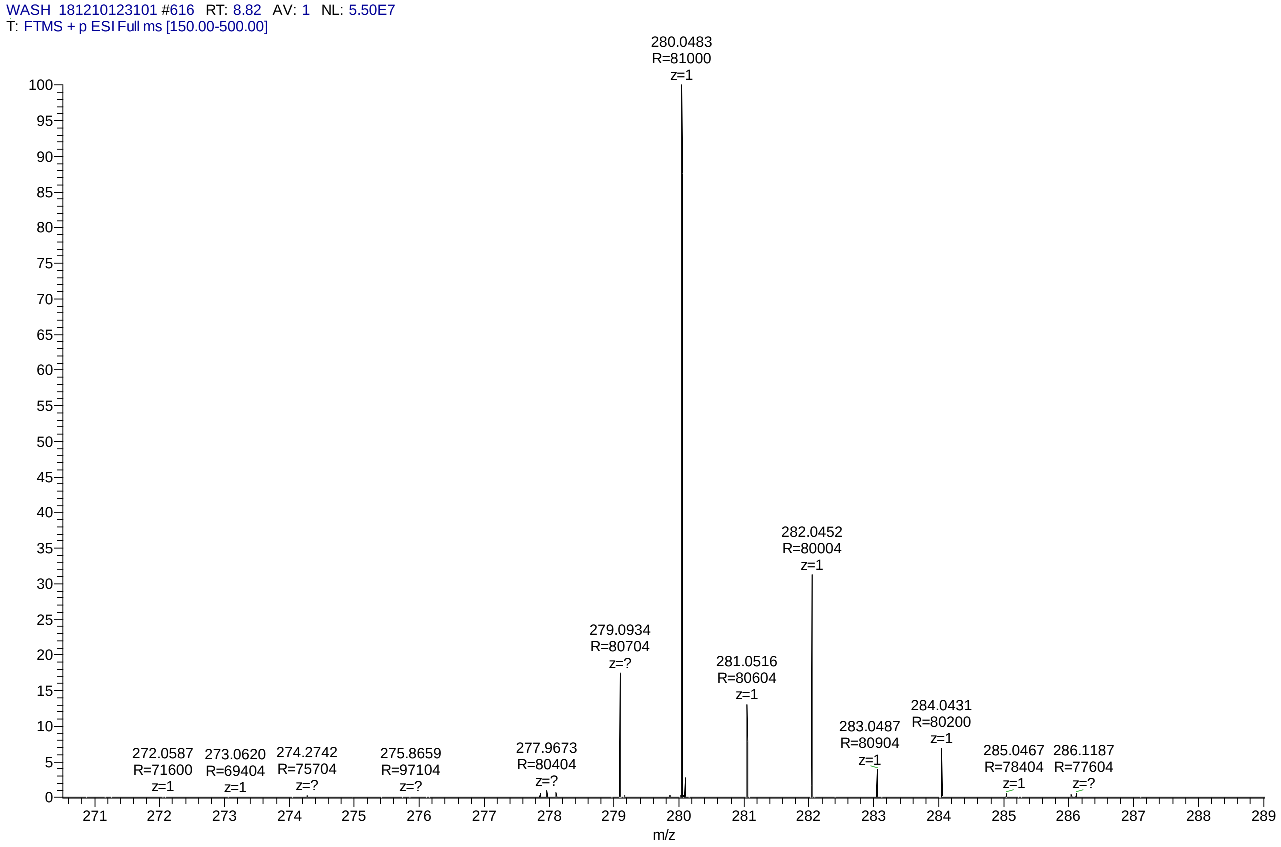

2-amino-*N′*-benzoyl-4-nitrobenzohydrazide (**21**)

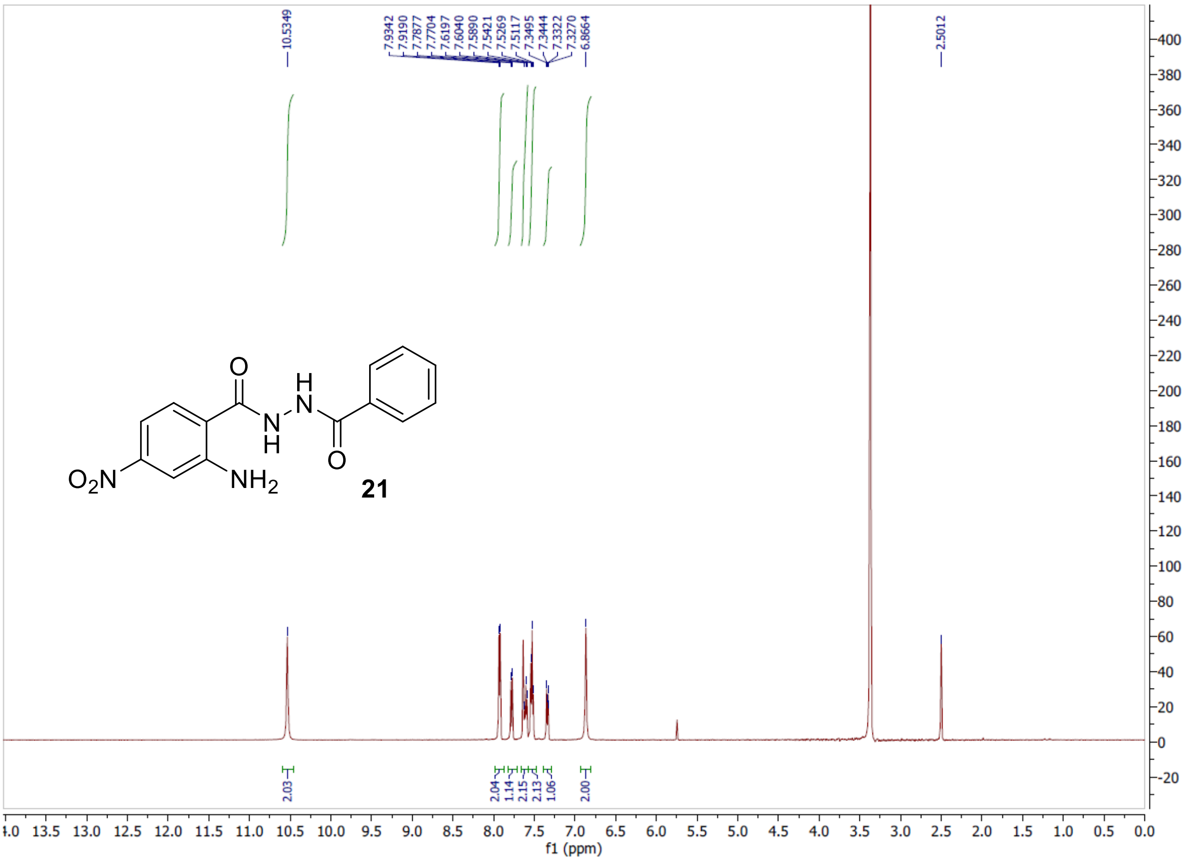

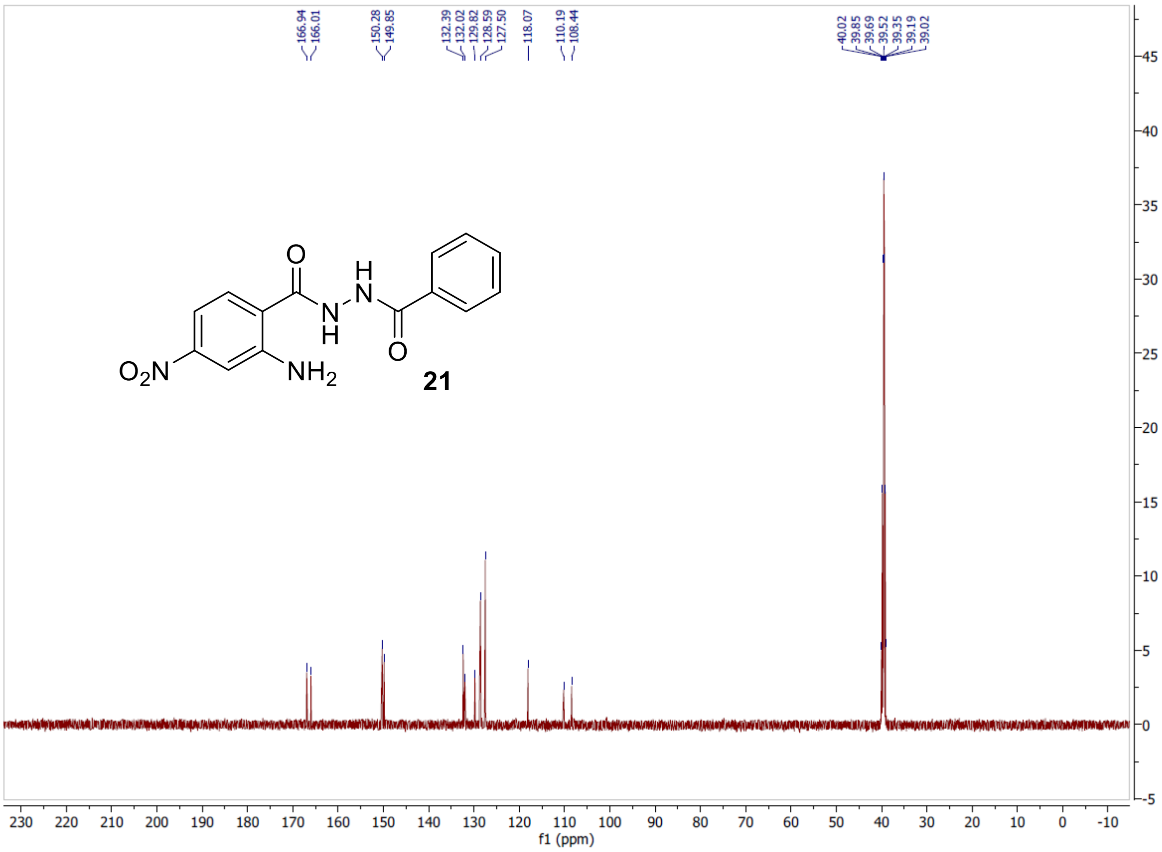

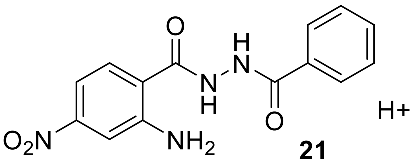

21

Chemical Formula: $C_{14}H_{13}N_4O_4^+$

Exact Mass: 301,0931

5_181210123101 #419-428 RT: 7.47-7.60 AV: 10 NL: 4.36E5
T: FTMS + p ESI Full ms [150.00-500.00]

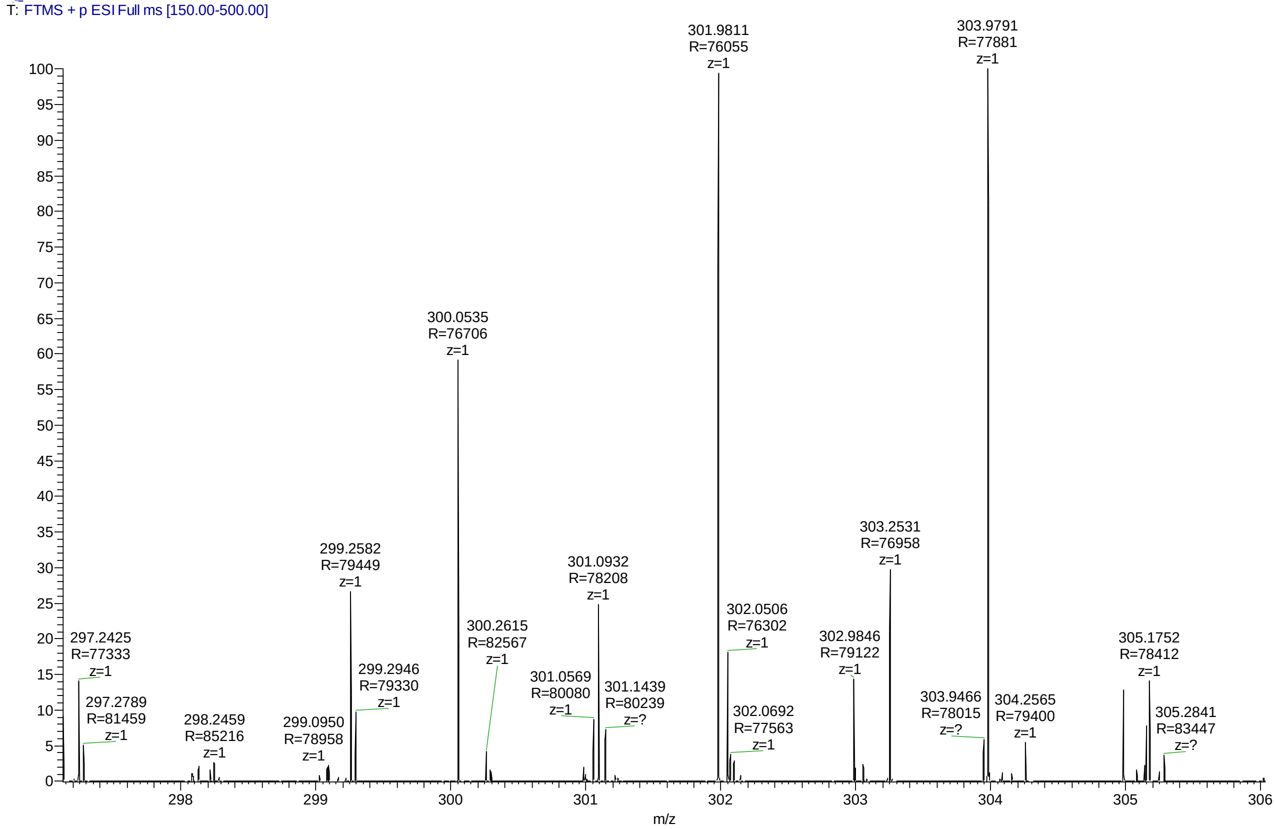

N′-(2-amino-4-nitrobenzoyl)furan-2-carbohydrazide (**22**)

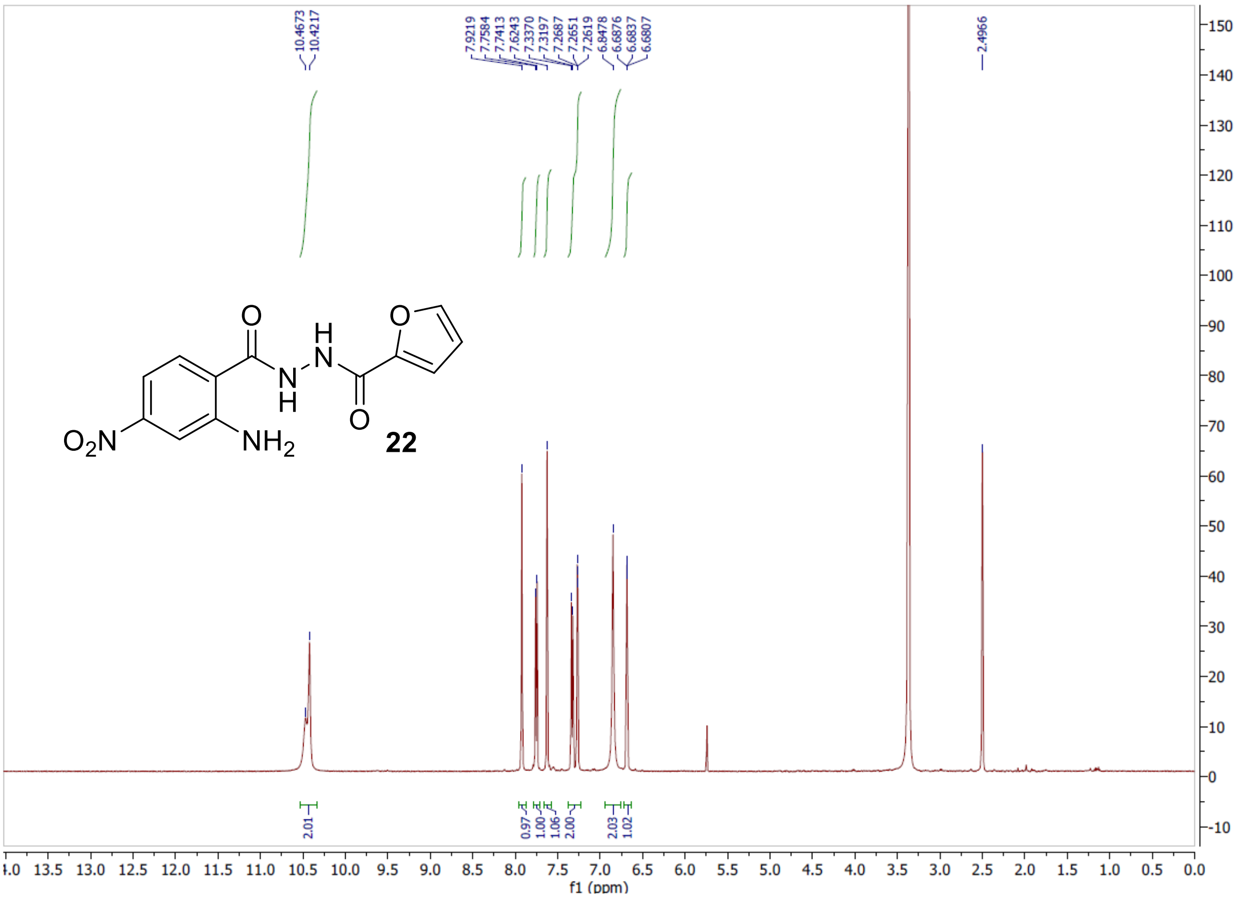

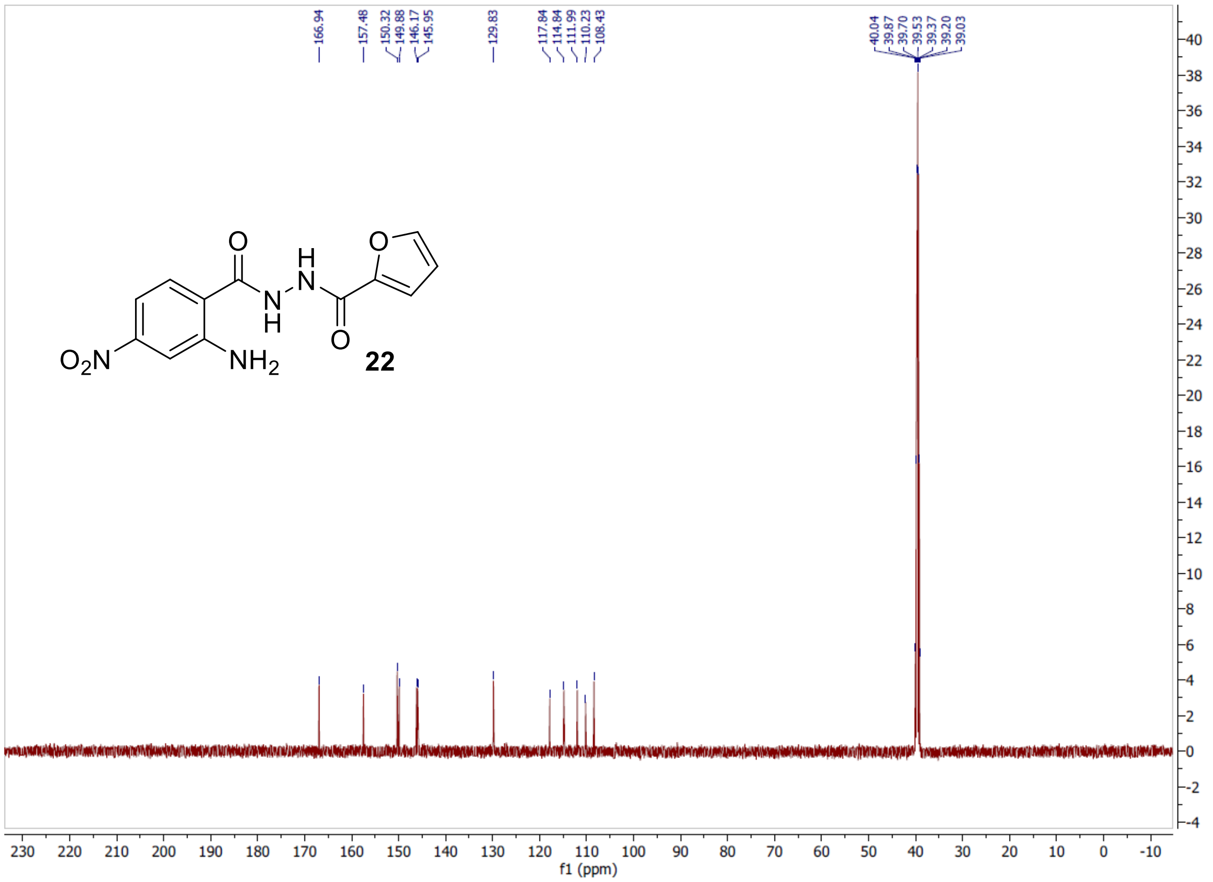

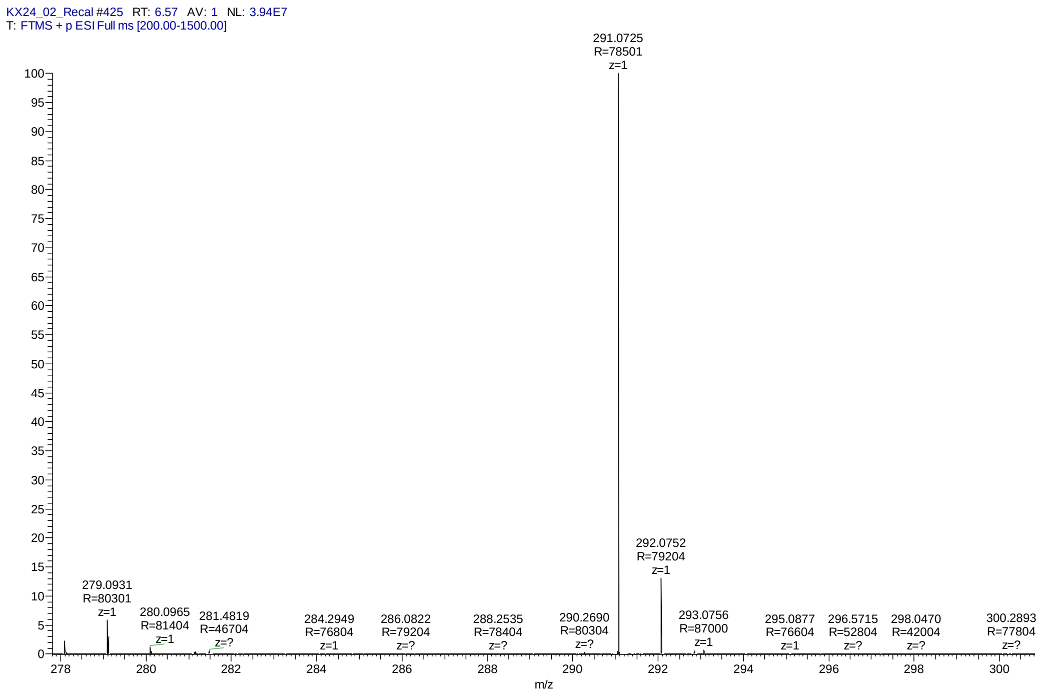

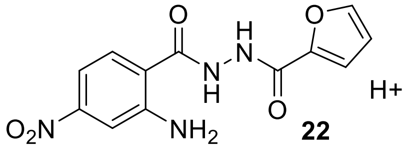

22

Chemical Formula: $C_{12}H_{11}N_4O_5^+$
Exact Mass: 291,0724

2-amino-*N′*-benzoyl-5-nitrobenzohydrazide (**23**):

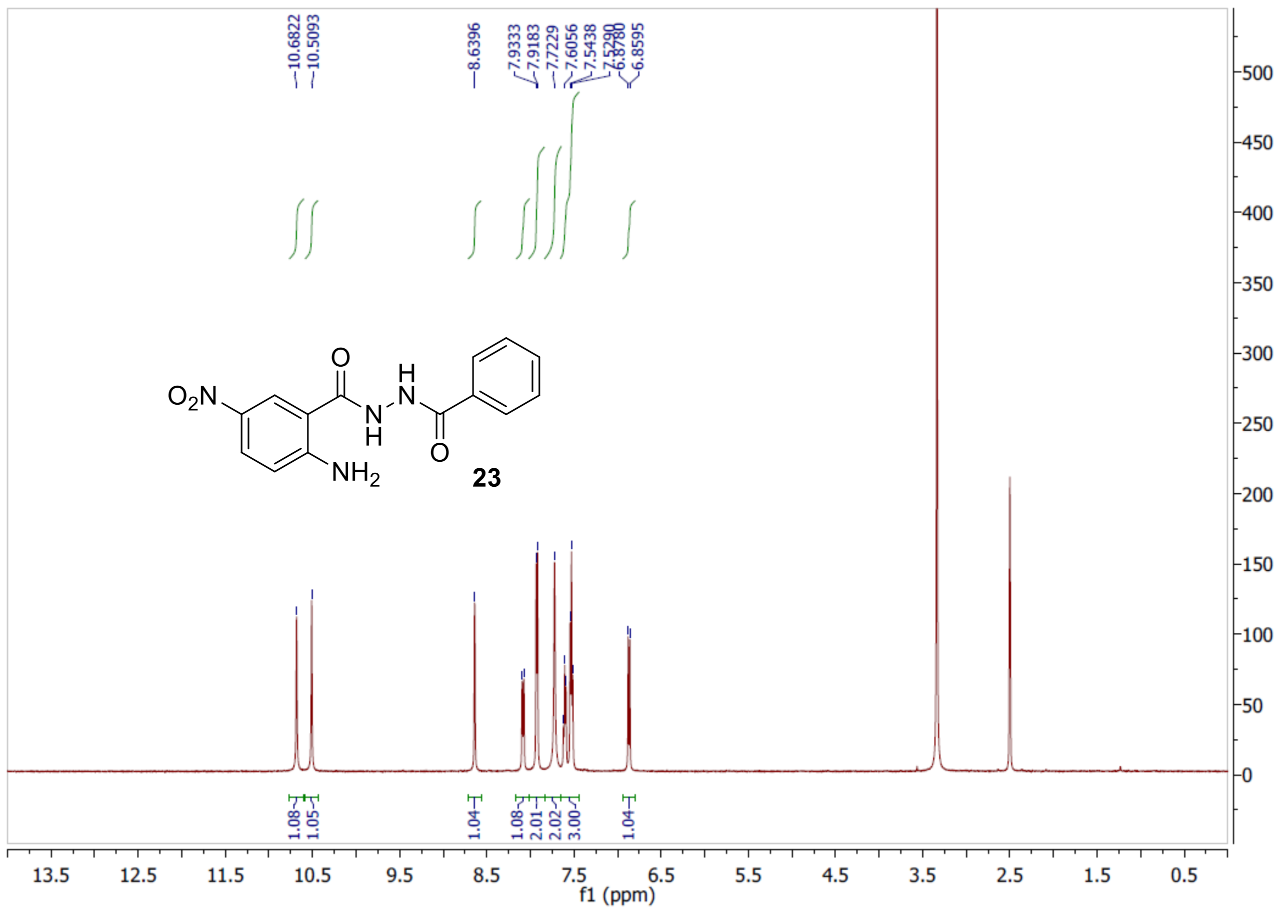

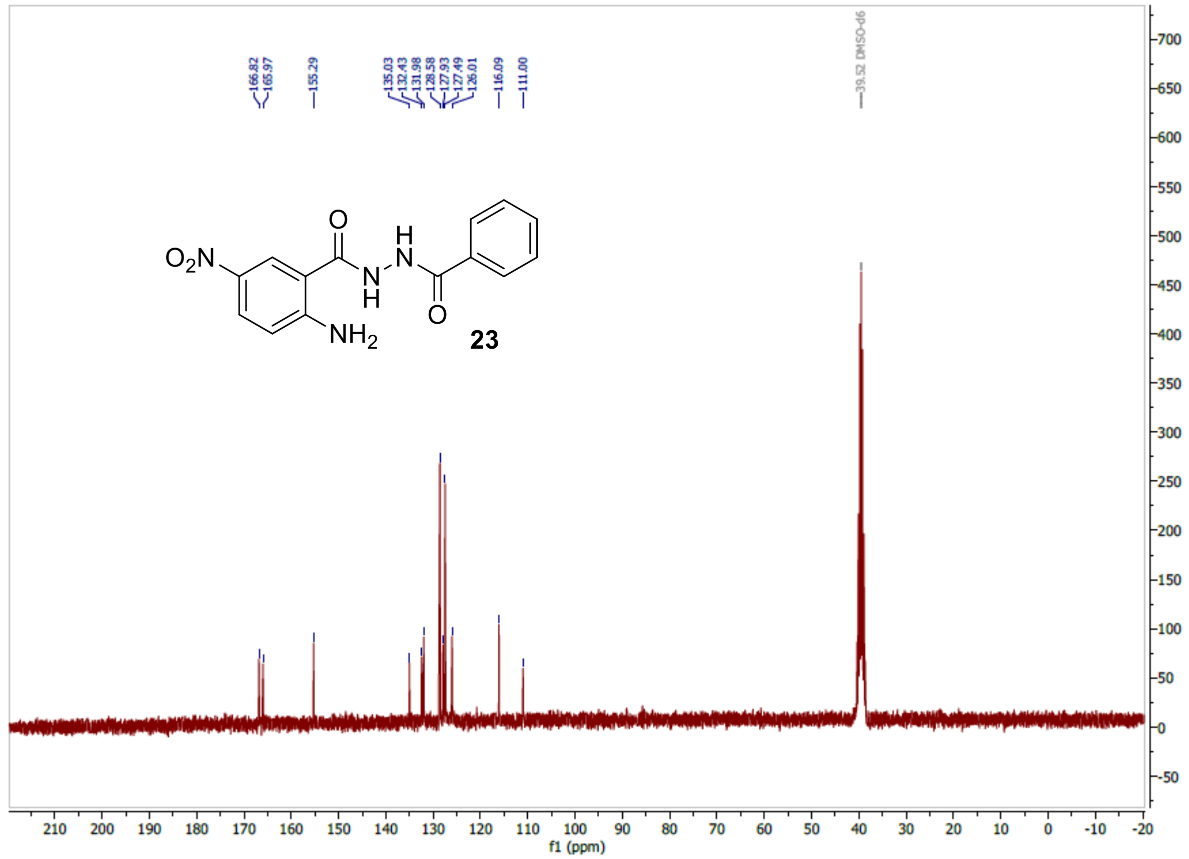

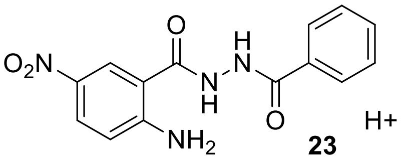

Chemical Formula: $C_{14}H_{13}N_4O_4^+$
Exact Mass: 301,0931
Molecular Weight: 301,2769

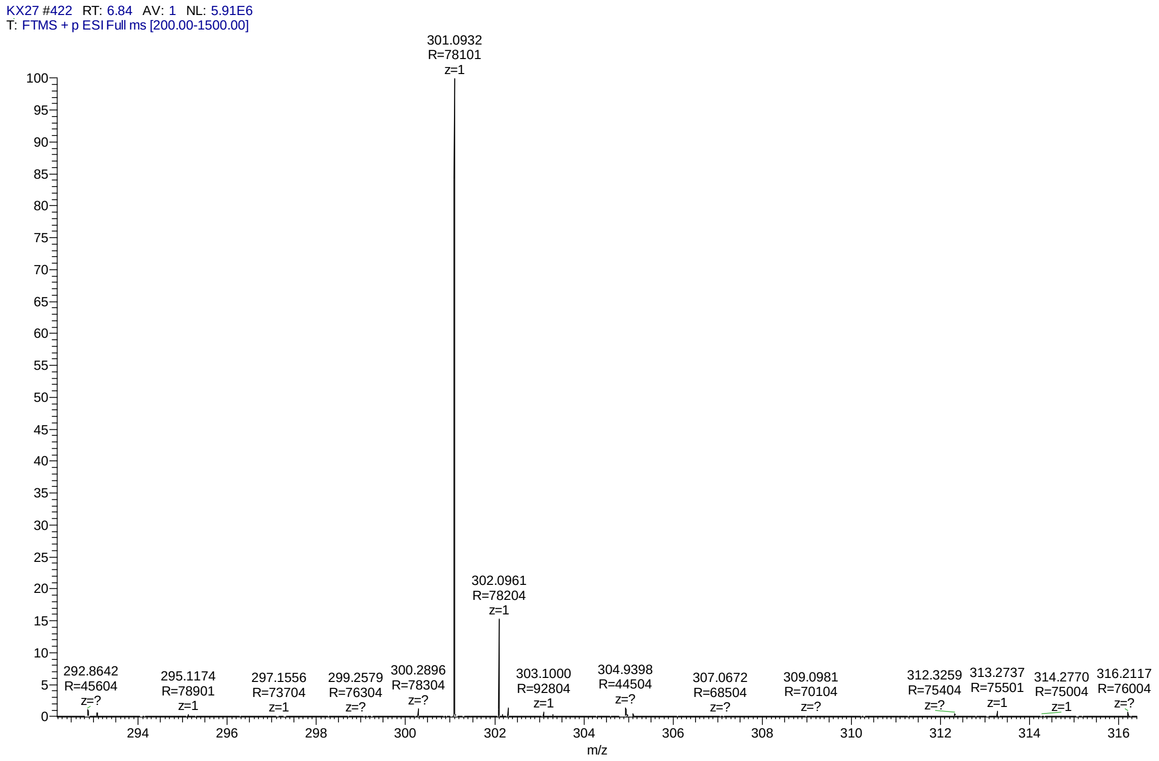

N'-(2-amino-5-nitrobenzoyl)furan-2-carbohydrazide (**24**)

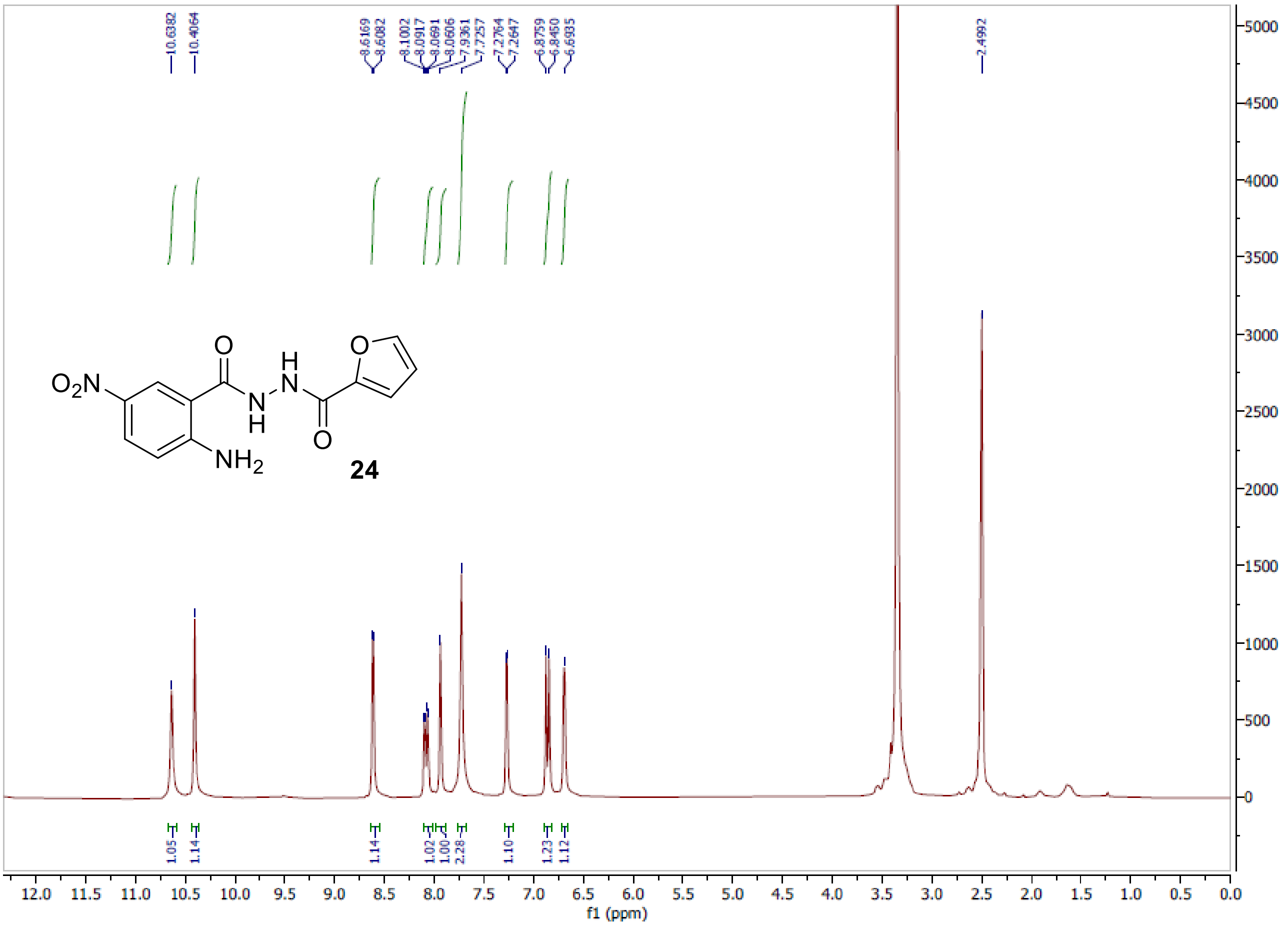

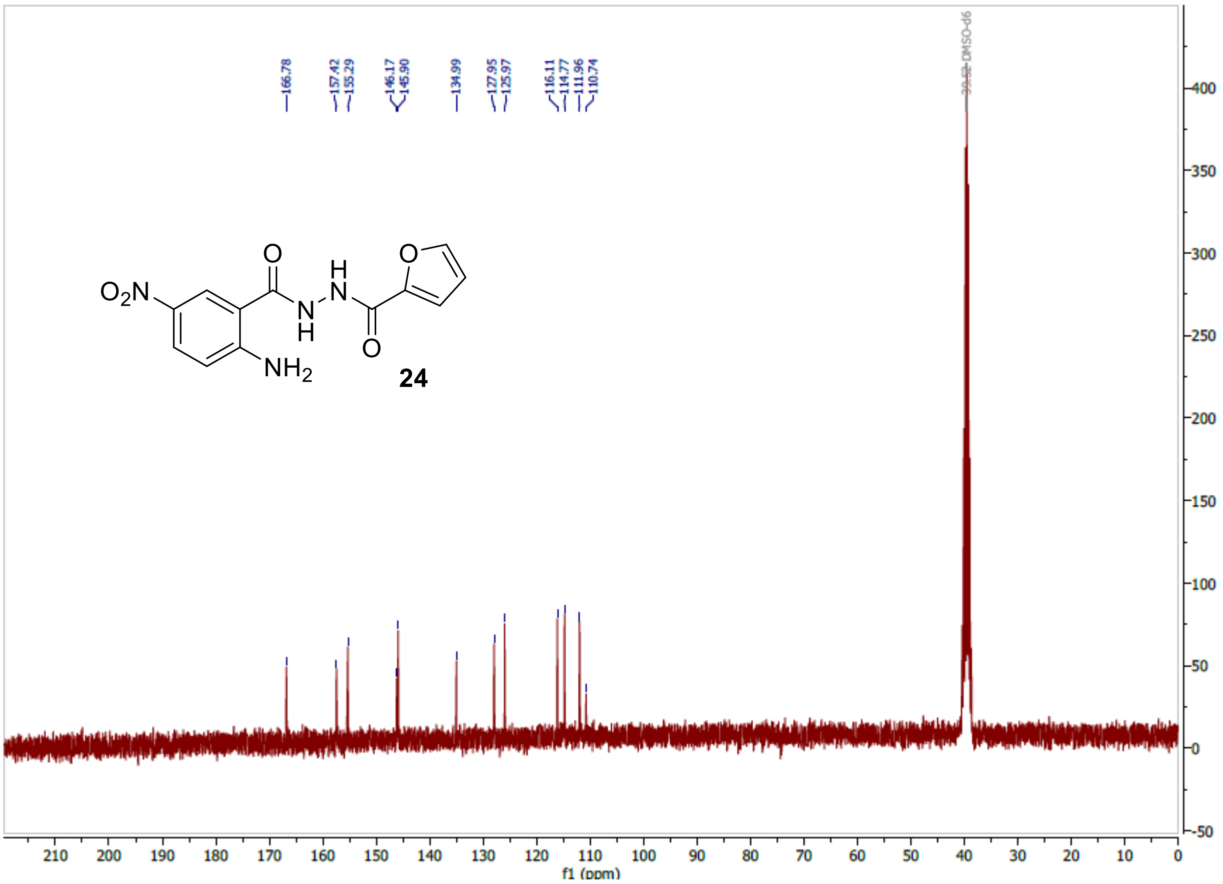

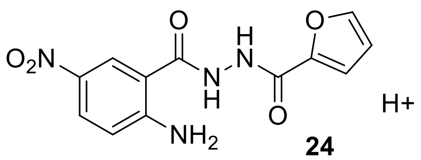

24

Chemical Formula: $C_{12}H_{11}N_4O_5^+$

Exact Mass: 291,0724

Molecular Weight: 291,2390

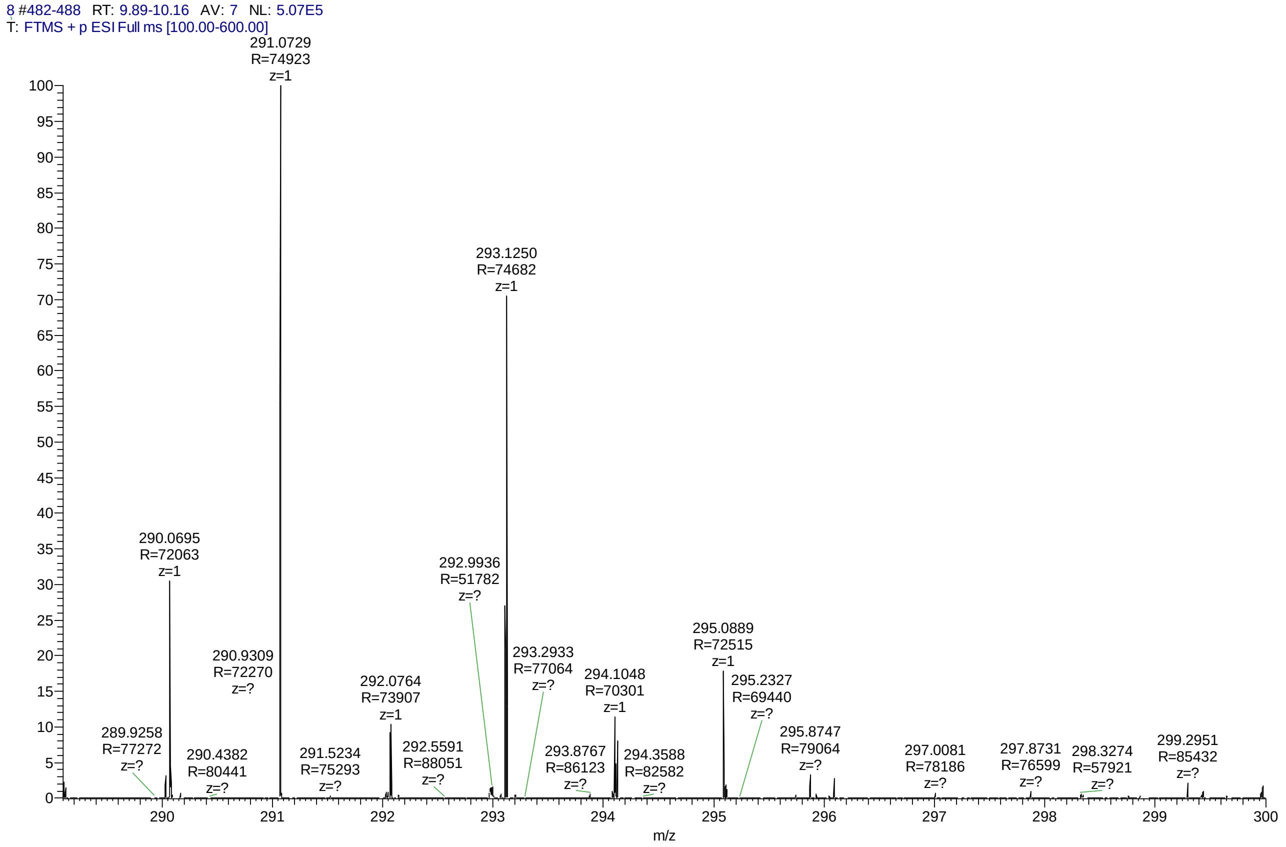

S.2. Gel electrophoresis pictures of AA DACHZs 1-24 and AA II a-h

S.2.1.

1 2 3 4 5 6 7 8 9 10 11

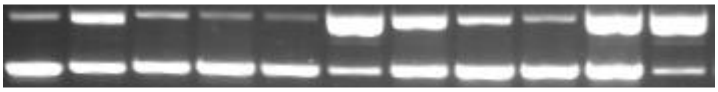

Form II% : 37 20 16 16 55 42 28 21 42 59

Form III% : 0 0 0 0 4 4 0 0 4 6

lane	312nm, 15cm, 30min, pH=6.8, DMSO, C = 500 μM				
1	DNA + UV +	Control	**lane**		
2	DNA + UV +	**II d**	7	DNA + UV +	**II d**
3	DNA + UV +	**13**	8	DNA + UV +	**13**
4	DNA + UV +	**14**	9	DNA + UV +	**14**
5	DNA + UV +	**15**	10	DNA + UV +	**15**
6	DNA + UV +	**16**	11	DNA + UV +	**16**

Gel S.2.1: Compounds 13-16 and II d

S.2.2.

1 2 3 4 5 6 7 8 9 10 11 12 13 14

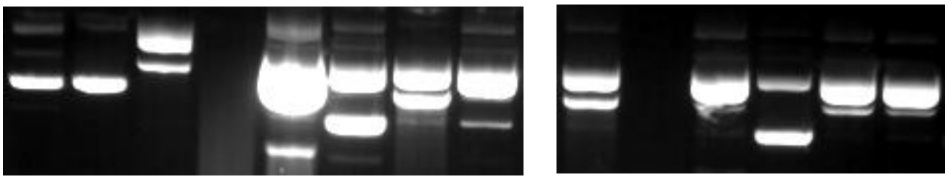

Form II% : 63 - 0 0 49 77 54 - 100 20 73 87

Form III% : 37 - 80 51 38 0 46 - 0 0 2 13

lane	312nm, 15cm, 30min, pH=6.8 DMSO, C = 500 μM				
1	DNA + UV	Control			
2	DNA	Control	**lane**		
3	DNA + UV +	**II c**	9	DNA + UV +	**II c**
4	DNA + UV +	**9**	10	DNA + UV +	**9**
5	DNA + UV +	**10**	11	DNA + UV +	**10**
6	DNA + UV +	**11**	12	DNA + UV +	**11**
7	DNA + UV +	**12**	13	DNA + UV +	**12**
8	DNA + UV +	**8**	14	DNA + UV +	**8**

Gel S.2.2: Compounds 8-12 and II c

S.2.3.

1 2 3 4 5 6 7 8 9 10 11 12 13 14 15 16

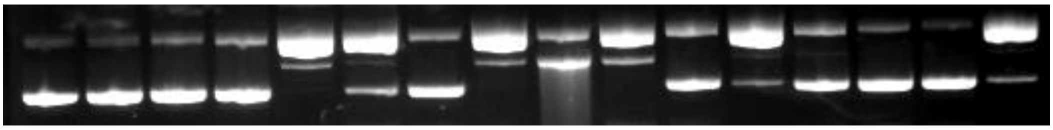

Form II% : 4 11 19 88 65 18 84 35 75 25 78 21 7 - 83

Form III% : 0 0 0 12 6 0 16 65 25 0 4 0 0 - 0

lane	312nm, 15cm, 30min, pH=6.8, DMSO, C= 500 μM		lane		
1	DNA + UV +	Control	9	DNA + UV +	**9**
2	DNA + UV +	**2**	10	DNA + UV +	**10**
3	DNA + UV +	**3**	11	DNA + UV +	**11**
4	DNA + UV +	**4**	12	DNA + UV +	**12**
5	DNA + UV +	**5**	13	DNA + UV +	**13**
6	DNA + UV +	**6**	14	DNA + UV +	**14**
7	DNA + UV +	**7**	15	DNA + UV +	**15**
8	DNA + UV +	**8**	16	DNA + UV +	**16**

Gel S.2.3: Compounds 1-16

S.2.4.

1 2 3 4 5 6 7 8 9 10 11 12 13 14 15 16

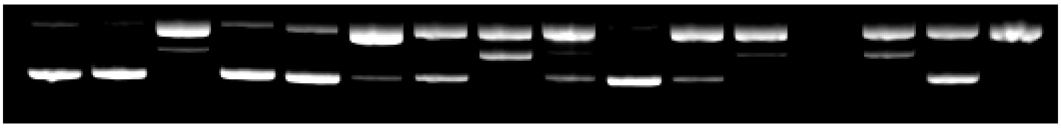

Form II% : 0 92 5 13 90 56 64 77 0 80 95 - 80 42 100

Form III% : 0 8 0 0 0 0 36 2 0 0 5 - 20 0 0

Σειρά	312nm, 15cm, 30min, pH=6.8, DMSO, C= 500 μM				
1	DNA + UV +	Control	9	DNA + UV +	**II d**
2	DNA + UV +	**II a**	10	DNA + UV +	**II g**
3	DNA + UV +	**irrelevant**	11	DNA + UV +	**II f**
4	DNA + UV +	**II h**	12	DNA + UV +	**II c**
5	DNA + UV +	**irrelevant**	13	DNA + UV +	**9**
6	DNA + UV +	**II e**	14	DNA + UV +	**10**
7	DNA + UV +	**irrelevant**	15	DNA + UV +	**11**
8	DNA + UV +	**II b**	16	DNA + UV +	**12**

Gel S.2.4: Compounds II a-g, 9-12

S.2.5.

1 2 3 4 5 6 7 8 9 10 11 12 13 14 15 16

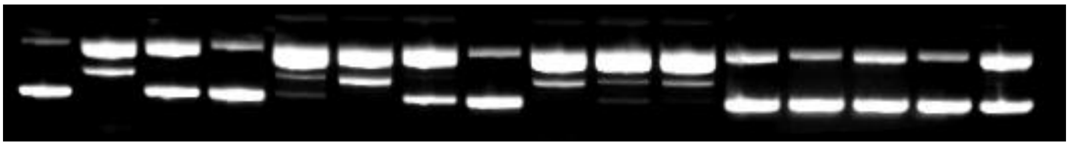

Form II% : 69 42 13 85 68 59 3 80 89 87 24 13 25 11 41

Form III% : 31 0 0 9 32 0 0 20 7 13 0 0 0 0 0

**lane**	**312nm, 15cm, 30min, pH=6.8, DMSO, C= 500 μM**				
1	DNA + UV +	Control	**lane**		
2	DNA + UV +	**5**	10	DNA + UV +	**17**
3	DNA + UV +	**6**	11	DNA + UV +	**18**
4	DNA + UV +	**7**	12	DNA + UV +	**19**
5	DNA + UV +	**8**	13	DNA + UV +	**20**
6	DNA + UV +	**5**	14	DNA + UV +	**19**
7	DNA + UV +	**6**	15	DNA + UV +	**20**
8	DNA + UV +	**7**	16	DNA + UV +	**II f**
9	DNA + UV +	**8**			

Gel S.2.5: Compounds II f, 5-8, 17-20

S.2.6.

1 2 3 4 5 6 7 8 9 10 11 12 13 14 15 16

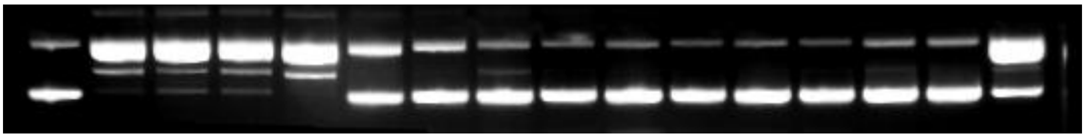

Form II% : 80 80 74 73 25 11 0 0 0 0 0 0 0 0 55

Form III% : 17 11 15 27 0 0 7 0 0 0 0 0 3 0 4

**lane**	**312nm, 15cm, 30min, pH=6.8, DMSO, Cτελ= 500 μM**		**lane**		
1	DNA + UV +	Control	8	DNA + UV +	**21**
2	DNA + UV +	**17**	9	DNA + UV +	**22**
3	DNA + UV +	**18**	10	DNA + UV +	**21**
4	DNA + UV +	**17**	11	DNA + UV +	**22**
5	DNA + UV +	**18**	12	DNA + UV +	**21**
6	DNA + UV +	**19**	13	DNA + UV +	**22**
7	DNA + UV +	**20**	14	DNA + UV +	**II g**
			15	DNA + UV +	**II h**
			16	DNA + UV +	**II e**

Gel S.2.6: Compounds II e,g,h, 17-22

S.2.7.

1 2 3 4 5 6 7 8 9 10 11 12 13 14

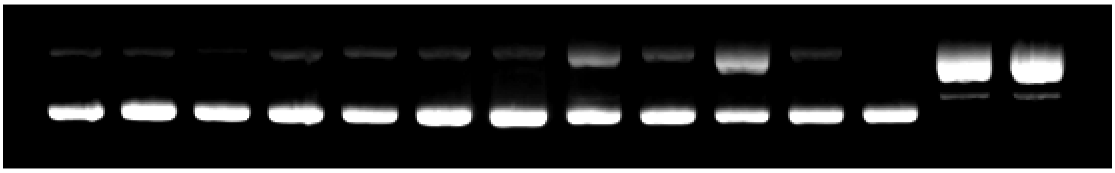

Form II% :	0	0	2	3	1	1	20	6	38	2	0	96	96	
Form III% :	0	0	0	0	0	0	0	0	0	0	0	4	4	

**lane**	**312nm, 15cm, 30min, pH=6.8, DMSO, C=500 μM**				
1	DNA + UV +	Control	**lane**		
2	DNA + UV +	**1**	10	DNA + UV +	**13**
3	DNA + UV +	**2**	11	DNA + UV +	**14**
4	DNA + UV +	**3**	12	DNA + UV +	**15**
5	DNA + UV +	**4**	13	DNA + UV +	**16**
6	DNA + UV +	**1**	14	DNA + UV +	**8**
7	DNA + UV +	**2**			
8	DNA + UV +	**3**			
9	DNA + UV +	**4**			

Gel S.2.7: Compounds 1-4, 8, 13-16

S.2.8.

1 2 3 4 5 6

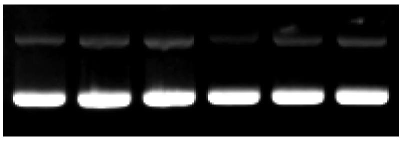

Form II% :	0	0	0	0	0	0
Form III% :	0	0	0	0	0	0

**lane**	**312nm, 15cm, 30min, pH=6.8, DMSO, C= 500 μM**	
1	DNA + UV +	**23**
2	DNA + UV +	**24**
3	DNA + UV +	**23**
4	DNA + UV +	**24**
5	DNA + UV +	**23**
6	DNA + UV +	**24**

Gel S.2.8: Compounds 23-24

S.3. Gel electrophoresis pictures of 9 at various concentrations, pH studies and mechanistic studies

S.3.1

1 2 3 4 5 6 7 8 9 10 11 12 13 14

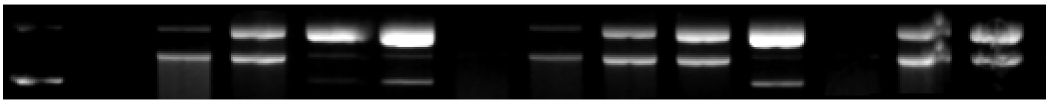

Form II% :	-	7	50	92	84	-	14	46	60	85	-	41	62	
Form III% :	-	93	50	4	3	-	86	54	40	2	-	59	38	

lane	312nm, 15cm, 30min, pH=6.8, DMSO, compound 9		lane		
1	DNA + UV +	Control	8	DNA + UV +	200 µM
2	DNA + UV +	400 µM	9	DNA + UV +	100 µM
3	DNA + UV +	200 µM	10	DNA + UV +	50 µM
4	DNA + UV +	100 µM	11	DNA + UV +	10 µM
5	DNA + UV +	50 µM	12	DNA + UV +	400 µM
6	DNA + UV +	10 µM	13	DNA + UV +	200 µM
7	DNA + UV +	400 µM	14	DNA + UV +	200 µM

Gel S.3.1: Compound 9 from 400 to 10 µM

S.3.2

1 2 3 4 5 6 7 8 9 10 11 12 13 14 15 16

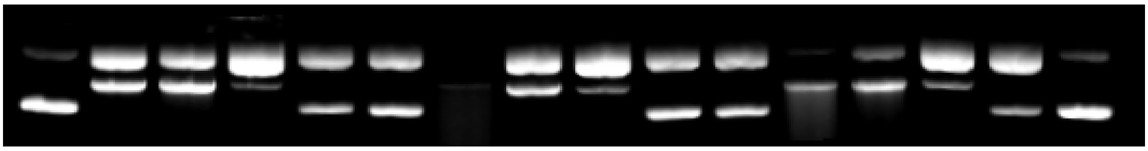

Form II% :	59	49	93	53	44	-	66	88	43	47	-	24	86	70	2
Form III% :	41	51	7	0	0	-	34	12	0	0	-	76	14	0	0

lane	312nm, 15cm, 30min, pH=6.8, DMSO, Compound 9		lane		
1	DNA + UV +	Control	9	DNA + UV +	10µM
2	DNA + UV +	100µM	10	DNA + UV +	1µM
3	DNA + UV +	50µM	11	DNA + UV +	500nM
4	DNA + UV +	10µM	12	DNA + UV +	100µM
5	DNA + UV +	1µM	13	DNA + UV +	50µM
6	DNA + UV +	500nM	14	DNA + UV +	10µM
7	DNA + UV +	100µM	15	DNA + UV +	1µM
8	DNA + UV +	50µM	16	DNA + UV +	500nM

Gel S.3.2: Compound 9 from 100 to 0.5 µM

S.3.3

1 2 3 4 5 6 7 8 9 10 11 12 13 14 15 16

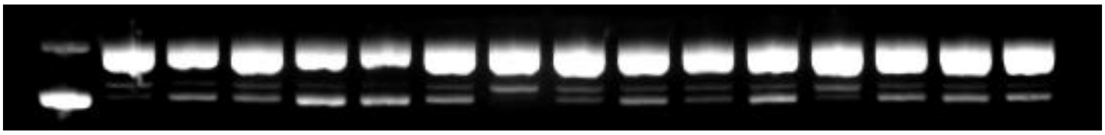

Form II% : 94 83 85 65 68 81 88 88 82 90 77 90 84 84 86

Form III% : 4 1 4 2 1 4 12 6 4 4 3 7 3 2 1

**lane**	**312nm, 15cm, 30min, DMSO, C= 5μM**		**lane**		
1	DNA + UV Control	**pH** 6.8	8	DNA + UV + **9**	pH 5
2	DNA + UV + **9**	pH 5	9	DNA + UV + **9**	pH 6.0
3	DNA + UV + **9**	pH 6.0	10	DNA + UV + **9**	pH 8.1
4	DNA + UV + **9**	pH 6.8	11	DNA + UV + **9**	pH 8.9
5	DNA + UV + **9**	pH 8.1	12	DNA + UV + **9**	pH 10
6	DNA + UV + **9**	pH 8.9	13	DNA + UV + **9**	pH 5
7	DNA + UV + **9**	pH 10	14	DNA + UV + **9**	pH 6.0
			15	DNA + UV + **9**	pH 8.1
			16	DNA + UV + **9**	pH 8.9

Gel S.3.3: Compound 9 from pH 5-10

S.3.4

1 2 3 4 5 6 7 8 9 10 11 12 13 14 15 16

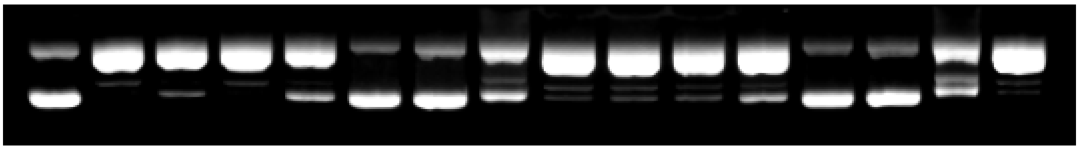

FII%: 97 89 97 65 0 0 41 92 90 90 72 0 0 42 96

FIII%: 3 0 3 1 0 0 6 4 3 2 2 0 0 16 2

**lane**	**312nm, 15cm, 30min, pH=6.8, DMSO, C=5μM**		**lane**		
1	DNA + UV +	Control	9	DNA + UV + **9**	air
2	DNA + UV + **9**	air	10	DNA + UV + **9**	argon
3	DNA + UV + **9**	argon	11	DNA + UV + **9**	L-cyst (1500 μM)
4	DNA + UV + **9**	L-cyst (1500 μM)	12	DNA + UV + **9**	KI (250 μM)
5	DNA + UV + **9**	KI (250 μM)	13	DNA + UV + **9**	NaN_3 (20 mM)
6	DNA + UV + **9**	NaN_3 (20 mM)	14	DNA + UV + **9**	D_2O
7	DNA + UV + **9**	D_2O	15	DNA + UV + **9**	DMSO 20%
8	DNA + UV + **9**	DMSO 20%	16	DNA + UV + **9**	air

Gel S.3.4: Compound 9, mechanistic studies

S.4. UV-vis spectra of AA DACHZs 1-24, in DMSO

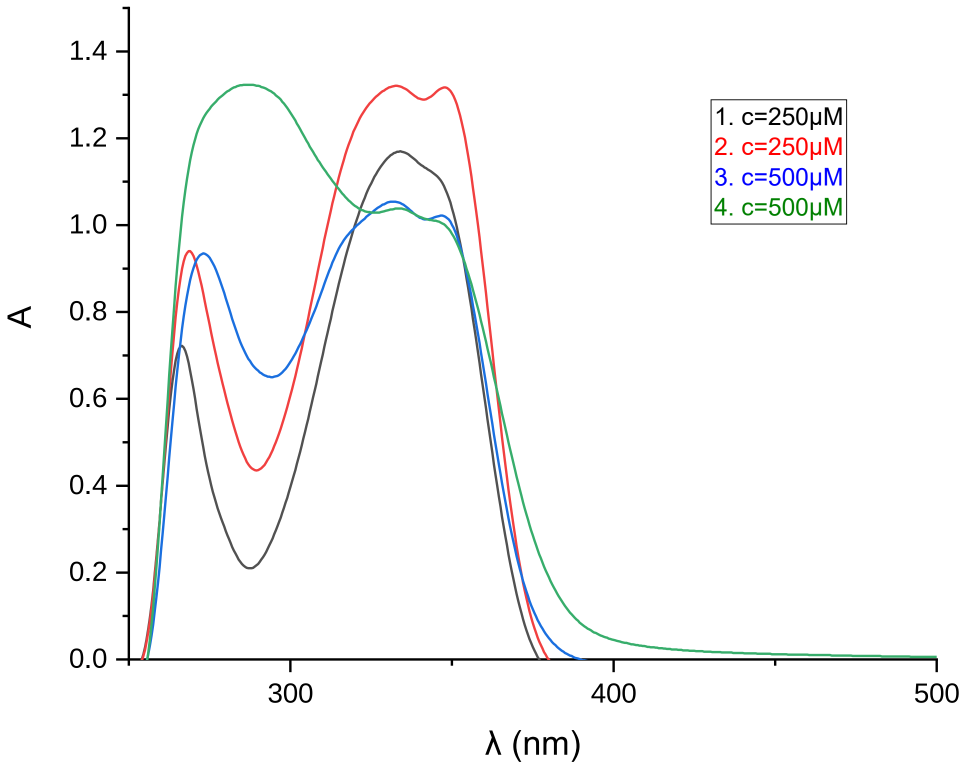

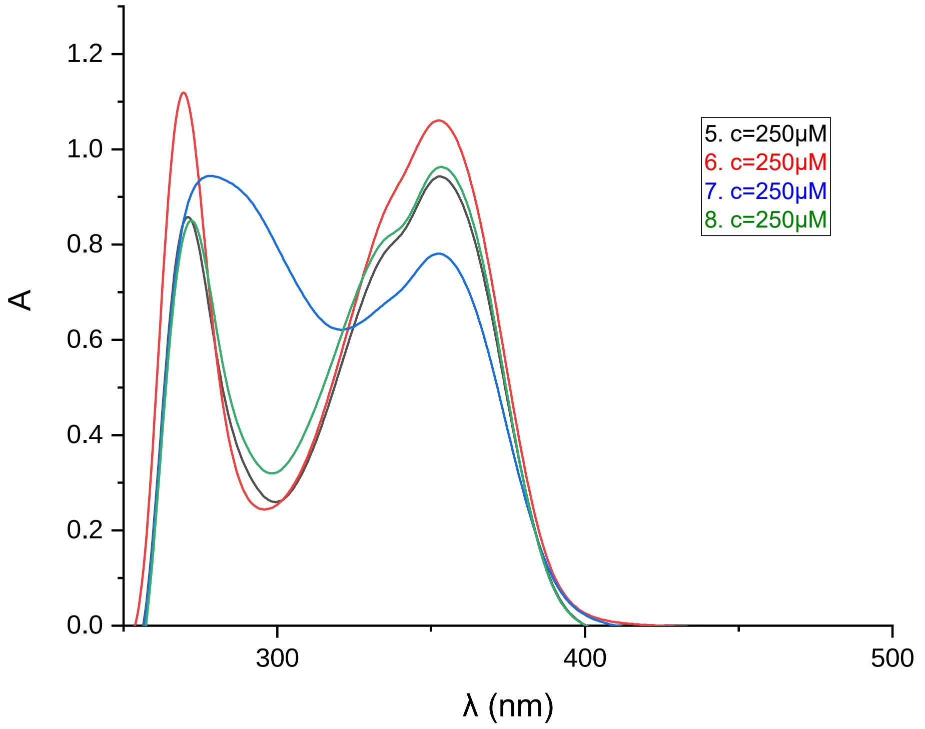

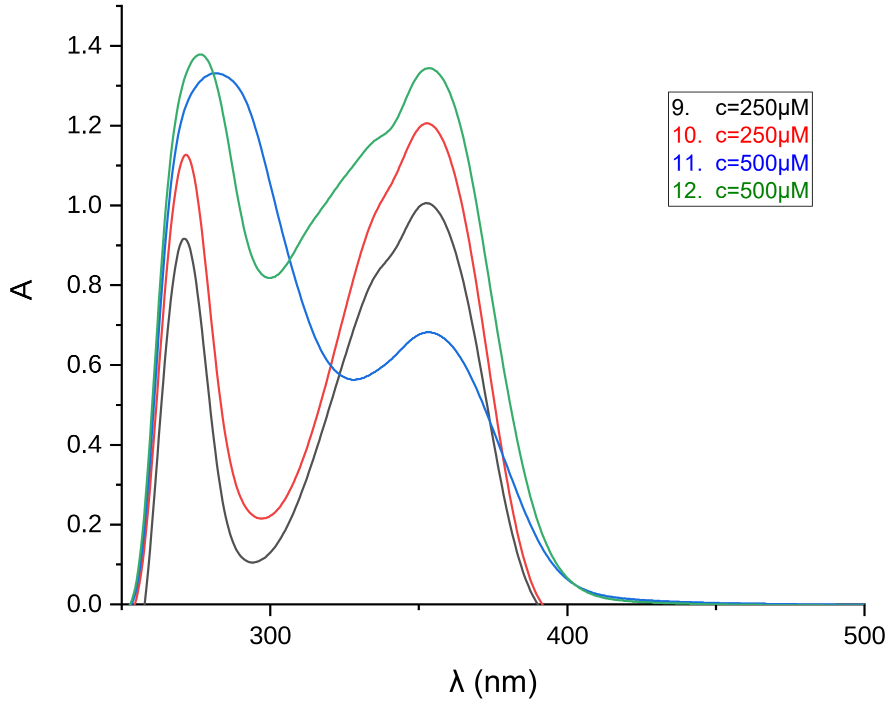
9. c=250µM
10. c=250µM
11. c=500µM
12. c=500µM
A
λ (nm)
0.0
0.2
0.4
0.6
0.8
1.0
1.2
1.4
300
400
500

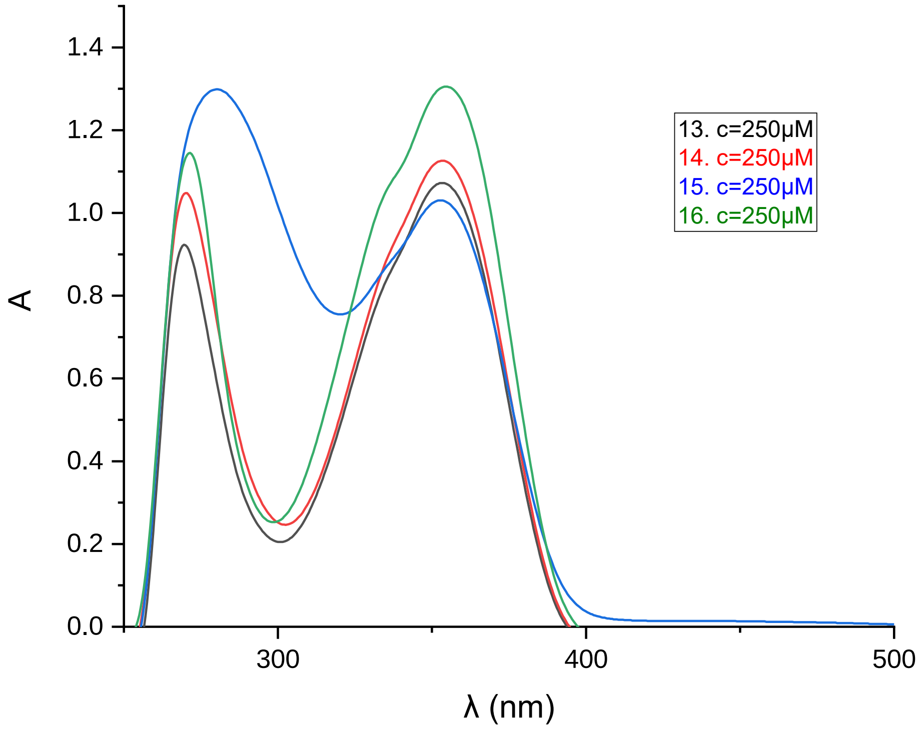
13. c=250µM
14. c=250µM
15. c=250µM
16. c=250µM
A
λ (nm)
0.0
0.2
0.4
0.6
0.8
1.0
1.2
1.4
300
400
500

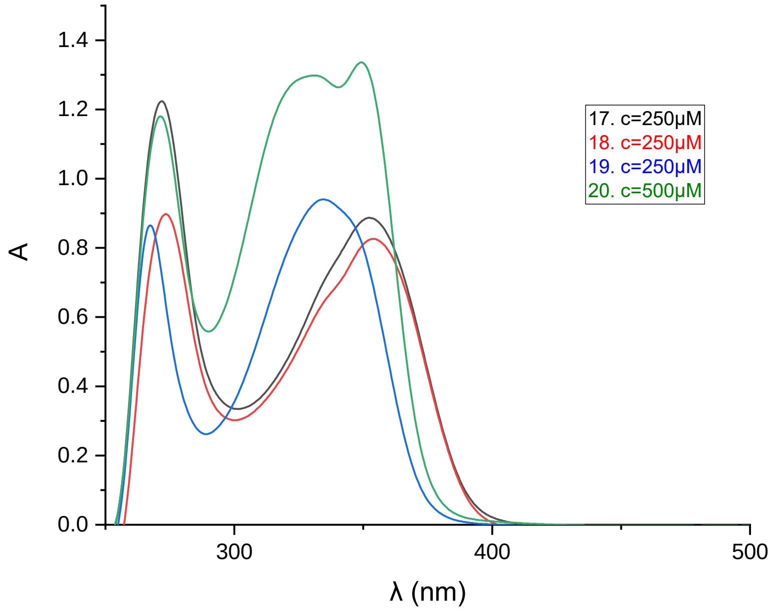

1.4
1.2
1.0
0.8
0.6
0.4
0.2
0.0
A
300
400
500
λ (nm)
17. c=250μM
18. c=250μM
19. c=250μM
20. c=500μM

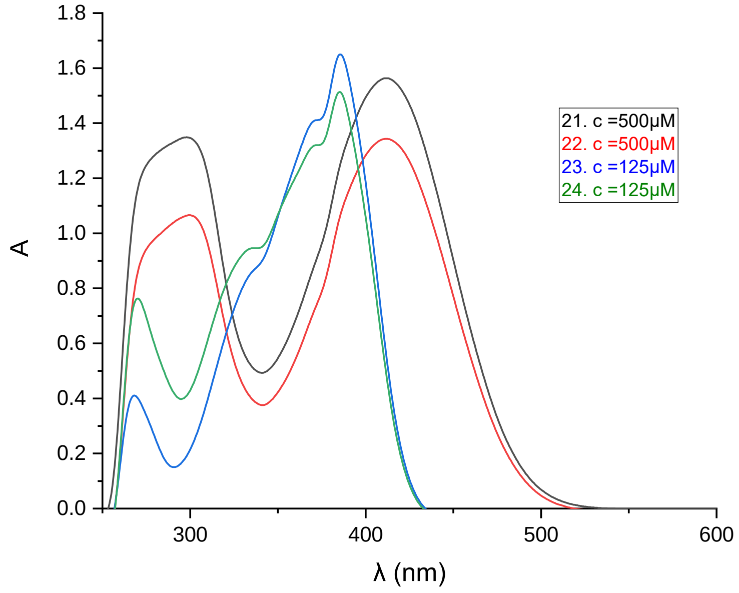

1.8
1.6
1.4
1.2
1.0
0.8
0.6
0.4
0.2
0.0
A
300
400
500
600
λ (nm)
21. c =500μM
22. c =500μM
23. c =125μM
24. c =125μM

S.5. Gel electrophoresis pictures of compounds 1-24 (UV-A)

S.5.1

1 2 3 4 5 6 7 8 9

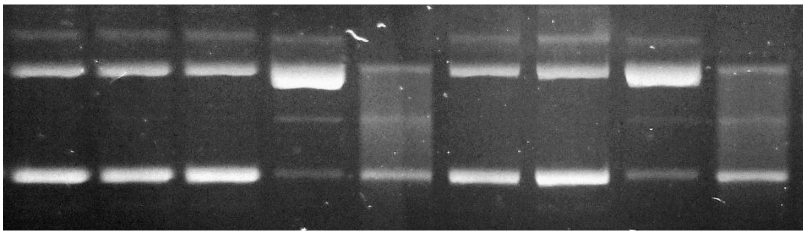

Form II% : 38 33 66 40 38 36 60 37

Form III%: 12 23 14 24

lane	365nm, 10cm, 120min, DMSO, C = 500 μM				
1	DNA + UV +	Control	**lane**		
2	DNA + UV +	**1**	6	DNA + UV +	**1**
3	DNA + UV +	**2**	7	DNA + UV +	**2**
4	DNA + UV +	**3**	8	DNA + UV +	**3**
5	DNA + UV +	**4**	9	DNA + UV +	**4**

Gel S.5.1: Compounds 1-4

S.5.2

1 2 3 4 5 6 7 8 9 10 11 12 13 14 15

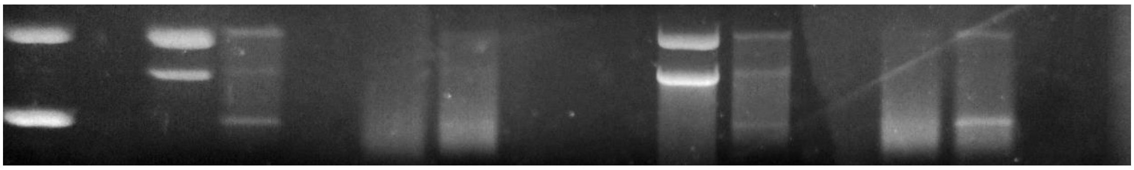

Form II%: 57 49 37 42

Form III%: 29 23 49 31

lane	365nm, 10cm, 120min, DMSO, C = 500 μM				
1	DNA + UV +	Control	**lane**		
2	DNA + UV +	**5**	9	DNA + UV +	**5**
3	DNA + UV +	**6**	10	DNA + UV +	**6**
4	DNA + UV +	**7**	11	DNA + UV +	**7**
5	DNA + UV +	**8**	12	DNA + UV +	**8**
6	DNA + UV +	**19**	13	DNA + UV +	**19**
7	DNA + UV +	**20**	14	DNA + UV +	**20**
8	DNA + UV +	**9**	15	DNA + UV +	**9**

Gel S.5.2: Compounds 5-9 & 19-20

S.5.3

1 2 3 4 5 6 7 8 9 10 11 12 13 14 15

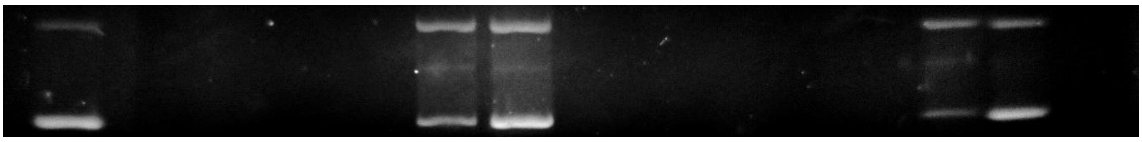

Form II% : 33 30 36 28

Form III%: 27 20 18 9

lane	365nm, 10cm, 120min, DMSO, C = 100 μM				
1	DNA + UV +	Control	**lane**		
2	DNA + UV +	**5**	9	DNA + UV +	**5**
3	DNA + UV +	**6**	10	DNA + UV +	**6**
4	DNA + UV +	**7**	11	DNA + UV +	**7**
5	DNA + UV +	**8**	12	DNA + UV +	**8**
6	DNA + UV +	**19**	13	DNA + UV +	**19**
7	DNA + UV +	**20**	14	DNA + UV +	**20**
8	DNA + UV +	**9**	15	DNA + UV +	**9**

Gel S.5.3: Compounds 5-9 & 19-20

S.5.4

1 2 3 4 5 6 7 8 9 10 11 12 13 14 15

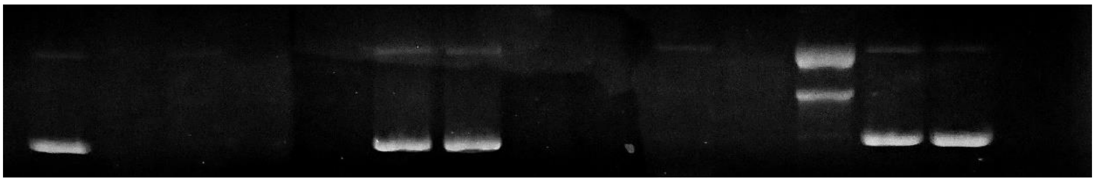

Form II% : 26 25 55 10 5

Form III%: 33

lane	365nm, 10cm, 120min, DMSO, C = 10 μM				
1	DNA + UV +	Control	**lane**		
2	DNA + UV +	**5**	9	DNA + UV +	**5**
3	DNA + UV +	**6**	10	DNA + UV +	**6**
4	DNA + UV +	**7**	11	DNA + UV +	**7**
5	DNA + UV +	**8**	12	DNA + UV +	**8**
6	DNA + UV +	**19**	13	DNA + UV +	**19**
7	DNA + UV +	**20**	14	DNA + UV +	**20**
8	DNA + UV +	**9**	15	DNA + UV +	**9**

Gel S.5.4: Compounds 5-9 & 19-20

S.5.5

1 2 3 4 5 6 7 8 9 10 11 12 13 14 15

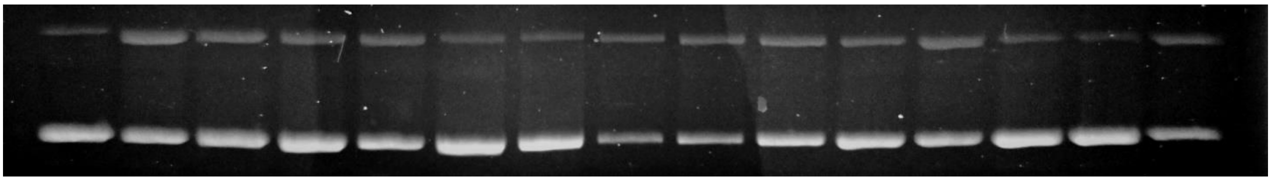

Form II% : 33 27 23 27 19 19 32 33 29 22 31 17 12 24
Form III%:

lane	365nm, 10cm, 120min, DMSO, C = 500 nM				
1	DNA + UV +	Control	**lane**		
2	DNA + UV +	**5**	9	DNA + UV +	**5**
3	DNA + UV +	**6**	10	DNA + UV +	**6**
4	DNA + UV +	**7**	11	DNA + UV +	**7**
5	DNA + UV +	**8**	12	DNA + UV +	**8**
6	DNA + UV +	**19**	13	DNA + UV +	**19**
7	DNA + UV +	**20**	14	DNA + UV +	**20**
8	DNA + UV +	**9**	15	DNA + UV +	**9**

Gel S.5.5: Compounds 5-9 & 19-20

S.5.6

1 2 3 4 5 6 7 8 9 10 11 12 13 14 15

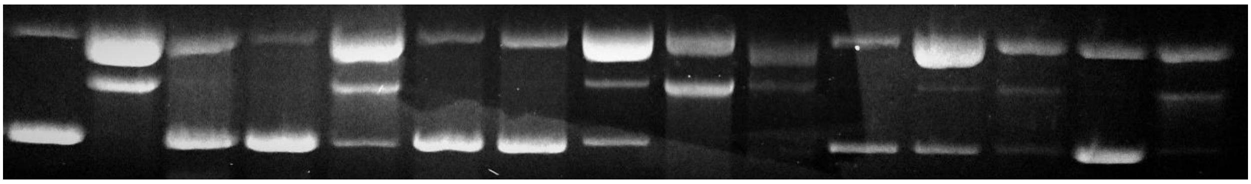

Form II% : 65 36 20 57 30 37 69 44 55 48 75 43 31 64
Form III%: 20 25 10 38 17 7 12 28

lane	365nm, 10cm, 120min, DMSO, C = 10 μM				
1	DNA + UV +	Control	**lane**		
2	DNA + UV +	**10**	9	DNA + UV +	**10**
3	DNA + UV +	**11**	10	DNA + UV +	**11**
4	DNA + UV +	**12**	11	DNA + UV +	**12**
5	DNA + UV +	**13**	12	DNA + UV +	**13**
6	DNA + UV +	**14**	13	DNA + UV +	**14**
7	DNA + UV +	**15**	14	DNA + UV +	**15**
8	DNA + UV +	**16**	15	DNA + UV +	**16**

Gel S.5.6: Compounds 10-16

S.5.7

1 2 3 4 5 6 7 8 9 10 11 12 13 14 15

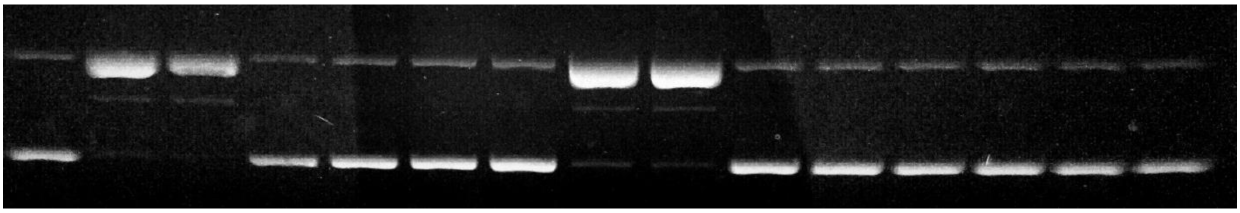

Form II% : 81 75 23 21 23 23 86 84 21 20 21 19 17 16

Form III%: 11 14 9 11

lane	365nm, 10cm, 120min, DMSO, C = 10 µM				
1	DNA + UV +	Control	**lane**		
2	DNA + UV +	**17**	9	DNA + UV +	**18**
3	DNA + UV +	**18**	10	DNA + UV +	**21**
4	DNA + UV +	**21**	11	DNA + UV +	**22**
5	DNA + UV +	**22**	12	DNA + UV +	**23**
6	DNA + UV +	**23**	13	DNA + UV +	**24**
7	DNA + UV +	**24**	14	DNA + UV +	**21** (5 µM)
8	DNA + UV +	**17**	15	DNA + UV +	**22** (5 µM)

Gel S.5.7: Compounds 17-18 & 21-24

S.5.8

1 2 3 4 5 6 7 8 9 10 11

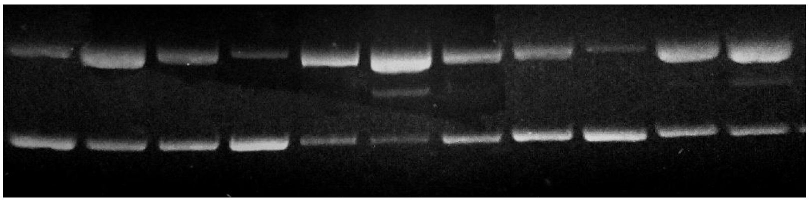

Form II%: 57 39 18 60 64 49 39 21 57 59

Form III%: 15 11

lane	365nm, 10cm, 120min, DMSO, C = 2 µM				
1	DNA + UV +	Control			
2	DNA + UV +	**5**	7	DNA + UV +	**5**
3	DNA + UV +	**6**	8	DNA + UV +	**6**
4	DNA + UV +	**7**	9	DNA + UV +	**7**
5	DNA + UV +	**8**	10	DNA + UV +	**8**
6	DNA + UV +	**9**	11	DNA + UV +	**9**

Gel S.5.8: Compounds 5-9

S.6. Optimized molecular geometries of compounds 1-24 at B3LYP/6-31g* level

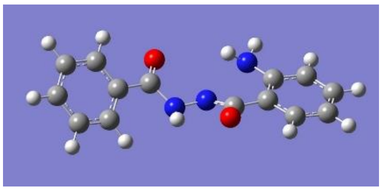
1

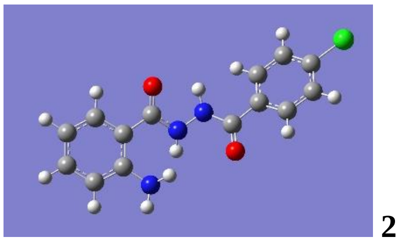
2

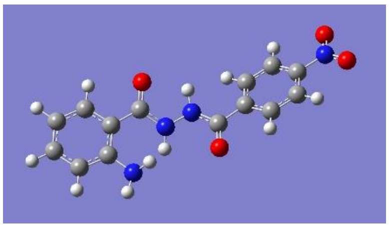
3

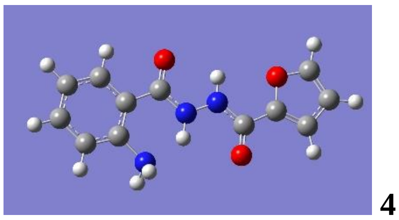
4

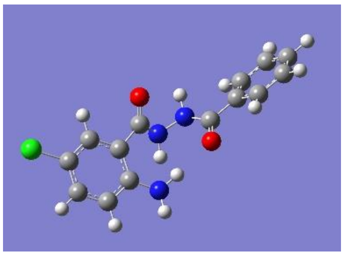
5

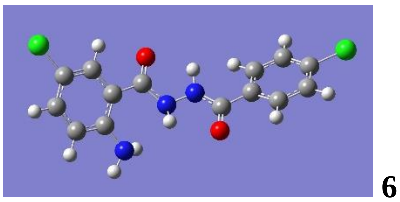
6

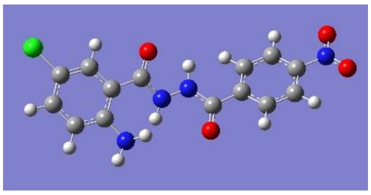
7

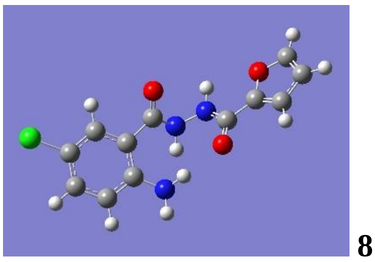
8

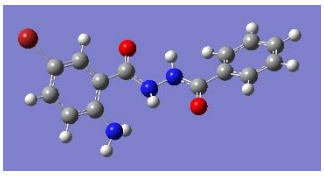
9

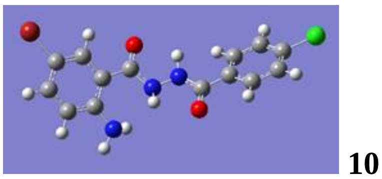
10

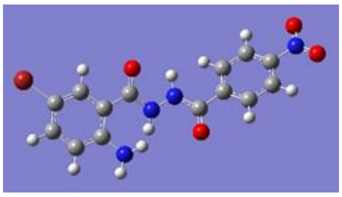
11

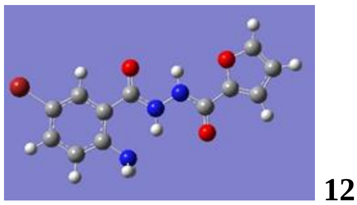
12

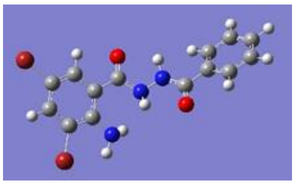
13

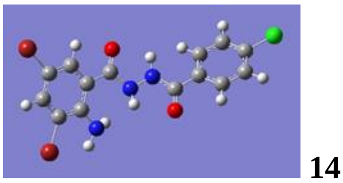
14

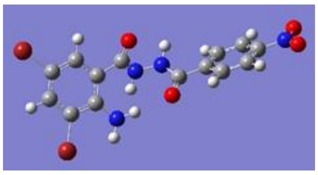
15

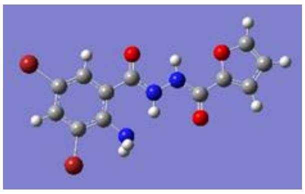
16

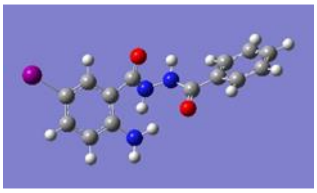
17

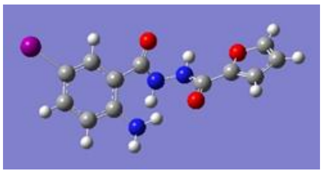
18

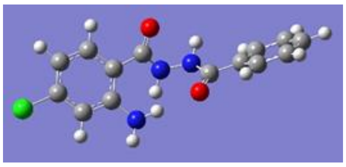
19

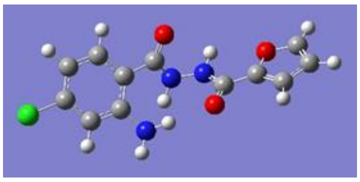
20

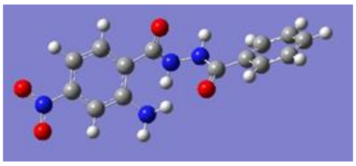
21

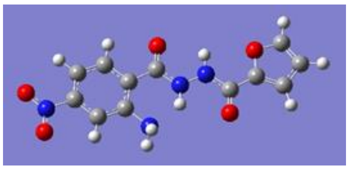
22

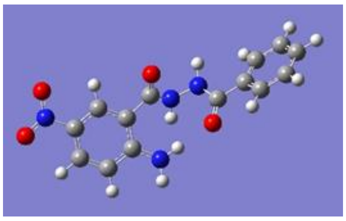
23

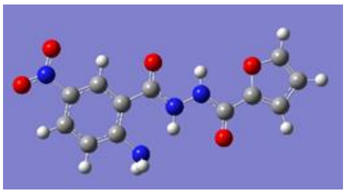
24

S.7. Molecular Docking Studies for the compounds 1-24

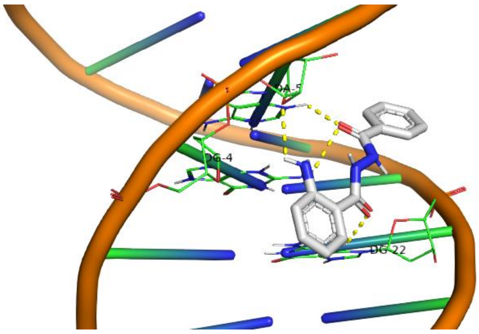

(**1**)

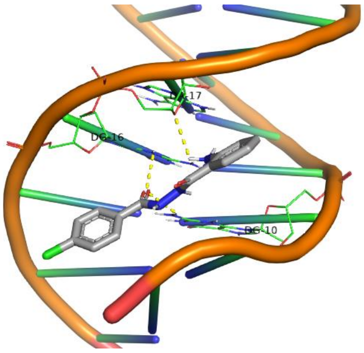

(**2**)

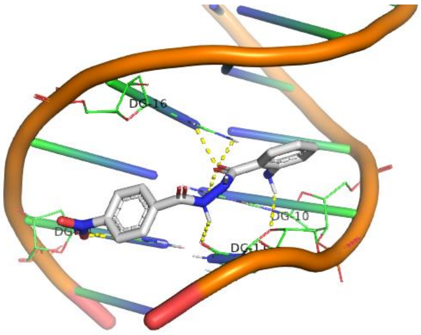

(**3**)

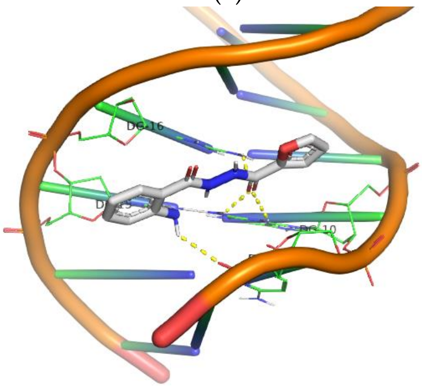

(**4**)

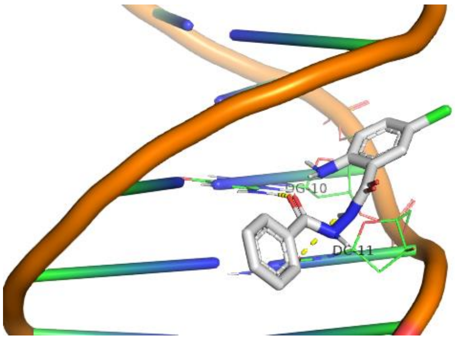

(**5**)

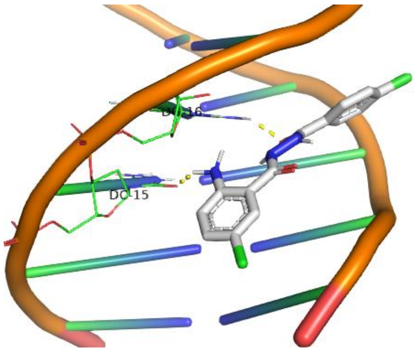

(**6**)

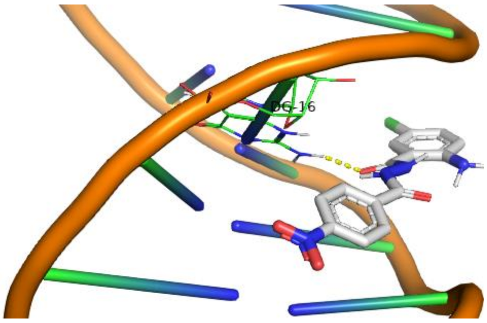

(**7**)

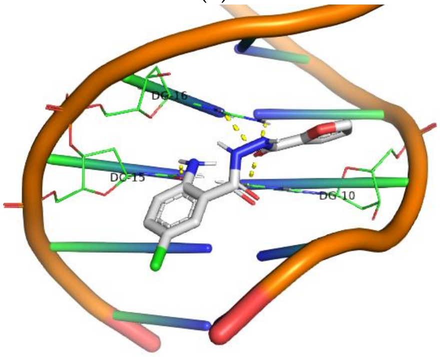

(**8**)

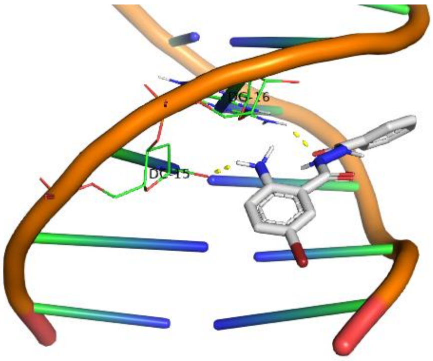

(**9**)

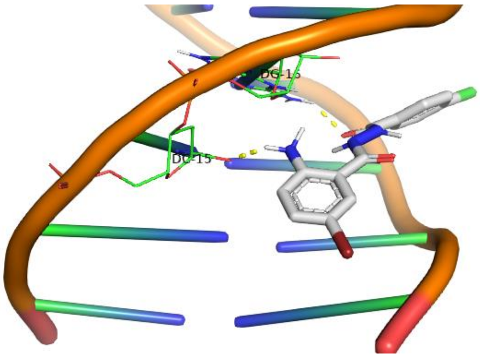

(**10**)

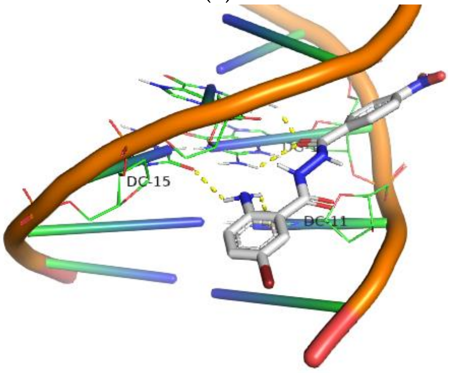

(**11**)

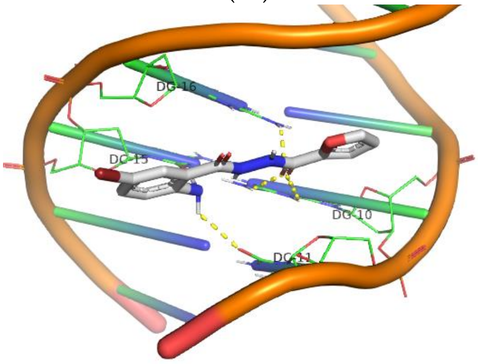

(**12**)

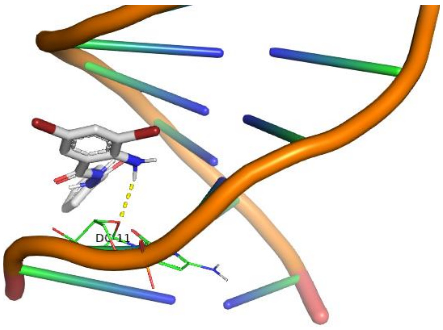

(**13**)

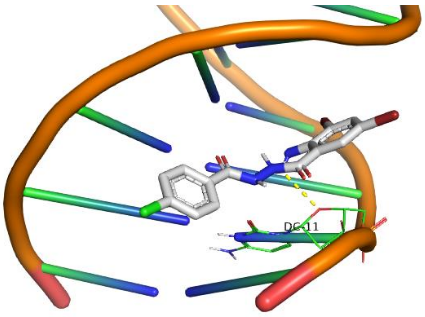

(**14**)

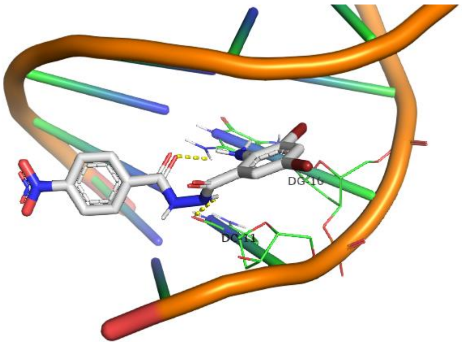

(**15**)

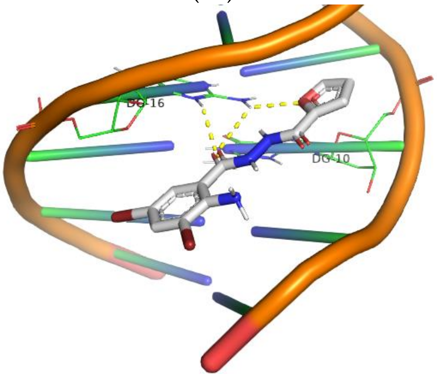

(**16**)

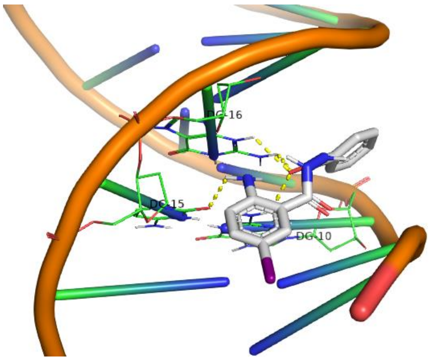

(**17**)

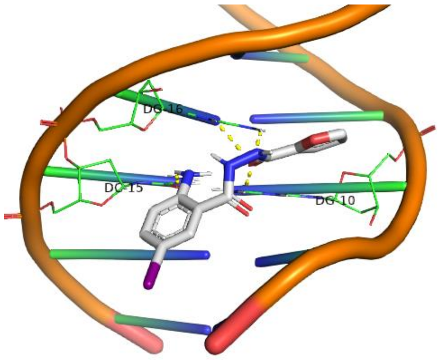

(**18**)

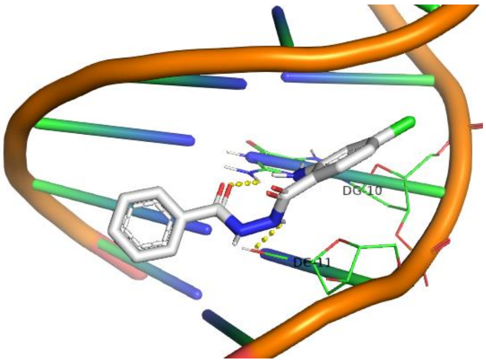

(**19**)

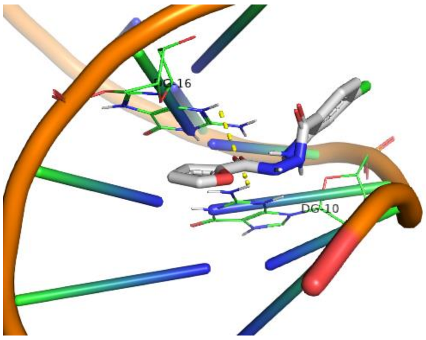

(**20**)

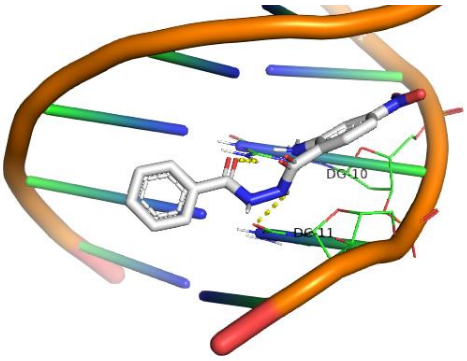

(**21**)

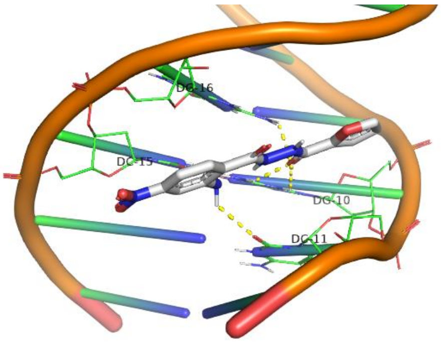

(**22**)

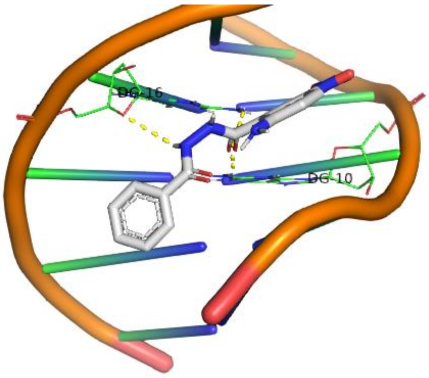

(**23**)

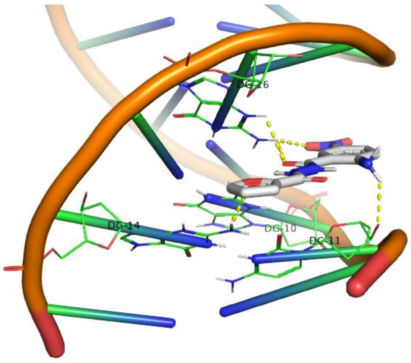

(**24**)
